# Single-nucleus chromatin accessibility profiling highlights distinct astrocyte signatures in progressive supranuclear palsy and corticobasal degeneration

**DOI:** 10.1007/s00401-022-02483-8

**Published:** 2022-08-17

**Authors:** Nils Briel, Viktoria C. Ruf, Katrin Pratsch, Sigrun Roeber, Jeannine Widmann, Janina Mielke, Mario M. Dorostkar, Otto Windl, Thomas Arzberger, Jochen Herms, Felix L. Struebing

**Affiliations:** 1 Center for Neuropathology and Prion Research, University Hospital Munich, Ludwig–Maximilians-University, Feodor-Lynen-Str. 23, 81377 Munich, Germany; 2 German Center for Neurodegenerative Diseases, Feodor-Lynen-Str. 17, 81377 Munich, Germany; 3 Munich Medical Research School, Faculty of Medicine, Ludwig-Maximilians-University, Bavariaring 19, 80336 Munich, Germany; 4 Munich Cluster of Systems Neurology (SyNergy), Feodor-Lynen-Str. 17, 81377 Munich, Germany; 5 Department of Psychiatry and Psychotherapy, University Hospital Munich, Ludwig-Maximilians-University, Nussbaumstr. 7, 80336 Munich, Germany

**Keywords:** Progressive supranuclear palsy, Corticobasal degeneration, Tauopathy, snATAC-seq, Astrocytes, Neurodegeneration

## Abstract

Tauopathies such as progressive supranuclear palsy (PSP) and corticobasal degeneration (CBD) exhibit characteristic neuronal and glial inclusions of hyperphosphorylated Tau (pTau). Although the astrocytic pTau phenotype upon neuropathological examination is the most guiding feature in distinguishing both diseases, regulatory mechanisms controlling their transitions into disease-specific states are poorly understood to date. Here, we provide accessible chromatin data of more than 45,000 single nuclei isolated from the frontal cortex of PSP, CBD, and control individuals. We found a strong association of disease-relevant molecular changes with astrocytes and demonstrate that tauopathy-relevant genetic risk variants are tightly linked to astrocytic chromatin accessibility profiles in the brains of PSP and CBD patients. Unlike the established pathogenesis in the secondary tauopathy Alzheimer disease, microglial alterations were relatively sparse. Transcription factor (TF) motif enrichments in pseudotime as well as modeling of the astrocytic TF interplay suggested a common pTau signature for CBD and PSP that is reminiscent of an inflammatory immediate-early response. Nonetheless, machine learning models also predicted discriminatory features, and we observed marked differences in molecular entities related to protein homeostasis between both diseases. Predicted TF involvement was supported by immunofluorescence analyses in postmortem brain tissue for their highly correlated target genes. Collectively, our data expand the current knowledge on risk gene involvement (e.g., *MAPT*, *MAPK8*, and *NFE2L2*) and molecular pathways leading to the phenotypic changes associated with CBD and PSP.

**Supplementary Information:**

The online version contains supplementary material available at 10.1007/s00401-022-02483-8.

## Introduction

Most neurodegenerative disorders are characterized by misfolded, intracellular protein aggregates that can disrupt neuronal and glial homeostasis. Among these, tauopathies represent a group of diseases in which deposits of hyperphosphorylated tau (pTau) protein can be seen upon neuropathological examination [25]. Examples of tauopathies are Alzheimer’s disease (AD) and Progressive Supranuclear Palsy (PSP) as well as the less common Corticobasal Degeneration (CBD). Because clinical symptoms of those three diseases can overlap, definite diagnosis requires postmortem neuropathological examination [2, 38, 64]. Besides the distribution pattern of pTau inclusions throughout the central nervous system, two histological features are used to distinguish tauopathies: affected cell types and the immunohistochemical ratio of isoform-specific Tau antibodies [18, 64].

**Fig. 1:**
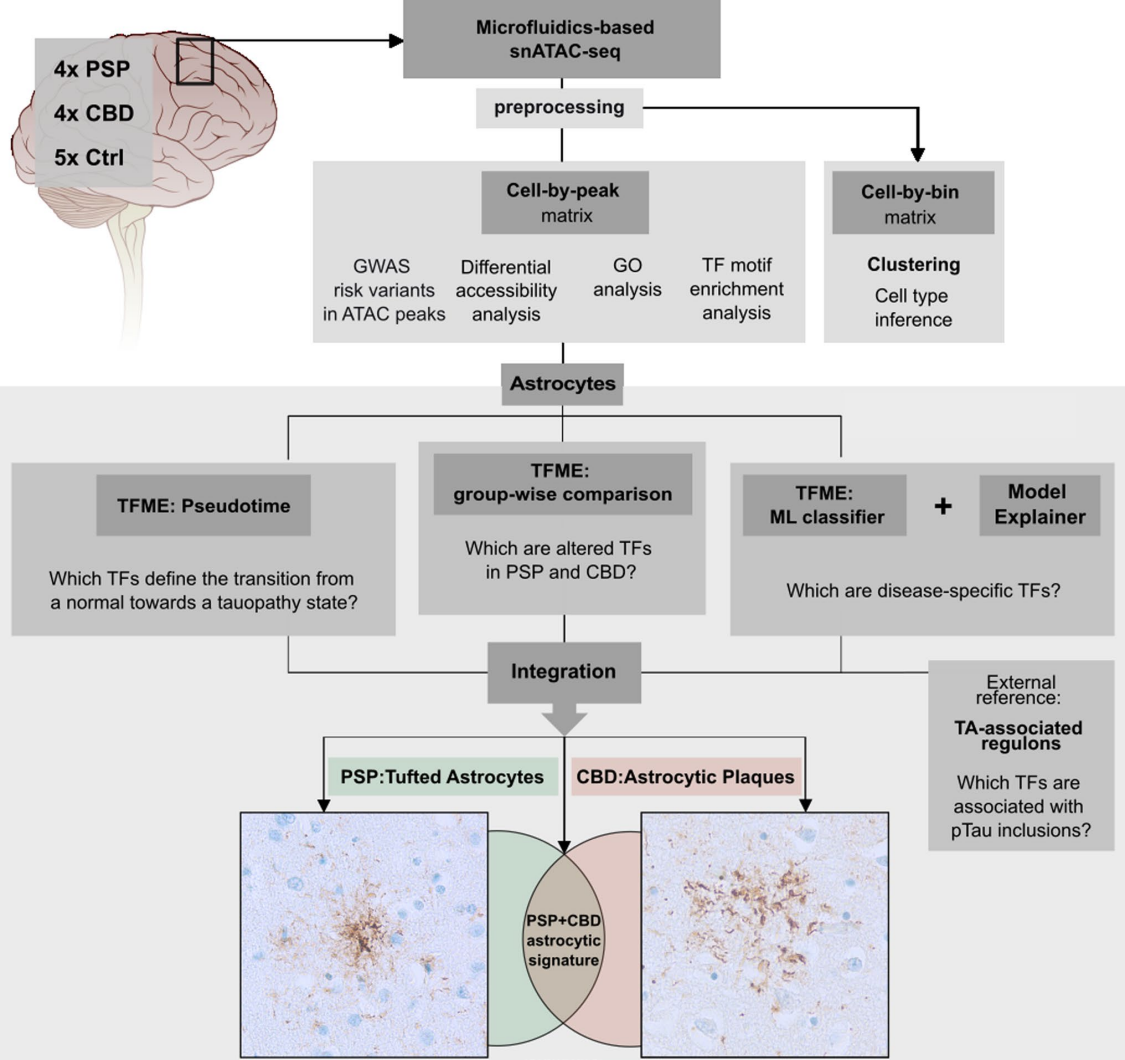
Concept of the bioinformatical analysis. SnATAC-sequencing was applied to snap-frozen frontal cortex samples from deceased PSP, CBD, and Ctrl individuals. Raw sequencing reads were preprocessed and resulting matrices were then used (i) for graph-based clustering and cell type inference (using a binned genome), and (ii) for GWAS risk variant-association with cell types, differential accessibility analysis and GO, as well as TF-motif analysis (using the peak matrix). Downstream, only the astrocytic cluster was investigated (boxed lower part). To find significantly altered TFs in tauopathy-derived astrocytes, disease-wise comparisons of TFME were conducted (mid panel). TFME changes along pseudotime trajectories were assessed to identify TFs linked with pathogenesis (left). An ML-based disease classifier was utilized to delineate disease-specific TFs in a more unbiased approach (right). Significant results from these three branches were refined by a TF profile linked to the presence of astrocytic pTau inclusions in PSP (Tufted Astrocytes, TA). Finally, this multilayered regulon pattern was integrated to define a general astrocytic tauopathy TF signature, or entity-specific astrocytic TF signatures. These are presumed to mirror the neuropathological context of characteristic pTau inclusions in astrocytes, namely TA in PSP and AP in CBD. *AP* astrocytic plaque, *GO* gene ontology, *GWAS* genome wide association studies, *ML* machine learning, *pTau* hyperphosphorylated Tau, *TA* tufted astrocyte, *TFME* transcription factor motif enrichment. The brain illustration was modified from https://de.m.wikipedia.org/wiki/Datei:Brain_stem_normal_human.svg (CC-Attribution-2.5 License 2006)

In regard to the latter, the Tau harboring gene *MAPT*, located on chromosome 17q21.23, gives rise to six isoforms by differential splicing involving exons 2, 3, and 10 [18, 21]. The microtubule-binding domain, consisting of 3 or 4 repeats (3R/4R) depending on inclusion of exon 10, not only defines the affinity of Tau to microtubules, but also its aggregation properties [25]. While AD can be regarded a mixed 3R/4R tauopathy, 4R isoforms predominate in PSP and CBD. As for affected cell types, AD, PSP, and CBD all share neuronal pTau inclusions such as neurofibrillary tangles or neuropilic threads. Glial inclusions are rare in AD, but common in CBD or PSP [16]. The most prominent immunoreactive feature to discriminate PSP from CBD is the astrocytic pTau phenotype: with tufted astrocytes (TA) being a hallmark for PSP and astrocytic plaques (AP) for CBD as shown in Fig. 1 [11, 38, 64].

Postmortem transcriptome studies can help in identifying disease-associated signatures and, when performed in single-cell resolution, even quantify the degree of cell type-specific involvement. This has been achieved primarily in the context of AD, and through integration of multiple “omics” studies such as single-cell and genome-wide association studies (GWAS), it is now believed that microglia are key players in AD pathogenesis [22, 40, 46, 54]. This insight is already being translated in a therapeutic context, with studies underway that seek to restore microglial fitness [26]. In contrast, postmortem “omics”-studies are sparse in PSP and CBD [1, 7, 22, 24, 34], and to date non-existent in single-cell resolution.

The lack of cell type-resolved molecular data for PSP and CBD compelled us to perform this study. We hypothesized that their neuropathological phenotype would be mirrored by molecular changes in specific cell types, and we wondered whether we could find molecular features that distinguished both diseases. We chose an epigenetic assay—the single-nucleus assay for transposase accessible chromatin sequencing (snATAC-seq) as compared to a transcriptomic assay (snRNA-seq). The read-out of snATAC-seq corresponds to regions of open chromatin in the genome, and these are commonly associated with loci of active transcription or epigenetic regulation [8, 33]. This assay relies on DNA instead of RNA as input material; hence, degradation of RNA, which is commonly associated with long postmortem intervals and agony phases, is not a cause of concern. Furthermore, since the coordinated expression of genes has been shown to be regulated by effects of transcription factors (TFs) on the compaction of chromatin [33, 60], altered TF dynamics might have an impact on pathogenesis in these diseases. This can be explored optimally with an open chromatin assay such as snATAC-seq. Direct TF effects are usually guided by DNA sequence motifs, so-called TF binding sites, which are located in non-coding *cis-*regulatory elements (CREs) like enhancers, silencers, or promoters [57]. Their interaction is perturbed in diseases with a complex genetic background [31], probably explaining the large contribution of non-coding mutations to pathogenesis. More precisely, mutations in CREs can affect TF binding dynamics to varying degrees [12]. Thus, viewed from a higher perspective, the TF interplay within a given cell type can be regarded as an integration point of genetic background, epigenetic information, and as effector of intra- and extracellular signaling pathways. Because TF binding sites within CREs outnumber genes and their transcripts by several orders of magnitude, the feature space is larger in snATAC-seq compared to snRNA-seq data, which can be leveraged for increased discriminatory power.

Figure 1 outlines our discovery-driven approach: first, we demonstrate the importance of astrocytes to CBD and PSP disease pathology by combining tauopathy-associated genes and GWAS risk variants with our data. Then we explore their transition into a tauopathy state via TF profiling. Incorporating separate analyses such as pseudotemporal imputations, group-wise comparisons of TF information, external data, and machine learning models, we ultimately attempt to delineate disease-specific astrocytic TF signatures in a comprehensive data integration part. To validate these findings, we finally detect protein expression alterations of highly correlated target genes in archival brain tissue of PSP and CBD cases using an immunofluorescent staining approach.

## Materials and methods

### Neuropathological assessment and case selection

The complete brain was prepared at autopsy. Hemispheres were treated differently: the left hemisphere was fixed in formalin for a duration of two weeks or longer before coronal slicing of 1 cm step size, while the right hemisphere was snap-frozen immediately. From the former, paraffin-embedded specimen sampled across the whole cerebrum, brain stem, cerebellum, and spinal cord were used for diagnostic examination.

Cases (4 PSP, 4 CBD) and controls (5 Ctrls) were selected based on the amount of additional co-pathology and matched by age (PSP 72.8 ± 4.8, CBD 56.5 ± 2.9, Ctrl 69.4 ± 14), postmortem interval (PSP 41.2 ± 34.2, CBD 33.8 ± 20.2, Ctrl 26.2 ± 6.8), and sex (males, PSP = 50%, CBD = 50%, Ctrl = 60%) at its best (see Table 1). Ctrl cases were chosen based on the absence of neurologic or psychiatric disease history. Those Ctrl or tauopathy cases exhibiting substantial co-pathology (i.e., Aβ_42_, α-synuclein, TDP-43, pTau (AT8), Aging-related Tau-Astrogliopathy (ARTAG), Primary Aging-related Tauopathy (PART), 3R Tau (RD3), or 4R Tau (RD4; in case of Ctrl)) in study-relevant regions were excluded.

**Table 1:** Covariates of PSP, CBD, and Ctrl subjects

Case ID	Neuropathological diagnosis	Neurological diagnosis (last *ante mortem*)	Age at death [years]	PMI [hours]	Sex	Disease duration [years]	Brain weight [g]	Tau isoform^#^ [IHC: 4R/3R]	AGD	Braak&Braak (NFT)	CERAD-plaque density	Thal-Phase		Aβ42 (4G8)	α-synuclein (Braak; LBs)	TDP-43	FUS
PSP1	PSP	PSP-RS	68	38	Male	6.0	1460	±	−	1	0	0	No		0	neg	neg
PSP2	PSP	PSP-ns	77	78	Female	2.5	1112	±	−	0	0	0	No		0	neg	neg
PSP3	PSP	PSP-ns	78	42	Male	9.0	NA	±	+	1	0	1*	Yes*		0	neg	neg
PSP4	PSP	bvFTD-tauopathy	68	7	Female	7.0	1005	±	−	0	0	0	No		0	neg	neg
CBD1	CBD	PSP-RS	52	14	Female	4.5	1260	±	−	0	0	0	No		0	neg	neg
CBD2	CBD	bvFTD	56	44	Male	2.5	NA	±	+	1	0	0	No		0	neg	neg
CBD3	CBD	bvFTD-CBS	59	23	Female	7.0	1220	±	−	1	–	1*	Yes*		0	pos	neg
CBD4	CBD	bvFTD-MND	59	54	Male	1.5	1330	±	+	1	0	3*	Yes*		0	neg	neg
C1	Ctrl	−	58	22	Male	−	NA	−/−	−	1	−	0	−		0	pos	−
C2	Ctrl	−	82	27	Male	−	1230	−/−	−	2	−	1*	−		0	neg	neg
C3	Ctrl	−	86	20	Female	−	1035	−/−	−	1	−	0	−		0	−	−
C4	Ctrl	−	64	33	Female	−	NA	−/−	−	1	−	0	−		0	neg	neg
C5	Ctrl	−	53	29	Male	−	1792	−/−	−	0	−	2*	−		0	neg	neg

*Aβ42* amyloid beta 42, *AGD* Argyrophilic Grain Disease, *bvFTD* behavioral variant Frontotemporal Dementia, *CBS* Corticobasal Syndrome, *CERAD* Consortium to Establish a Registry for Alzheimer's Disease, *Ctrl* control, *IHC* immunohistochemistry, *LBs* Lewy bodies, *MND* motor neuron disease, *NA* not assessable, *neg* negative, *NFT* neurofibrillary tangles, *ns* not specified, *PMI* *postmortem* interval, *RS* Richardson Syndrome. See also comprehensive data (Supplementary Data, T0, online resource)

^#^In study-relevant region. *No 4G8/A*β42-*positivity or A*β-*plaques in the study-relevant region

### Region of interest

Approximately 1 cm^3^-thick tissue blocks of grey and appending white matter were excised from snap-frozen coronal cerebral slices using a diamond band saw. The regions of interest were parts of the medial and superior frontal gyrus at the level of the anterior striatum (MFG, SFG) in the coronal view corresponding to Brodmann areas 6/8/9. The corresponding regions were sampled from the contralateral formalin-fixed hemisphere for validation studies.

### Single-nucleus ATAC-sequencing

#### Nuclei preparation and quantification

To isolate nuclei, 200 mg of brain tissue was homogenized in 3.75 mL of chilled lysis buffer (10 mM Tris–HCl pH 8.0, 0.32 M sucrose, 0.34 mM DTT, 0.1 mM PMSF, 3 mM MgAc_2_, 5 mM CaCl_2_, 0.1 mM EDTA, 0.1% Igepal, and 1 protease inhibitor cocktail tablet (complete mini protease inhibitor cocktail, Roche Diagnostics, Mannheim, Germany) per 50 mL) using a Dounce homogenizer and transferred to 15 mL-ultracentrifugation tubes (Seton Open-top polyallomer centrifuge Tubes) with additional 2.25 mL lysis buffer. Homogenates were underlaid with 6.75 ml sucrose buffer (10 mM Tris–HCl, pH 8.0, 1.8 M sucrose, 0.34 mM DTT, 0.1 mM PMSF, 3 mM MgAc_2,_ and 1 protease inhibitor cocktail tablet per 50 mL) and centrifuged for 1 h at 24,000 rpm at 4 °C. The nuclei pellet was resuspended in 1 × nuclei buffer (10 × Genomics) and nuclei were quantified using a Neubauer haemocytometer.

### Single nuclei partitioning and snATAC-Seq library construction

Single nuclei partitioning and subsequent snATAC-Seq library construction were performed using the Chromium Next GEM Single Cell ATAC Reagent Kit v1.1 (10 × Genomics, Pleasanton, CA, USA) according to the manufacturer’s protocol. Briefly, following incubation with ATAC Enzyme for 1 h at 37 °C, nuclei were loaded onto a Chromium Next GEM Chip H for a targeted recovery of 5000 or 10,000 nuclei per PSP/CBD and Ctrl sample, respectively. After partitioning of the nuclei and DNA cleanup, sample indices were added, and double-sided size selection was performed. Finally, libraries were eluted in 20 µl Buffer EB (Qiagen, Hilden, Germany) and stored at − 20 °C. Correct fragment size distribution of the libraries was checked via the Agilent Bioanalyzer System using an Agilent bioanalyzer High-sensitivity DNA chip (Agilent, Santa Clara, CA, USA). Library concentrations were determined using the KAPA library Quantification Kit for Illumina Platforms (Roche Diagnostics, Mannheim, Germany).

### Sequencing of snATAC-seq libraries

Quantified and quality-controlled snATAC libraries were pooled at equimolar concentrations, denaturized, and sequenced on an Illumina NovaSeq6000 platform according to the 10 × Genomics sequencing requirements for single-indexed snATAC-Seq libraries, aiming at a minimum sequencing depth of 25,000 read pairs per nucleus.

### Analysis of snATAC-seq data

The main bioinformatical workflow is illustrated in Fig. 1 and consists of 8 parts (a detailed description of bioinformatical analyses is included separately as a Supplementary Methods section, online resource). Initially, sequencing data were subjected to the 10 × Genomics™ *cellranger-atac-1.2.0* pipeline and *Snaptools/SnapATAC* packages [14] for preprocessing and quality control (QC), respectively. Barcodes were filtered for mapping quality, fragment sizes, and correct alignment flags. *SnapATAC’s* representations of chromatin accessibility as either bins (equally sized genomic windows of 1,000 bp overlapping with sequenced DNA-fragments) or peaks (exact genomic ranges of cluster-aggregated DNA-fragments) were the basis for all downstream single-nucleus analyses. Gene accessibility (GA) as a surrogate of a gene’s transcriptional activity was calculated as a *z* score-based metric in *SnapATAC* [14].

The cell-by-bin matrix was used for clustering (Supplementary Fig. 1, online resource) and barcode embedding using uniform manifold approximation and projection (UMAP) metrics. Technical covariates were identified, and batch effect correction applied to the primary UMAP embedding (Supplementary Figs. 1 and 2, online resource). Furthermore, graph-based cell type inference was conducted in the UMAP embedding, while RNA- and ATAC-seq data-derived marker gene lists from McKenzie et al. [41] and Lake et al. [35] served as references (Supplementary Figs. 3 and 4, Supplementary Data, T01, online resource).

The cell-by-peak matrix was leveraged for identifying GWAS risk variants in ATAC peaks [28], differential gene accessibility analysis, gene ontology (GO) analysis, and TF motif enrichment (TFME) analysis*.* To assess cell type enrichment of GWAS risk variants, publicly available disease-specific GWAS [24, 34] summary statistics were downloaded from https://www.ebi.ac.uk/gwas/ for PSP (Orphanet_683), CBD (Orphanet_278), AD (EFO_0000249), Frontotemporal Dementia (FTD, Orphanet_282), Parkinson Disease (PD, EFO_0002508), Multiple System Atrophy (MSA, EFO_1001050), Lewy Body Dementia (LBD, EFO_0006792), and Amyotrophic Lateral Sclerosis (ALS, EFO_0000253).

Quantification of alterations assigned to biological pathway terms was enabled by the *amiGO2* database (http://amigo.geneontology.org/amigo/search/bioentity) filtered for the terms ‘chaperon-mediated autophagy’ (CMA), ubiquitin–proteasome-system (UPS), and unfolded-protein-response (UPR) or ‘microglial cell activation’ in *Homo sapiens*.

The subsequent steps were exclusively conducted with the astrocytic cluster: first, we assessed group-wise differences of TFME using *Wilcoxon* rank-sum tests and the *Bonferroni* method to adjust for multiple hypothesis testing.

Employing the package *Cicero* [49]*,* we constructed pseudotime trajectories on the re-embedded astrocyte cluster, which was filtered for Ctrl- and CBD-derived astrocytic nuclei. High TFME levels of an epigenetic indicator of astrocytic immaturity (i.e., the TF EMX2) served to define the origin of the trajectory. To evaluate GA and TFME changes along these trajectories, *tradeSeq* [3]*,* its modeling framework, and *Wald-*test-based functions were used.

To identify features that are most distinctive in predicting the group entity (Ctrl, PSP, or CBD), a supervised machine learning (ML) algorithm called extreme gradient boosting tree (XGB) was trained on the astrocytic TFME. Train-test set splits consisted of 80% or 20% of the complete astrocytic population, respectively. The model’s predictive performance was primarily measured by *overall accuracy* and *Cohen’s kappa* in the test set; further classification performance indicators (e.g., sensitivity, specificity, negative and positive predictive values) were reported for a more detailed characterization. To interpret the model’s predictive process and to weigh its input features (i.e., TFs) by their importance for a particular prediction, the ML explanation framework *Lime* was used [52].

We identified TFs associated with the classical neuropathological phenotype in PSP (tufted astrocyte, TA) by applying the *Reconstruction of Transcriptional Regulatory Networks.* To this end, we utilized a public transcriptomic data set derived from temporal cortices of 176 PSP cases [1]. Using gene-set enrichment analysis (GSEA), the inferred transcriptional regulatory network was integrated with differentially expressed genes as well as covariate-adjusted neuropathology-gene expression correlation coefficients, which both were reported in the original study. The unit of a given TF with all the genes regulated by it was termed *regulon.*

To ultimately distill a set of shared and disease-specific pTau-associated TF candidates, we integrated the results of all previous analyses. These multidimensional overlaps were visualized as upset plots.

The Supplementary Methods, online resource, offer a more detailed description of the bioinformatical workflow.

### TF target gene validation—bioinformatics, immunofluorescence staining and data analysis

To retrieve the most important target genes of JUNB and TFEB TFs in this astrocyte dataset, correlations between TFME and the GA of all genes in the dataset were computed group-wise. Significant correlations in the Ctrl data were excluded in the following step to reduce false positive, thus unspecific findings. Next, potential target genes were ranked per disease by *BH*-adjusted *p* values and *Pearson* R before searching for genomic overlaps of *Cicero CRE* links and each specific gene locus (± 10 kb). Visual inspection of co-accessibility plots at highly correlated and overlapping gene loci guided identification of the top candidate genes.

Ten micrometer-thick sections were prepared from formalin-fixed paraffin-embedded brain samples of the MFG, which were deparaffinized with Histoclear (VWR Life science, #H103-4L) and rehydrated in descending ethanol series. Antigen retrieval was performed with 1 × Sodium citrate solution, pH 6 in a pressure cooker for 20 min. Autofluorescence quenching/photobleaching was applied using 25,000 Lux LED lights for 2 × 45 min with slides submerged in quenching solution (4.5% H_2_O_2_ and 20 mM NaOH in 1X PBS). Blocking was performed with 5% Goat Serum (GS) and 0.3% Triton X-100 in 1X PBS. Primary antibodies raised in rabbit against the candidate gene products MAP3K8 (1:500, abcam, #ab137589), or CTSD (1:200, abcam, #ab75852) were incubated together with mouse anti-AT8 (1:500, ThermoFisher Scientific, #MN1020) and guinea pig anti-GFAP (in combination with MAP3K8, 1:500, SynapticSystems, #173,004) or chicken anti-GFAP (in combination with CTSD, 1:500, EMD Millipore AB5541), respectively, over night at 4 °C. After washing, secondary antibody incubation followed (1:1000, goat anti-guinea pig /anti-chicken AlexaFluor®488, anti-mouse AlexaFluor®568, anti-rabbit AlexaFluor®647) with an incubation period of 60 min. Finally, slides were covered with mounting medium containing DAPI (#S302380-2, Agilent Dako, Germany) and #1.5H high-precision imaging coverslips.

Images were acquired from the MFG cortex with a Leica Stellaris 5 confocal microscope. pTau + astrocytes were identified based on the prototypical morphology and cellular distribution of aggregates. TAs were defined by bush-like, soma-proximally arranged pTau conglomerate with processes of different thickness. APs were defined by corona-like, soma-distantly arranged fine pTau processes. Then, z-stacks were acquired, using an HC Plan Apochromat CS2 63x/NA 1.40 Oil objective (2048 × 2048 pixels, 181.93^2^ μm height/width, 8–9 μm depth). Images were preprocessed including min and max pixel value cut-offs using custom macro scripts (see also GitHub repository).

Marker-positive (CTSD + , MAP3K8 +) and -negative astrocytes with detectable signal after standardized thresholding were counted using the *CellCounter* tool provided with ImageJ/Fiji. Ratios of GFAP + [Marker] + and GFAP^only^ + cells were compared with ratios of AT8 + (TA/AP) [Marker] + and AT8^only^ + cells.

### General statement: computing environment and statistics

Preprocessing was run in RStudio Server with R3.6 and Python2.7 for Debian Server. The subsequent bioinformatical analysis including statistical testing was conducted within RStudio Desktop running on R3.6.3 (*SnapATAC*) and R4.0.4 (remaining analyses) for Linux (Ubuntu 20.04 LTS). To determine the applicability of hypothesis testing methods, the *Shapiro–Wilk* method was used to test for normal distributions. Consequently, *Welch's* *t-*test was used in normally distributed and *Wilcoxon's* rank-sum test in non-normally distributed populations. *Welch* and *Wilcoxon* tests were conducted as two-tailed versions, unless otherwise stated. For multiple comparison correction, the *BH* method to obtain the false discovery rate (FDR), or the *Bonferroni* method to report family-wise errors were applied to unadjusted *p* values. Significant distribution differences as estimated by the *tradeSeq*-gene models along pseudotime were determined based on their *Wald*-statistic and corrected according to the *Bonferroni* method, unless otherwise stated.

## Results

### Characterization of PSP and CBD frontal lobe brain nuclei via snATAC-seq

**Fig. 2:**
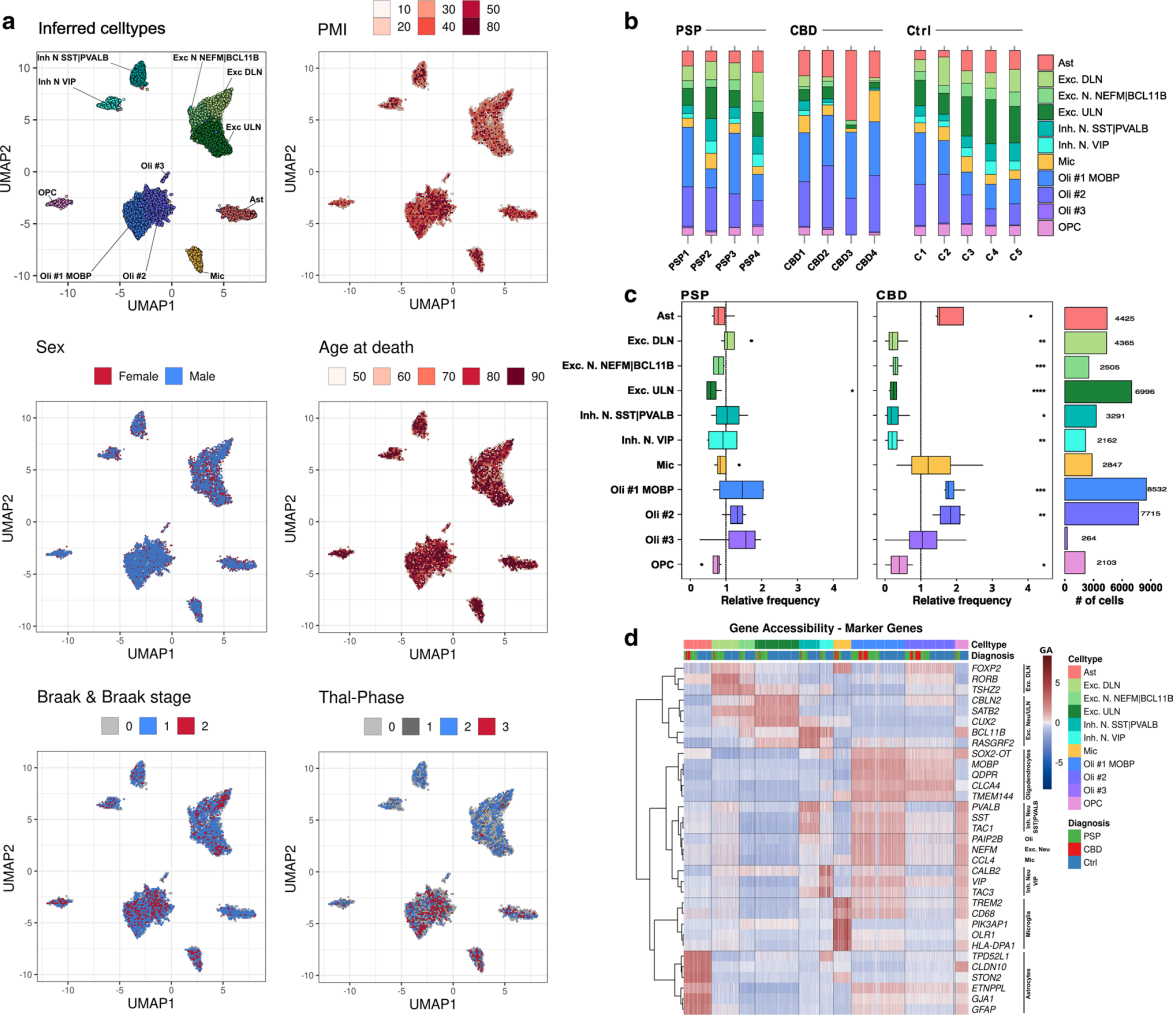
Cell type inference and shifted cell type proportions in primary tauopathies. **a** Projections of cluster-cell type assignments and metadata onto the UMAP embedding of barcodes indicating their dissimilarity in distance between single barcodes (nuclei) as well as the respective variable as color code. Color coding and labels indicate the cell type or sub-cell type identity where applicable. The case-related covariates *postmortem* interval (PMI), sex, age at death, *Braak&Braak* stages did not overtly influence the embedding. Neuronal clusters were rather composited of nuclei originating from cases with *Thal* phase 0–2. Hex-binning was used to visualize overlapping puncta as pixel-wise means in case of PMI, sex, age at death, *Braak&Braak* stages, and *Thal* phases. **b** Bar plots representing relative sample-wise cell type frequencies of PSP, CBD, and Ctrl samples. Color coding indicates the cell type identity. CBD cases exhibit higher relative numbers of astrocytes (esp. CBD3). **c** Boxplots of relative cell type frequencies show excitatory neuron loss in PSP (left) and reductions in all neuronal populations with higher oligodendrocyte frequencies in CBD (mid) samples, when compared to the Ctrls’ mean (vertical dashed line). Outliers are depicted as black dots. The hinges of each box correspond to the 25th and 75th percentiles with medians drawn as black bar. The 1.5-times inter‐quartile ranges are shown as black whiskers. Total numbers of cells (# of cells) are indicated as bar plots on the right. Color coding indicates the cell type identity where applicable, while asterisks display the degree of significance with *p < .05, **p < .01, ***p < .001, and ****p < .0001. **d** Heatmap of gene body accessibility (GA) scores at marker gene loci to guide cell type identification. Every column corresponds to a single barcode, every row to a gene. Color shading indicates the extent of GA from low (blue) to high (red). Rows were clustered hierarchically (*Euclidean* distance, *Ward-D2* method) and results depicted as dendrogram on the left. Cell/barcode order was fixed, but the overlay informs about the definitive cell type and the neuropathological diagnosis. Gene names comply with the *Ensembl* gene IDs. *Exc. DLN* excitatory deep-layer neurons, *Exc. ULN* excitatory upper-layer neurons, *Inh. N.* inhibitory neurons, *Mic* microglia, *Oli* oligodendrocytes, *OPC* oligodendrocytic precursor cells, *PMI* *postmortem* interval

Isolated nuclei from fresh-frozen frontal cortex tissue from 13 samples underwent single-nucleus library preparation and short-read sequencing, yielding open chromatin profiles for 45,205 nuclei. After quality control and dimensionality reduction, clusters were defined from the snATAC-seq data to assess cell type identity (Fig. 2a). The total number of clusters and their respective sizes were found to be robust to downsampling, indicating appropriate clustering metrics (Supplementary Figs. 1 and 2, online resource). We detected a total of 11 major clusters, with negligible confounding effects by PMI, sex, age at death, *Thal* phase or *Braak & Braak* stage. Nevertheless, divergent regional atrophy patterns between PSP and CBD cases cannot completely be excluded. For cell type annotations, we gathered canonical marker genes from the literature and aggregated their gene accessibility (GA) score per cluster (Fig. 2d; Supplementary Fig. 3; Supplementary Data, T01, online resource). The GA score is derived from the sum of open chromatin loci overlapping a gene and its respective regulatory elements and can thus be understood as a proxy for gene transcription.

While neurons were represented by 5 and oligodendrocytes by 3 distinct clusters, microglia (Mic), astrocytes (Ast), and oligodendrocyte precursor cells (OPC) were each assigned to a single cluster. Neuronal subtypes were classified as (i) excitatory upper-layer neurons (Exc. ULN) with increased GA of *CBLN2, CUX2,* and *RASGRF2*, (ii) excitatory deep-layer neurons (Exc. DLN) with *RORB, FOXP2,* and *TSHZ2*, (iii) *NEFM-* and *BCL11B*-positive excitatory neurons (Exc. N. NEFM|BCL11B) as well as (iv) inhibitory neurons with either *PVALB* and *TAC1* or *SST* GA (Inh. N. SST|PVALB), and (v) inhibitory neurons with high *VIP*, *TAC3*, and *CALB2* GA. Oligodendrocytes were identified based on high GA for *MOBP* (Oli #1 MOBP) and numbered consecutively (Oli #2, Oli #3). Across all cells, Oli #1 MOBP was the most and Oli #3 the least abundant cell type (Fig. 2b, c). Because visual inspection of the *Thal* phase projection in UMAP suggested an uneven distribution across cell types, we performed a post hoc correlation analysis. This revealed only a positive association (*R* = 0.65, *p* = 0.017) between microglia and the cerebral distribution of Aβ + plaques (i.e., *Thal* phases) in our dataset (Supplementary Fig. 4, online resource), an association that has been described in the context of AD [20].

Gene ontology (GO) enrichment analyses supported cluster identities by recapitulation of known, commonly ascribed biological functions (Supplementary Fig. 5, online resource). Interestingly, microglial and astrocytic annotations were largely overlapping, hinting at an immune-regulatory role also for astrocytes in PSP/CBD. Transcription factor motif enrichment (TFME) comparisons demonstrated cluster-specific TF patterns, e.g., for Mic (SPI, RUNX2, IRF family), neurons in general (ZBTB18, RORA), and Ast (UNCX, LMX1A/B, HOXB2/3) (Supplementary Fig. 6A, online resource). Finally, cross-correlation of TFME matrices showed strong overlap within the major glial and neuronal cell types (Supplementary Fig. 6B, online resource), altogether indicating a technically and biologically consistent definition of clusters.

The frontal cortex is frequently affected by neurodegeneration and glial pathology in CBD and PSP [64]. Thus, we hypothesized that cell type proportions might appear shifted towards more glial cells in such areas and sought to calculate cell type frequencies per case (Fig. 2b). Comparing disease groups after normalization to Ctrl indicated a statistically significant decrease of neurons, OPCs, and oligodendrocytes in CBD, whereas there was only a reduction of Exc. ULN in PSP (*Welch* *t* test, Fig. 2c). However, we noted a greater cell type proportion variance in the CBD group. Because it is generally believed that qualitative immunohistochemical changes in tauopathies can be observed in both hemispheres, cases in this study were evaluated for inclusion with immunohistochemistry on the contralateral side. Thus, we cannot exclude the possibility that neurodegeneration or gliosis differed between hemispheres for certain cases. While only few reports regarding the asymmetry of immunohistochemical changes in PSP exist, this was previously described in the context of CBD [18, 64].

### Broad chromatin changes at tauopathy-associated and protein degradation-related gene loci

**Fig. 3:**
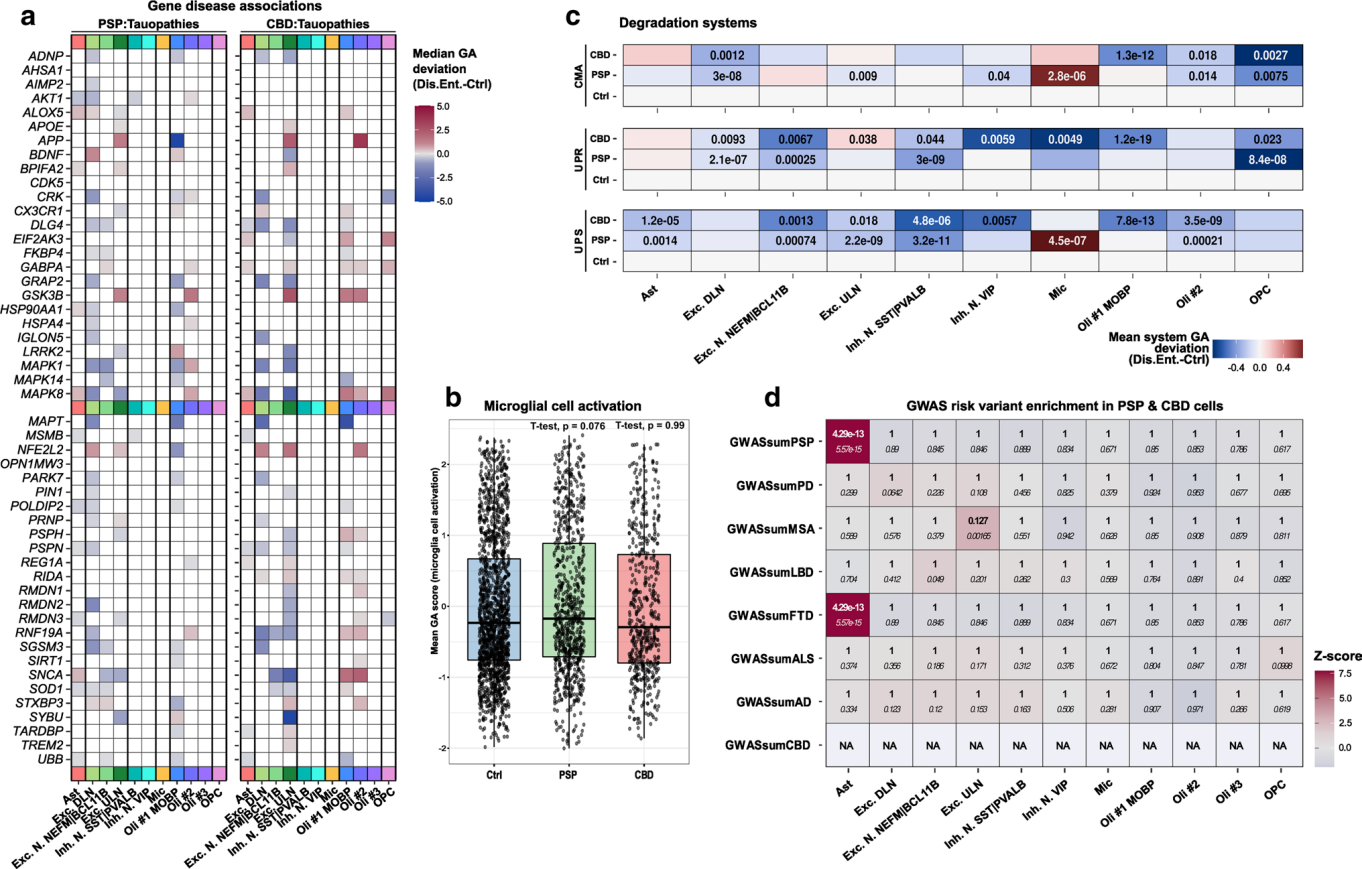
Differential accessibility analysis reveals prominent changes in tauopathy-associated genes in neurons and glia. **a** Heatmap indicating significance and magnitude of GA changes at the top50 tauopathy-associated gene loci in PSP-(left) and CBD-(right) assigned cells and their gene-cell type pairs. Color shading represents the difference from reference gene-cell type pairs in Ctrls. Only gene-cell type pairs with *p* <  = .05 are depicted (*Wilcoxon* rank-sum test, *BH*-correction). All major cell types except for microglia (and OPCs in PSP) exhibit significant GA changes in respect of these tauopathy-associated candidates. **b** Boxplots of *microglial cell activation*-associated GA patterns (AmiGO database) in microglia of the snATAC-seq dataset indicate no significant differences between compared groups. Nuclei-specific GA means of genes related to this GO term are given on the *y*-axis and compared between group entities on the *x*-axis. Single nuclei are depicted as black dots. The hinges of each box correspond to the 25th and 75th percentiles with medians drawn as black bar. The 1.5-times inter‐quartile ranges are shown as black whiskers. Two-tailed *Welch's* *t*-test, referencing the Ctrl set, *p* values as indicated. **c** Protein homeostasis-related genes across all cell types differentiated by group entity and degradation pathway show reduced system-level GA in Ast, most neuronal, and Oli populations, while Mic exhibit marked CMA and UPS inductions in PSP. Color coding shows aggregated mean scores of accessibility values at genes that were altered significantly and associated with either the CMA (top), UPR (middle), or UPS degradation systems (bottom). *P* values are given for each group vs. Ctrl comparison (two-tailed *Welch* *t* test, if *p* <  = .05). **d** Heatmap of genetic risk variant enrichment results in tauopathy cortices resolved by cell type assignments (*x*-axis) and GWAS data set (*y*-axis) highlight Ast, which exhibit strong enrichment. Color code indicates *z* scores and text inserts depict the uncorrected *p* value (*italic*) as well as the *BH*-corrected *p* values (bold, *Wilcoxon* rank-sum test). *Abs.diff.* absolute difference, *CMA* chaperon-mediated autophagy, *Dis.Ent.* disease entity, *Exc. DLN* excitatory deep-layer neurons, *Exc. ULN* excitatory upper-layer neurons, *FDR* false discovery rate, *GA* gene accessibility, *Inh. N.* inhibitory neurons, *Mic* microglia, *NA* not assessable, *Oli* oligodendrocytes, *OPC* oligodendrocytic precursor cells, *UPS* ubiquitin–proteasome-system, *UPR* unfolded-protein-response

To gain insight into disease-specific epigenetic alterations, we retrieved tauopathy-associated genes from *DisGeNET*, a database that integrates GWAS results, animal model experiments, and literature references [47]. We assessed their *z*-scored accessibilities in the disease and Ctrl groups (*Wilcoxon* rank-sum test, *Bonferroni* correction). We found altered GA scores of the top 50-ranked tauopathy-associated genes in all primary cell types except for the Mic cluster, which showed no significant changes at the selected genes in both disease entities (Fig. 3a). Due to the prominent involvement of microglia in AD [42, 45, 46]—another tauopathy—this finding was unexpected. Nevertheless, we could additionally show that there was no GA difference between groups when aggregating scores for all genes associated with the GO term “microglial activation”, supporting the notion that this phenomenon is not driven by epigenetic mechanisms in CBD or PSP (Fig. 3b).

Of note, *MAPT* showed reduced GA scores in one oligodendroglial and one neuronal cluster (Oli #1 MOBP and Exc. DLN) for both diseases, with a more pronounced reduction in CBD and unchanged conditions in Ast. The most significant positive GA change was observed in *NFE2L2* (Nuclear factor erythroid 2-related factor 2) in excitatory neurons, with a stronger gain in CBD compared to PSP. While in PSP, most significant hits were attributed to Exc. DLN, Exc. ULN were affected by the most extensive changes in CBD (Fig. 3a, Supplementary Fig. 7A&B, online resource).

Many tauopathy-related genes, including MAP-kinases and lysosomal enzymes, are associated with protein homeostasis [29, 66]. Thus, we aggregated GA scores for three molecular entities known to be involved in protein degradation (Fig. 3c): the ubiquitin proteasome system (UPS), the unfolded protein response (UPR), and chaperone-mediated autophagy (CMA). All three pathways were downregulated in oligodendrocytic populations in CBD with primarily downregulated CMA and UPS in PSP-derived Oli #2. We observed concordant UPS reductions in CBD and PSP Ast (*p* < 0.005) with simultaneously reduced microglial UPR (*p* < 0.001). Contrarily, PSP Mic exhibited a marked activation of both CMA and UPS (*p* < 0.001). With respect to the neuronal populations, downregulation of all three systems was apparent. However, in CBD-derived Exc. ULN, the UPR system was induced, reflecting neuronal heterogeneity also in terms of degradative pathways.

On the DNA sequence level, GWAS risk variants associated with neurodegenerative diseases might be linked to ATAC-seq peaks, which are several hundred bp long regions of open chromatin, aggregated by cluster. Assuming that this relation would likewise affect certain cell types more than others, we compiled GWAS summary statistics for tauopathies and related neurodegenerative syndromes, namely PSP, CBD, AD, Frontotemporal Dementia (FTD), Parkinson Disease (PD), Multiple System Atrophy (MSA), Lewy Body Dementia (LBD), and Amyotrophic Lateral Sclerosis (ALS). We then inferred cell type-associated risk variants in our dataset (as pooled tauopathy nuclei) segregated by disease-specific peaks (Fig. 3d, see Methods). This analysis showed pairs of high z scores and highly significant enrichment for FTD- and PSP-associated risk variants in astrocyte-specific peaks. We were unable to calculate cell type enrichments for CBD risk variants due to the sparsity of CBD GWAS (the significance-filtered CBD GWAS list consisted of only 6 SNPs). Strikingly, we did not observe GWAS enrichments for any of the above neurodegenerative diseases in microglia-specific peaks—not even AD, for which other studies have clearly attributed a large genetic risk proportion to microglia [42, 45, 46], suggesting that PSP- and CBD-specific microglial peaks are distinct from AD-specific ones.

Collectively, this investigation demonstrates that genetic risk variants previously associated with the clinical spectrum of primary tauopathies (i.e., PSP and FTD) are tightly linked to *astrocytic* chromatin accessibility profiles in the brains of PSP and CBD patients. Nonetheless, systematic cell type and pathway annotations also point to inherent differences between these diseases.

### Tracking epigenetic transition states of tauopathy astrocytes supports a context of neuroinflammation

**Fig. 4:**
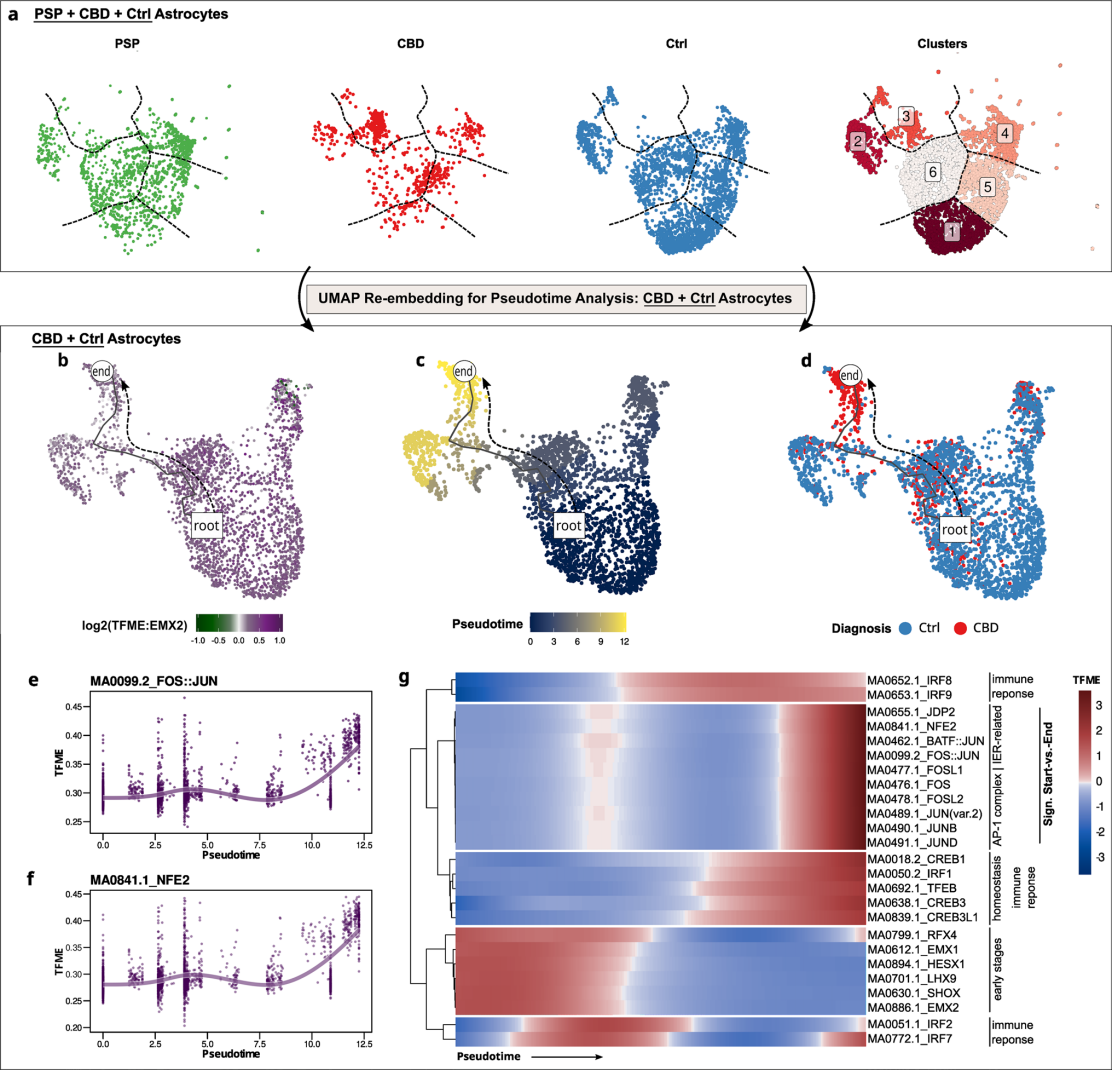
CBD astrocytes acquire an epigenetic state of stress response and neuroinflammation. **a** All *PSP-, CBD-, and Ctrl*-derived astrocytes re-embedded in UMAP, stratified by group entity (first, second, third panel), and depicted after k-means clustering in a merged UMAP (fourth panel). One cluster (#3) is specific for CBD astrocytes. Color code indicates group entity or cluster assignments in the first three or the fourth panel, respectively. Dashed lines delineate cluster borders and are transferred to the group-wise depictions. **b**–**d** Exclusively *CBD- and Ctrl*-derived astrocytes re-embedded in UMAP. A pseudotime trajectory leads from a non-specific Ast pool towards a CBD-enriched population. Color code indicates EMX2 TFME (**b**), pseudotime (**c**, dimensionless), or group entity (**d**). The black line indicates the pseudotemporal trajectory from the *root* towards the *end* cell. **e, f** Generative additive model non-linear fits of TFME values over pseudotime of the FOS-JUN (**e**) or NFE2 (**f**) motifs indicate parallel increments during the astrocytic transition towards a CBD-state. **g** Pseudotime heatmap displaying the TFME values of significantly altered TFMs in the start-vs.-end comparison (*Wald*-testing, *BH*-adjusted *p* < .05), as well as markers of early astrocytic development or immune regulation. Biological pathway associations are given on the right. TFME of astrocytic early-stage TFs is gradually decreasing, while immunologically relevant and AP-1 complex-related TFs gain in motif enrichment. *IER* immediate-early response, *TFME* transcription factor motif enrichment, *Sign* significant

Integrating the previous findings with the knowledge that the astrocytic phenotype constitutes a major neuropathological feature to distinguish CBD from PSP impelled us to focus our downstream investigations on the Ast cluster. Thus, we subjected all 4425 astrocyte-derived nuclei from both tauopathies and Ctrl to subclustering and annotation procedures. One astrocytic subcluster exclusively consisted of tauopathy-derived nuclei (mainly CBD) and exhibited higher accessibility at genes involved in ‘stimulus detection’ or ‘signal transduction’ (Fig. 4a, Supplementary Fig. 8A, online resource). A triangular disease-wise comparison of TF motif enrichment (TFME) values (Supplementary Data, T02-T03, online resource, *Wilcoxon* rank-sum test, *BH*-corrected) revealed that the most significant candidates were those TFs associated with immunological terms (Supplementary Fig. 9A-E, online resource), while TF deviations were stronger in CBD than in PSP astrocytes (Supplementary Fig. 9F, online resource).

To gather insight into *how* astrocytes derived from tauopathy brains might evolve from a physiological towards a diseased state, we hypothesized this transition to be a continuous process paralleled by changes in open chromatin and mirrored by differential accessibilities for specific TFs along a shared time constant. Hence, we sought to understand TFME dynamics with pseudotemporal models, though in separate UMAP embeddings for each disease entity.

We reasoned that high accessibility for the TF EMX2, which is specific for and active in early differentiating astrocytes, would be a suitable starting point for an assumed astrocytic transition path [58] (Fig. 4b–d). For CBD, we obtained a trajectory that terminated in a population of CBD-derived astrocytes, while only few of them were embedded in proximity to Ctrl cells in the EMX2_HIGH_ population. This is consistent with the presence of unaffected astrocytes in the tauopathy brain. In PSP brains, however, no disease-defined astrocytic subcluster was evident (Supplementary Fig. 8B, online resource).

The pseudotime inference itself does not allow for statistical evaluation of single-cell feature values over pseudotemporal trajectories. Thus, a framework called *tradeSeq* [3] was employed to test for TFME changes along the astrocytic transition axis. In a first step, generative additive models were fit to the feature distributions as a function of pseudotime. Subsequently, *Wald*-statistic-based hypothesis testing allowed to discern TFMs whose respective TFME values were either associated with the trajectory course or differed significantly from starting to terminating points.

This analysis showed a diverging pattern: The immediate early response (IER)-related FOS and JUN family members as well as their co-transcriptional factors NFE2, JDP2, and MAF, known to co-act as the AP-1 complex in the regulation of cell growth, differentiation, inflammation, and apoptosis [50, 61] (Fig. 4e–g, Supplementary Fig. 10A, Supplementary Fig. 11A&B, online resource), showed significantly higher and highly correlated TFME values (*Wald*-statistic start-vs.-end comparison, *BH*-corrected p value < 0.05). In contrast, TFs related to the early stages of astrocyte differentiation (LHX9, SHOX, RFX4, HESX1, and EMX2), showed a gradual loss of their enrichment (Fig. 4g, Supplementary Fig. 10A, Supplementary Fig. 11C–E, online resource). Focusing on pseudotime-aligned GA changes, protein homeostasis-related genes (e.g., *APOE, HSPB8, LRP1*) were upregulated, while many synaptic candidates (e.g., *SNCA*, *BDNF, NRXN3*) gradually decreased in their GA in CBD astrocytes (Supplementary Fig. 10B, online resource).

This analysis demonstrated that in CBD astrocytes, chromatin accessibility of TFs implicated in early astroglial development appears to be reduced in favor of a neuroinflammatory response.

### Reconstructing disease-specific representations of astrocytic TF networks and a phenotype-associated regulon activity profile

**Fig. 5:**
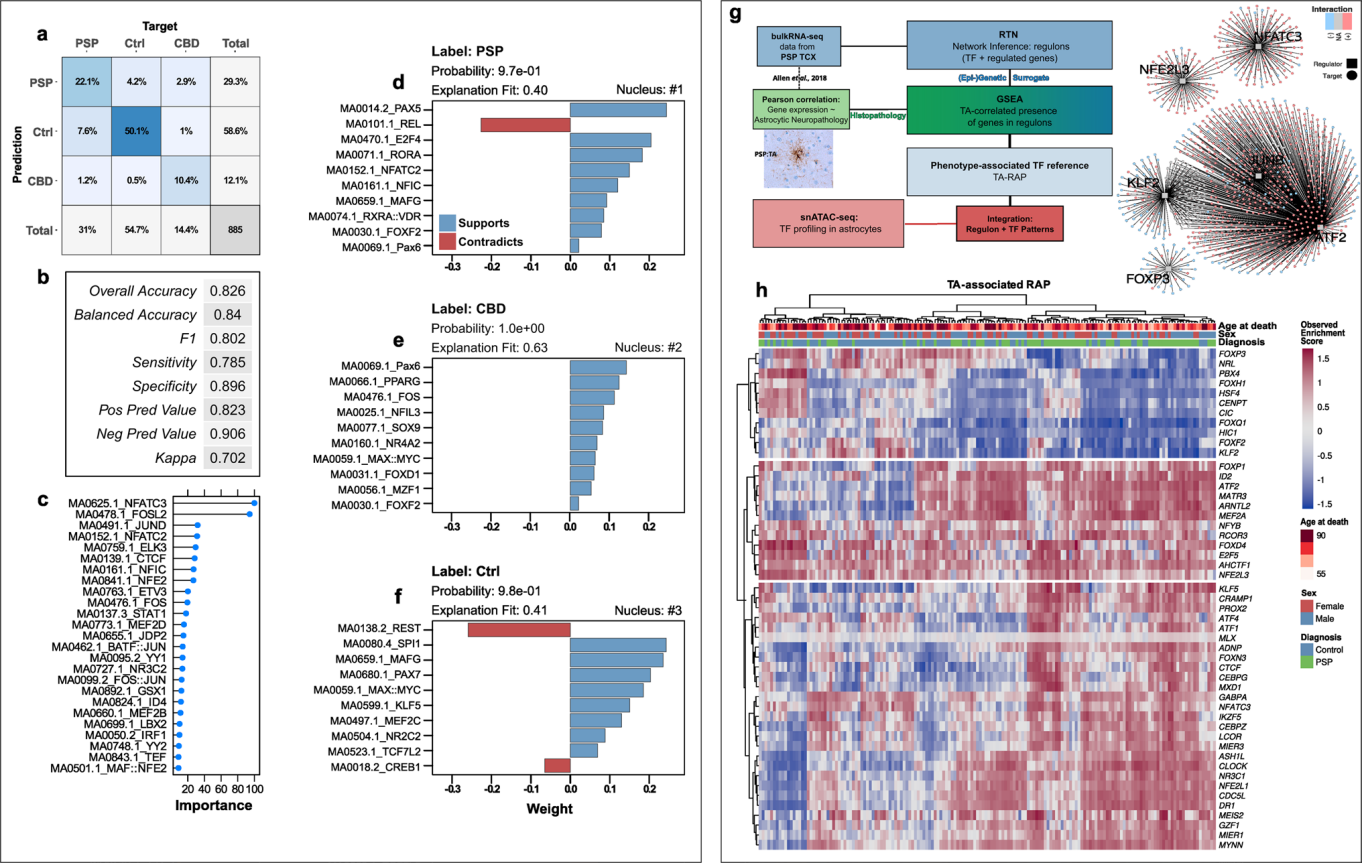
TF networks associated with the astrocytic tauopathy state and regulatory correlates of tufted astrocytes. **a** Confusion matrix displaying the intersections of the XGB model’s predictions (rows) and the actual labels (columns). Each square contains the percentual proportion of test set samples (= 20% nuclei) with the assigned prediction–label relation. The sums of each row or column are depicted in the rightmost column or bottom row, respectively. The total sample number (i.e., nuclei of the 20% test set-split) is shown in the bottom right corner. **b** Evaluation parameters of classification performance of the trained XGB model on the 20% test set-split. Overall, more than 82% of predictions were correct (overall accuracy) and the model performs "substantially" with a *Cohen* kappa of 70.2%. **c** Overall feature importance values of the top 25 TFMs included in training the XGB model to correctly classify an astrocyte TF representation in general. The x-axis differentiates the feature importance (%) as reported by *caret’s* varImp function. Immediate-early response candidates (NFAT2/3) and major AP-1 constituents (FOSL2, JUND) were among the most important TFs. **d–f** *Lime* feature importance bar diagrams of the most certainly correctly classified barcodes of each group entity. The bar direction and bar color indicate the feature weights (~ importance) assigned to the TFM, which are given as *y*-axis breaks. Feature weight was assigned to specific TFME value ranges. Each panel is complemented by the group entity label, the model's calculated probability, and the explanatory model’s fit value. **g** Bioinformatical concept of the RTN analytical approach to link a neuropathological phenotype to TF information. A regulon network was inferred from published bulkRNA-seq data in PSP TCX and filtered subsequently for those regulons that showed phenotype association (i.e., gene set enrichment of DEGs with histopathological TA grading in PSP cortices). Thereby, a TA-associated regulon activity profile was deduced, which was employed as TF reference in an integration part with snATAC-seq data-derived astrocytic TF activity patterns. Ultimately, this approach served to refine pTau-inclusion pathology-associated astrocytic PSP/CBD signatures. On the right, a set of TA-linked regulons illustrates the modularity of TF-gene-interactions (color code), the inter-modular connectivity suggesting co-regulation exerted by regulators on common genes, and the presumed presence of distinct groups of TFs. **h** Activity heatmap of those regulons that are enriched with TA grading in PSP TCX and whose regulon activity is significantly different between PSP and Ctrl TCX samples (*p* < .05, *BH*-corrected). Regulons in the upper part correlate negatively, those in the lower part correlate positively with TAs in PSP cortices. Every column corresponds to a single TCX sample and every row to a gene while color shade indicates the extent of regulon activity change. Rows and columns were clustered hierarchically (*Euclidean* distance, *Ward-D2* method) and results indicated as dendrograms. The colored overlay informs about the age at death, sex, and definitive neuropathological diagnosis. Gene names comply with the *Ensembl* IDs. *DEG* differentially expressed gene, *GSEA* gene set enrichment analysis, *Neg Pred Value* negative predictive value, *Pos Pred Value* positive predictive value, *RTN* Reconstruction of Transcriptional Networks, *TA* tufted astrocyte, *TCX* temporal cortex, *RAP* regulon activity profile, *XGB* extreme gradient boosting tree

We wondered whether we could leverage TFME information in a more unbiased way to decipher signatures delineating PSP and CBD brains specifically from a non-diseased condition. Thus, we modeled disease-specific TF patterns by exploiting the discriminatory power of a machine-learning algorithm in a supervised classification task. Concretely, an extreme gradient boosting tree algorithm was trained on the cell-specific TFME of an 80%-split of the astrocyte subset, including data from PSP, CBD, and Ctrl astrocytes (*n* = 3540). Assessing the model’s performance with unseen nuclei of the remaining 20% of the astrocyte subset (*n* = 885, Fig. 5a, b) yielded reliable classification regarding accuracy (overall 82.6%, balanced 84.0%), positive predictive value (82.3%), and negative predictive value (90.6%). Furthermore, when dividing by the a priori expected likelihood of predicting the correct group entity, the model’s predictive ability can be considered ‘substantial’ with a *Cohen kappa* of 70.2% [10]. The differentiated prediction performance was most accurate for CBD vs. Ctrl tasks, followed by PSP vs. CBD, and PSP vs. Ctrl distinctions (Fig. 5a, Supplementary Fig. 12A, online resource).

In a subsequent step, overall feature importance assessment highlighted IER- and cellular immunity-related TFs (FOS, JUN, NFATC2/3, STAT1) as most useful in predicting the tauopathy or Ctrl state (Fig. 5c). Since this did not sufficiently inform about *disease-specific* importance, we used local interpretable model-agnostic explanations (*Lime*) [52] to understand the model’s decision-making process in more detail. For a given astrocytic nucleus, *Lime* evaluated the contributions of TFME ranges to support or contradict the respective disease label (depicted as bar length in Fig. 5d–f). Consequently, we selected the top 10 candidates ranked by their feature weight in all test set astrocytes, which were assumed to be most helpful in predicting astrocytic identity of either PSP, CBD, or Ctrl origin specifically. Interestingly, the ‘importance’ metric was not only determined by the TFME deviations with the highest changes, but also by the discriminatory power of subtle TFME changes across the compared groups (Supplementary Fig. 12B, online resource).

The previous analyses highlighted changes in astrocyte TF dynamics without any respect to the underlying neuropathological phenotype. To link this information (i.e., pTau inclusions in astrocytes) with TF activities, we used the *Reconstruction of Transcriptional Regulatory Networks* approach [9] (Fig. 5g). Enabled by the availability of bulk gene expression data and correlations thereof with tufted astrocyte quantitation data (i.e., adjusted semiquantitative TA density) from the temporal cortex (TCX) of 176 PSP patients [1], we assessed genetic regulators and their target genes as so-called *regulon units* (Fig. 5g).

Utilizing these neuropathological-transcriptomic correlations, we assigned TA-related values to the given regulons (two-tailed GSEA, differential enrichment score, Fig. 5h), which we termed tufted astrocyte-associated regulon activity profile (TA-RAP). Unsupervised clustering of regulons indicated sets with lower (top branch), mixed (mid branch), and increased (bottom branch) activity in PSP cases when compared to Ctrls. Again, IER-related and immunologically relevant TF transcripts such as *JUNB, NFATC2, NFE2L3,* or *IKZF5* were present among the set of activity enhanced regulons, while those related to developmental processes exhibited lower scores (e.g., *FOXF2*, *NRL*). Genes assigned to the TA-associated regulons shown in Fig. 5h were also enriched in biological pathways related to autophagy, peroxisomes, and (de-)ubiquitination (Supplementary Fig. 13, online resource).

These analyses highlight the importance of TF networks related to the IER or neuroinflammation in PSP and CBD astrocytes alike. Furthermore, external bulk transcriptomic data supports this notion independently from our snATAC-seq data for PSP. Nevertheless, an unbiased machine learning model also predicted discriminatory features between the two disease groups.

### Definition of tauopathy signatures in astrocytes of PSP and CBD brains

**Fig. 6:**
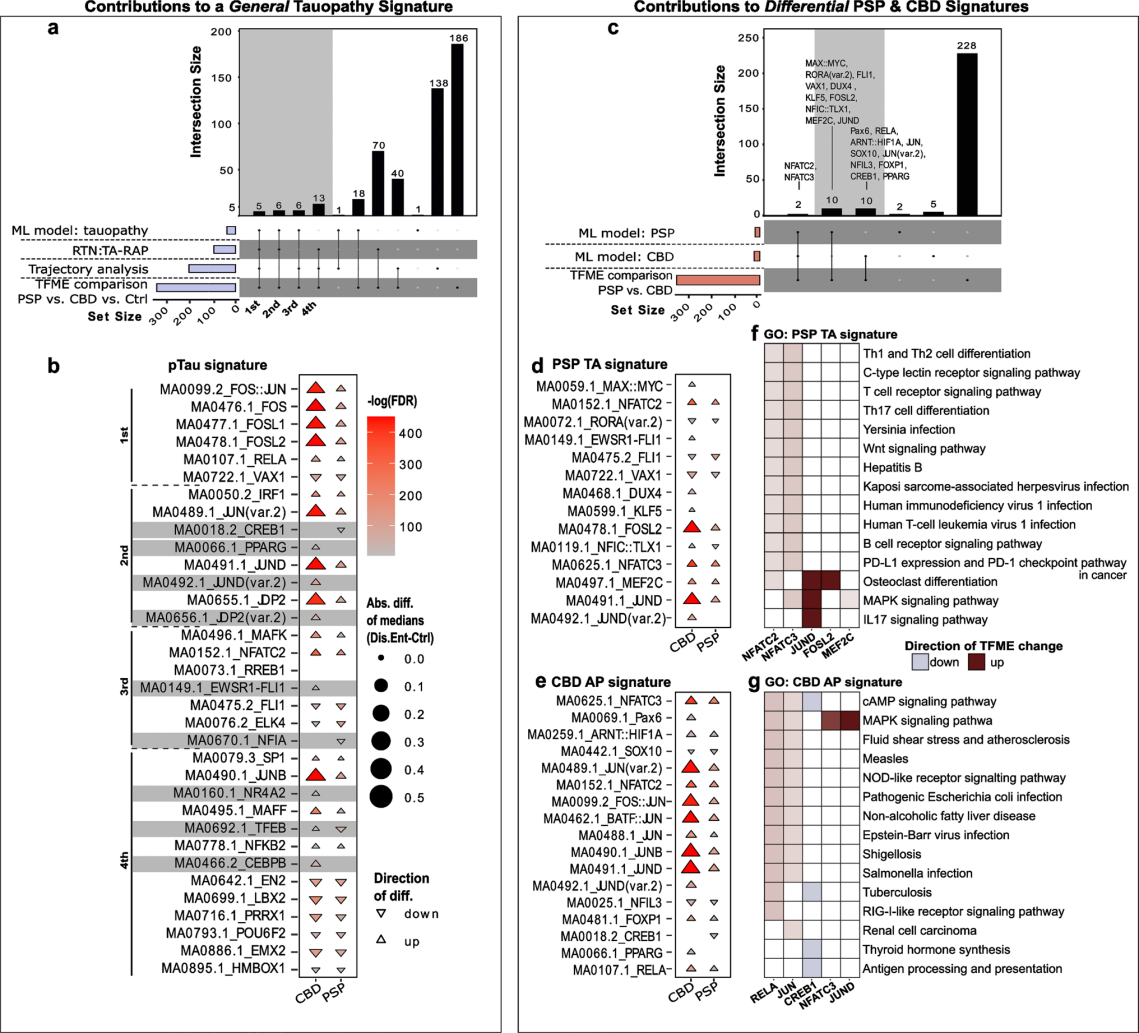
A concept of astrocytic tauopathy signatures. **a** Upset plot illustrating TFs useful in distinguishing PSP/CBD from Ctrl astrocytes that resulted from (i) interpreting the XGB classification model (‘ML model: tauopathy’), (ii) the TA-related regulon activity profile in the bulkRNA-seq data set (‘RTN: TA-RAP’), (iii) the pseudotime trajectory analysis in the snATAC-seq data set (‘Trajectory analysis’), and (iv) group-wise TFME comparisons in the snATAC-seq data set (‘TFME’ comparison’). Set sizes are indicated as blue bars, while the intersection logic is shown as vertical lines and dots. Column heights depict the extent of intersection for the given sets. The first four intersections were assigned a hierarchy of importance in defining the primary tauopathy context. **b** Triangle plot indicating significance, absolute extent, and direction of TFME changes in pTau signature TFs in tauopathy-assigned astrocytes. The triangle tips point towards the direction of change while the size represents the absolute difference from the TFME reference in Ctrls. Fill shading displays the negative decadic logarithm of the *BH*-corrected p values from pair-wise *Wilcoxon* rank-sum tests. Empty coordinates inform about non-significant comparisons. Gray underlay informs about candidates with diverging TFME when collating PSP and CBD. **c** Upset plot to identify TFs useful in differentiating CBD from PSP astrocytes. The single sets resulted (i) from the most important TFs for PSP or CBD prediction according to the XGB model explainer (‘ML model: PSP’, ‘ML model: CBD’) and (ii) from pairwise statistical TFME comparisons between PSP and CBD astrocytes (‘TFME comparisons’). The general plot structure equates to A. **d, e** Triangle plot indicating significance, absolute extent, and direction of TFME changes in PSP (**d**), and CBD (**e**) signature TFs in tauopathy-assigned astrocytes. The general plot structure equates to B. **f**, **g** Heatmaps of the GO enrichment of the PSP (**f**) and CBD TF signatures (**g**). The top 15 terms according to MF, BP, and CC enrichment scores as well as only those TFs that share at least one of these terms are depicted. Color code indicates the direction and strength of enrichment or depletion compared to Ctrl astrocytes. MAPK signaling, immunological and infectious disease terms are enriched. *Abs.diff.* absolute difference, *AP* astrocytic plaque, *CMA* chaperon-mediated autophagy, *DAR* differentially accessible region, *Dis.Ent.* disease entity, *EC* extracellular, *Exc. DLN* excitatory deep-layer neurons, *Exc. ULN* excitatory upper-layer neurons, *FDR* false discovery rate, *GO* gene ontology, *Mic* microglia, *ML* machine learning, *Oli* oligodendrocytes, *OPC* oligodendrocytic precursor cells, *TA-RAP* tufted astrocyte-associated regulon activity profile, *TF(ME)* transcription factor (motif enrichment), *UPS* ubiquitin–proteasome-system, *UPR* unfolded-protein-response

The primary objective of this study was to define epigenetic signatures for PSP and CBD based on TF networks. Hence, we finally integrated the results of the four disjoint analysis branches into consensus lists (Fig. 6, for concept see also Fig. 1). The first set contained the names of all TFs that showed significant TFME changes in disease-wise statistical comparisons within the entire astrocyte population. The second set consisted of TFs whose accessibility profiles aligned tightly with the pseudotime trajectory. ML model explanations indicated the TF candidates contributing to the third set. Lastly, those TFs resulting from TA-RAP extraction in the external PSP dataset represented the members of the fourth set. To determine the consensus of candidates nominated by these different approaches, we used upset plots. We first describe a common PSP and CBD “pTau” signature that likely represents the regulatory state of pTau-positive astrocytes (Fig. 6a, b). Through hierarchical ordering by (i) the number of branches that overlap and (ii) the extent of overlap in terms of TF candidate counts, 4 ranks of confidence were assigned to TF sets with >  = 3 set intersections. Guiding in discerning a tauopathy (PSP and/or CBD) from a Ctrl frontal cortex was the proposed astrocytic tauopathy signature depicted in Fig. 6b, with increased accessibilities for TF binding sites related to the IER such as JUN, FOS, and its ligands FOSL1 and FOSL2.

When directly comparing CBD and PSP TF binding site accessibilities, it became apparent that their changes followed almost exclusively the same direction, with the exception of TFEB and NFIC::TLX1, which showed lower accessibilities in PSP but greater ones in CBD. Meanwhile, we observed relatively strong differences in the extent of accessibility alterations. Thus, to discriminate the two tauopathies, we hereby propose JUN(B,D)_HIGH_, FOS(L1,L2)_HIGH_ as well as the involvement of DUX4, KLF5, MAX::MYC, PAX6, and PPARG to support the identity of CBD-originated astrocytes (Fig. 6c–e). PSP astrocytes exhibited a signature with additionally decreased TFEB and CREB1 accessibilities. Interestingly, subsequent pathway enrichment analyses of the PSP and CBD signatures incorporating the logic of TFME changes suggested MAPK-dependent signaling and infectious agent defense terms to play important biological roles in primary tauopathy astrocytes (Fig. 6f, g). In addition, the RELA and JUN TFs link back to protein homeostasis pathways (i.e., ‘ubiquitin protein ligase binding’), which is reflected in the gradual accessibility increments of tauopathy- and degradation system-associated genes (Supplementary Fig. 14, online resource).

Collectively, the integration of a variety of analytic approaches resulted in the identification of an astrocytic pTau signature that is strongly reminiscent of an immediate-early response. Notwithstanding, distinct molecular states of astrocytes in PSP and CBD were identified as well.

Since a conceivable confounder—case CBD3—might have introduced a sampling bias through its relatively high proportion of astrocytes, we performed a separate re-analysis without that case. Even with this approach, the major results of our study remain unaffected; however, we detected a significant increase in relative astrocyte frequencies (likely due to the lack of the outlier sample CBD3) and a more comprehensive list of TFs supporting a pTau phenotype (Supplementary Fig. 15–18, online resource).

### Altered JUNB and TFEB activity influences target protein expression

Finally, we wondered whether the altered TF regulatory networks in tauopathies would manifest themselves in altered protein levels that could be observed by immunohistochemistry in post-mortem brains. We therefore identified target genes of the prominent TF candidates JUN(B) and TFEB, both of which are master regulators of essential homeostatic pathways.

**Fig. 7:**
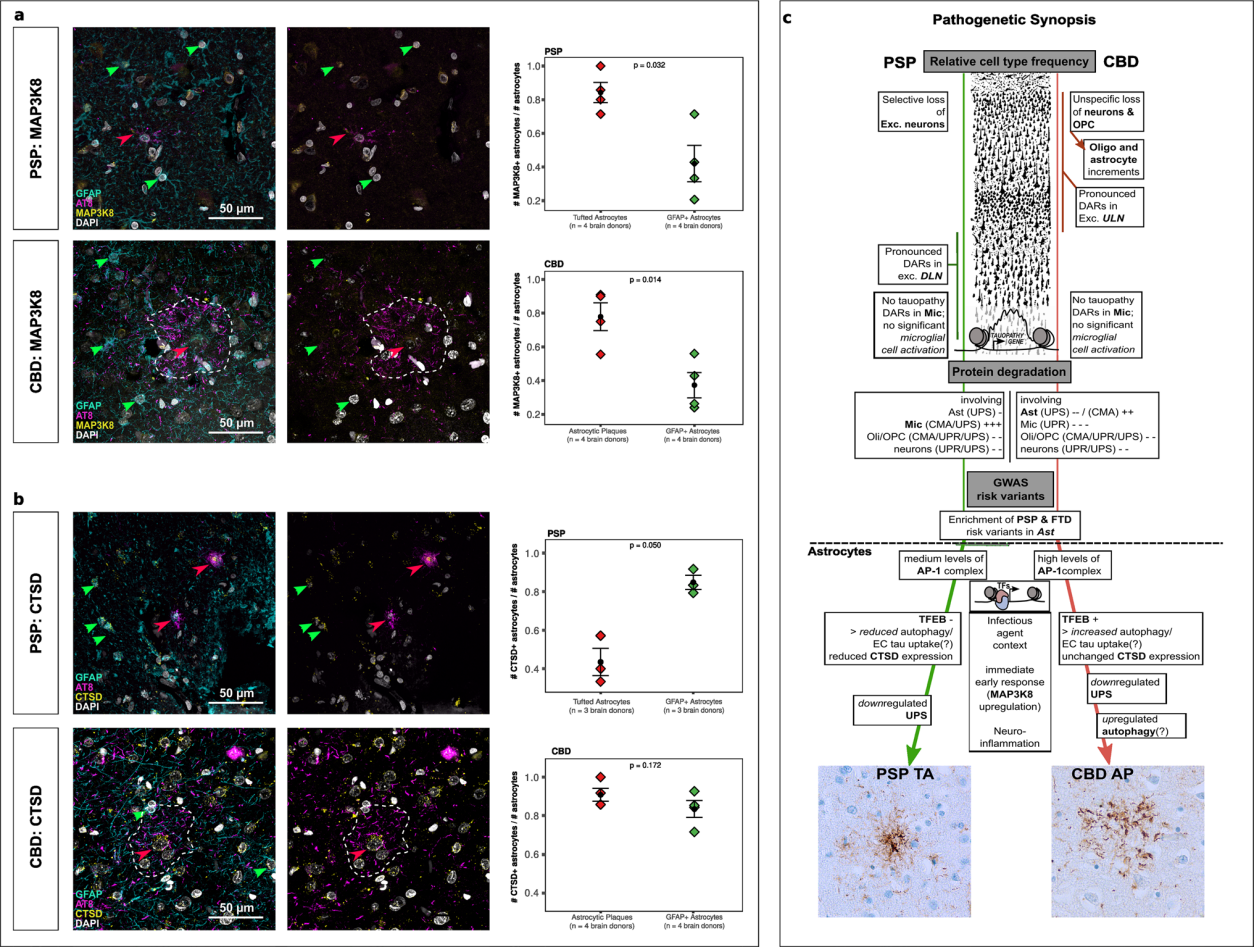
TF target gene validation and synopsis of pathogenesis. **a** Immunofluorescent staining analysis showing 4-channels merge (GFAP, AT8, MAP3K8, DAPI) and 3-channels merge (AT8, MAP3K8, DAPI) of PSP (upper row) and CBD (lower row) MFG. Arrowheads mark GFAP + (green) and AT8 + (red) astrocytes. Boxplots depicting fractions of GFAP + MAP3K8 + astrocytes over GFAP + astrocytes compared with the fractions of AT8 + MAP3K8 + astrocytes over AT8 + astrocytes. Statistics were calculated using a two-tailed *paired* t-test with p values as indicated. **b** Immunofluorescent staining analysis showing 4-channels merge (GFAP, AT8, CTSD, DAPI) and 3-channels merge (AT8, CTSD, DAPI) of PSP (upper row) and CBD (lower row) MFG. Arrowheads mark GFAP + (green) and AT8 + (red) astrocytes. Boxplots depicting fractions of GFAP + CTSD + astrocytes over GFAP + astrocytes compared with the fractions of AT8 + CTSD + astrocytes over AT8 + astrocytes. Plot structure equal to a. **c** Concept of epigenetic contribution to the pathogenesis in the primary 4R tauopathies PSP and CBD. The upper half summarizes global findings of this study, while the lower half focuses on changes assigned to astrocytes. Differences in neuronal cell loss were observed and mirrored by prominent DAR-patterns in different neuronal subclusters. Protein degradation was induced in Mic in PSP, while Ast served this role in CBD. PSP and FTD-associated risk variants were exclusively enriched in Ast. Focusing on the latter glia type, disease-specific molecular patterns comprising regulators of the immediate early response, autophagy, and UPS delineate differential pathogenetic signatures. The histological illustration of the neocortex was modified from https://commons.wikimedia.org/wiki/File:Cajal_cortex_drawings.png. *AP* astrocytic plaque, *CMA* chaperon-mediated autophagy, *CTSD* Cathepsin D, *DAR* differentially accessible region, *Dis.Ent.* disease entity, *EC* extracellular, *Exc. DLN* excitatory deep-layer neurons, *Exc. ULN* excitatory upper-layer neurons, *FDR* false discovery rate, *GO* gene ontology, *MAP3K8* Mitogen-activated protein 3 kinase 8, *Mic* microglia, *ML* machine learning, *Oli* oligodendrocytes, *OPC* oligodendrocytic precursor cells, *TA-RAP* tufted astrocyte-associated regulon activity profile, *TF(ME)* transcription factor (motif enrichment), *UPS* ubiquitin–proteasome-system, *UPR* unfolded-protein-response

To this end, we selected the top gene candidates resulting from correlating JUN(B)/TFEB TFME and GA, and which overlapped with *Cicero* co-accessibility links on the genomic level (see Methods). This approach highlighted *MAP3K8* as JUN(B)- and *CTSD* as TFEB-targeted genes (Supplementary Fig. 19, online resource). We performed immunofluorescence labeling of those candidates’ gene products in postmortem PSP and CBD samples (i.e., MFG) to validate JUNB and TFEB network dysregulation though their predicted target genes (Fig. 7a, b).

The mitogen-activated protein kinase *MAP3K8* (also known as TPL2) can transduce pro-inflammatory stimuli (i.e., IL17-receptor signaling in astrocytes) through the JUN(B)/FOS TF complex, which itself can enhance *MAP3K8* and interleukin expression [43, 63, 65]. We observed a significantly higher rate of pTau-positive astrocytes (TAs and APs) with MAP3K8 expression compared to GFAP positives in both tauopathies (PSP: 0.83 vs. 0.42, *n*_cells_ = (118; 23), *n*_cases_ = 4; CBD: 0.79 vs. 0.37, *n* = (83; 34), *n*_cases_ = 4) (Fig. 7a). In contrast, when investigating the expression of Cathepsin D, a lysosomal hydrolase and marker of lysosomal degradation [48], we found an inverse association with CTSD + TAs (in PSP), while the fraction of CTSD + APs (in CBD) was not significantly higher than their GFAP + counterparts (PSP 0.44 vs. 0.84, *n*_cells_ = (65; 18), *n*_cases_ = 3; CBD 0.91 vs. 0.85, *n*_cells_ = (87; 36), *n*_cases_ = 4) (Fig. 7b). Notably, protein expression was also visible in neuronal somata and other non-astrocytic cells. Together, we conclude that tauopathy astrocytes show signs of a JUN(B)-mediated pro-inflammatory state, while TAs in PSP additionally display a TFEB-mediated downregulation of lysosomal degradation.

Collectively, our findings are summarized in a flow chart (Fig. 7c) that can be used as an informative reference for epigenetic signatures that play a role in PSP and CBD.

## Discussion

In this study, we applied an integrative systems biology approach to capture single-nucleus chromatin accessibilities in PSP and CBD frontal cortices. By combining latent characteristics of TF information and external transcriptome data, we shed light on the regulatory identity of pTau-affected astrocytes in these neurodegenerative diseases. Previous studies in bulks of brain cells repeatedly highlighted tauopathy-associated genes, or genes harboring genetic risk variants in their proximity [1, 27, 34]. However, cell type-resolved data sets have only been published for AD, where a role for microglia in its pathogenesis is now relatively well established. To our surprise, we did neither find microglia-associated accessibility changes, nor did we see an association of neurodegeneration-specific genetic risk loci with microglial specific peaks in the PSP or CBD datasets. In contrast, our data highlight astrocyte-relevant epigenetic alterations (Fig. 7c) [6, 55, 62]. Responses to pathological stimuli associated with the occurrence of pTau are likely related to astrocytic subpopulations, as delineated by differential use of TF binding sites in our study [15, 17, 30]. Nevertheless, we cannot exclude the possibility that accessibility changes in astrocytes are secondary in nature, without any direct effect of pTau on the compaction of chromatin, even though this has been described before in Tau transgenic *Drosophila* [19].

We propose that such TF signatures are meaningful (i) descriptively as indicators of disease entity, and (ii) biologically as the representation of a pathogenetically relevant gene expression program. The diagnostic value of this analysis might aid future TF-based studies with latent or missing phenotype data in a priori trait mapping. Together with the proposed TF signature-based disease description, the underlying biology might even elicit candidates worth targeting pharmacologically. For example, one concept in AD aims at promoting lysosomal biogenesis and degradative enzyme expression. Astrocyte-specific conditional knock-out experiments in mouse models and neuronal cultures previously suggested to target TFEB, a transcriptional inductor of autophagic lysosomal degradation [5, 39]. This lysosomal master regulator significantly reduced the load of neuronal pTau and shifted extracellular Tau fibrils into astrocytes for lysosomal degradation. In our data, increased TFME values of TFEB in CBD astrocytes were paired with autophagy activation without significant rise in CTSD protein levels (in APs), while reduced TFEB activity and CTSD expression (in TAs) was evident in PSP astrocytes. This adds the notion of deficient activity in the latter and potentially defective or inefficient lysosomal pathways in the former disease context. TFEB could thereby activate antioxidative and autophagy processes in synergy with NFE2 [32], a TF which featured increased motif enrichment in CBD astrocytes and whose deficiency is associated with exacerbation of Tau and amyloid pathology [53]. In turn, NFE2 family members are dependent on co-regulators and the AP-1 complex, a regulatory machinery implicated in neuroinflammation, apoptosis, gliotic remodeling, and axonal repair [50, 61]. This heterodimeric regulator complex exhibits cell type-specific composition and response profiles but has merely been in the focus of tauopathy research.

Immunohistochemical analysis in *postmortem* brain tissue of individuals with the very rare 3R tauopathy Pick's disease revealed colocalization of AP-1 components such as FOS, JUN, and MYC with the disease-defining intraneuronal pTau deposits (*Pick* bodies) and neuronal cytoplasms [44]. PSP and CBD astrocytes seem to acquire a pathological state of reactivity, cellular stress, and potentially apoptosis upon AP-1 activation (Fig. 6h). To our knowledge, an IER signature, comprising the AP-1 subunits, NFKB2, and NR4A2, has not been described in 4R tauopathies.

AP-1 itself might be induced by MAP kinases, as suggested by the simultaneous increase in the GA of *MAPK8/*JNK1 and protein levels of *MAP3K8*/TPL-2 in (pTau +) astrocytes of both tauopathies. In parallel, activated *MAPK8*/JNK1 in astrocytes might perpetuate Tau hyperphosphorylation, thereby contributing to a persistent cellular stress response [19, 51]. In neurons, the reduced GA of *MAPKs* (isoforms 1,8,14) and increased GA of their downstream target *GSK3B* suggest a potential target point by means of therapeutic kinase inhibition. Unfortunately, previous clinical trials with unspecific kinase inhibitors (valproic acid, lithium) and the selective GSK3B-inhibitor tideglusib failed to achieve considerable clinical improvements in AD or PSP patients [23, 36, 59]. Complicating the kinase modulation concept, increased GA of *MAPK8/*JNK1 was evident in astrocytes and oligodendrocytes alike, again highlighting the importance of cell type-resolved analyses.

Intriguingly, the GWAS risk variant enrichment analysis revealed enrichments only in astrocyte-specific chromatin peaks. Microglial-driven alterations in genes participating in protein homeostasis, which are commonly believed to be a hallmark of AD [13, 22, 56], seemed to be confined to PSP in our analysis (Fig. 3b), consistent with the pronounced microglial transcriptional networks observed by Allen et al*.* [1]. The lack of risk variant enrichment as well as the significant GA increase in genes associated with UPS and CMA suggest that microglial activation in PSP is rather unlikely determined by sequence alterations as defined in GWAS. Unlike other tauopathies such as AD or Pick's Disease, the pTau-laden astrocytic phenotype is considered a hallmark of PSP and CBD, where loss of synapse support and concurrent inflammation can exert deleterious effects [4, 6, 37]. Our results therefore suggest that future experiments should prioritize the investigation of astrocytes over microglia in PSP and CBD.

In summary, comparing these two 4R tauopathies from an epigenetic perspective demonstrates broad overlap in terms of disease-associated regulatory processes. Nevertheless, our analysis also suggests distinct pathogenetic properties, particularly with regard to the phenotype of astrocytes. The proposed tauopathy signatures should be contextualized with different data sets and more diverse disease populations to define their specificity as well as overlapping features with related disease entities. Future research might also benefit from integrating our data in multi-omic projects or when studying pathomechanisms and causal relationships in suitable disease models.

## Supplementary Information

Below is the link to the electronic supplementary material.

Supplementary file 1 (PDF 11027 KB)

Supplementary file 2 (PDF 370 KB)

Supplementary file 3 (XLSX 134 KB)

## Data Availability

All scripts for pre-processing and analyzing the snATAC-seq data from PSP/CBD frontal cortex and the Allen et al*.* bulkRNA-seq data from PSP TCX are available on GitHub (https://github.com/nes-b/snATAC-seq_psp_cbd). Raw data that support the findings of this study are available in the European Bioinformatics Institute—European Nucleotide Archive (EBI-ENA) under the accession: PRJEB54978.

## Abbreviations

(bv)FTD: (Behavioral variant) frontotemporal dementia
(q)PCR: (Quantitative) polymerase chain reaction
AD: Alzheimer’s disease
ALS: Amyotrophic lateral sclerosis
ARTAG: Aging-related Tau-astrogliopathy
Ast: Astrocytes
ATAC-seq: Assay for transposase-accessible chromatin using sequencing
BH: Benjamini–Hochberg
bp: Base pairs
BP: Biological process
CBD: Corticobasal degeneration
CBS: Corticobasal syndrome
CC: Cellular compartment
CMA: Chaperon-mediated autophagy
CRE: *cis*-Regulatory element
CTSD: Cathepsin D
DAR: Differentially accessible region
DEG: Differentially expressed gene
DLN: Deep-layer neurons
DNA: Desoxyribonucleic acid
Exc.: Excitatory
FDR: False discovery rate
GA: Gene accessibility
Gb: Giga bases
GEM: Gel-bead in emulsion
GO: Gene ontology
GSEA: Gene-set enrichment analysis
GWAS: Genome-wide association study
Inh.: Inhibitory
LB(D): Lewy body (dementia)
Lime: Local interpretable model-agnostic explanations
Log2-FC: Binary logarithm fold-change
MAP(3)K8: Mitogen-activated protein (3) kinase 8
MAPT: Microtubule-associated protein Tau
MF: Molecular function
Mic: Microglia
ML: Machine learning
MND: Motor neuron disease
MSA: Multiple system atrophy
NFT: Neurofibrillary tangles
Oli: Oligodendrocytes
OPC: Oligodendrocytic precursor cells
PART: Primary aging-related tauopathy
PD: Parkinson disease
PMI: *Postmortem* Interval
PSP(-RS): Progressive supranuclear palsy-(Richardson syndrome)
pTau: Hyperphosphorylated tau
RAP: Regulon activity profile
RNA-seq: Ribonucleotide acid sequencing
RTN: Reconstruction of transcriptional regulatory networks
sn*: Single nuclei
SNP: Single nucleotide polymorphism
TA: Tufted astrocyte
TF(M)(E): Transcription factor (motif) (enrichment)
ULN: Upper-layer neurons
UMAP: Uniform manifold approximation and projection
UPR: Unfolded protein response
UPS: Ubiquitin proteasome system
XGB: Extreme gradient boosting
