# Supplementary material for: Single-nucleus chromatin accessibility profiling highlights distinct astrocyte signatures in progressive supranuclear palsy and corticobasal degeneration
Source: Acta Neuropathol. 2022 Aug 17;144(4):615–35. doi: 10.1007/s00401-022-02483-8 (PMC9468099; doi:10.1007/s00401-022-02483-8)
Supplement: Supplementary file 1 — (PDF 11027 KB) [file 401_2022_2483_MOESM1_ESM.pdf]

Single-Nucleus Accessible Chromatin Profiling Highlights Distinct Astrocyte Signatures in Progressive Supranuclear Palsy and Corticobasal Degeneration

Nils Briel, Viktoria C Ruf, Katrin Pratsch, Sigrun Roeber, Jeannine Widmann, Janina Mielke, Mario M Dorostkar, Otto Windl, Thomas Arzberger, Jochen Herms* , Felix L Struebing*

* These authors contributed equally.

– Supplementary figures –

Suppl.Fig.01

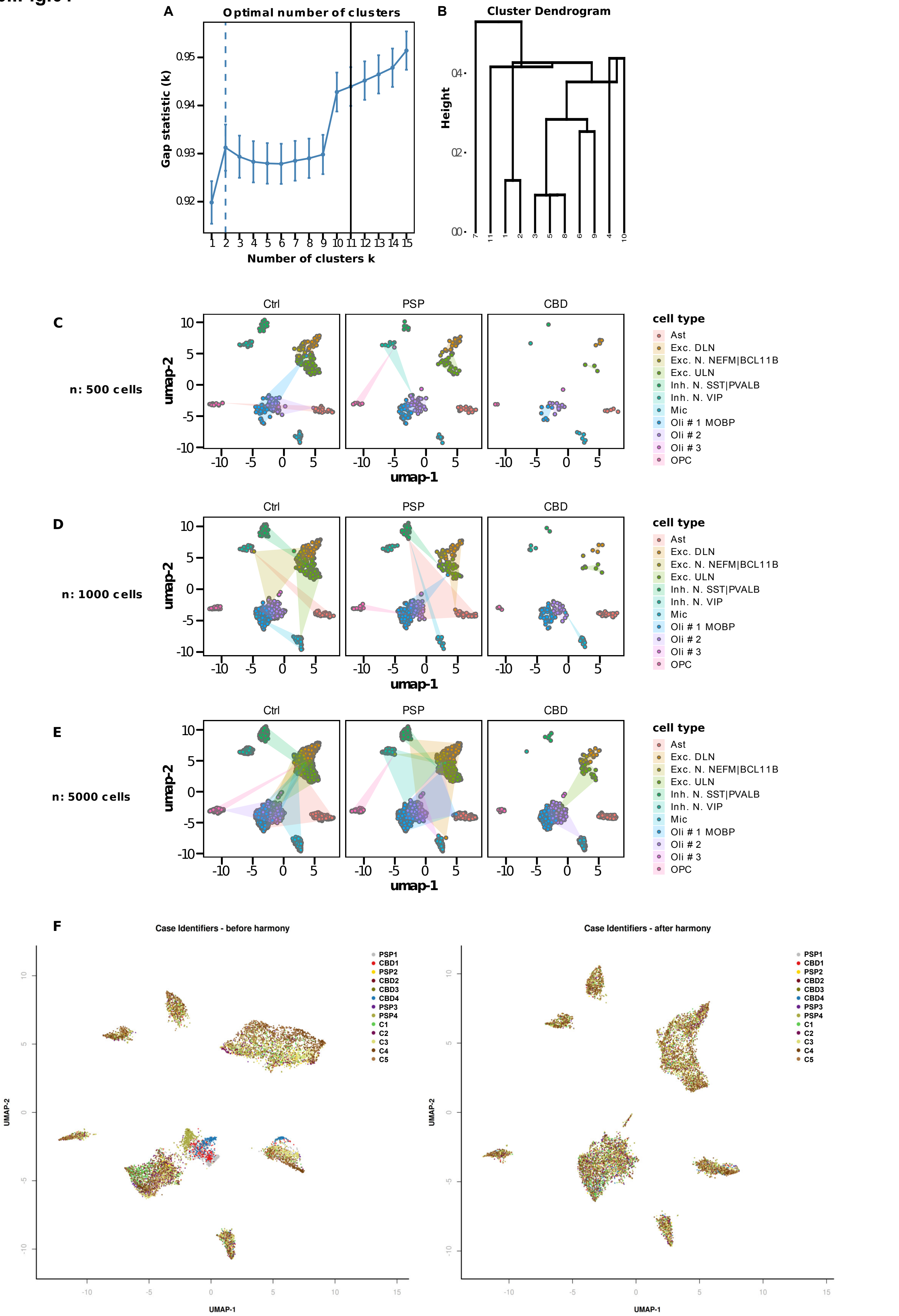

A
Optimal number of clusters
Gap statistic (k)
Number of clusters k
B
Cluster Dendrogram
Height
C
n: 500 cells
D
n: 1000 cells
E
n: 5000 cells
Ctrl
PSP
CBD
umap-1
umap-2
cell type
Ast
Exc. DLN
Exc. N. NEFM|BCL11B
Exc. ULN
Inh. N. SST|PVALB
Inh. N. VIP
Mic
Oli # 1 MOBP
Oli # 2
Oli # 3
OPC
F
Case Identifiers - before harmony
Case Identifiers - after harmony
PSP1
CBD1
PSP2
CBD2
CBD3
CBD4
PSP3
PSP4
C1
C2
C3
C4
C5
UMAP-1
UMAP-2

Suppl.Fig.01 Cluster validation and cluster purity

A Lineplot indicating the *gap statistics* for different *k*-values between 1 and 15 that were drawn from a subset of all barcodes and bin-matrix, *post hoc* confirming a reliable cluster differentiation by the previous graph-based clustering approach. Points and error bars correspond to the deviation of intra-cluster variation at k = 1,…,15 from a randomly distributed reference data set and their standard deviations, respectively. The dendrogram on the right visualizes the determined cluster similarity using centroid hierarchical clustering.
B-D Differentiation of cluster assigned cell type identities in UMAP, stratified by group entity (columns) and n=500,1000, 5000 cells (B,C,D). The color code and polygon fill both indicate cell types and clusters. With increasing cell numbers the observed cluster purity decreases slightly.
F Projection of case identity onto the UMAP embedding of all barcodes before (left) and after batch effect correction using *harmony*.
Abbreviations: Ast, astrocytes; DLN, deep-layer neurons; Exc., excitatory; Inh. N., Inhibitory neurons; Mic, microglia; Neu., neurons; ULN, upper-layer neurons; Oli Oligodendrocytes; OPC, oligodendrocytic precursor cells.

Suppl.Fig.02

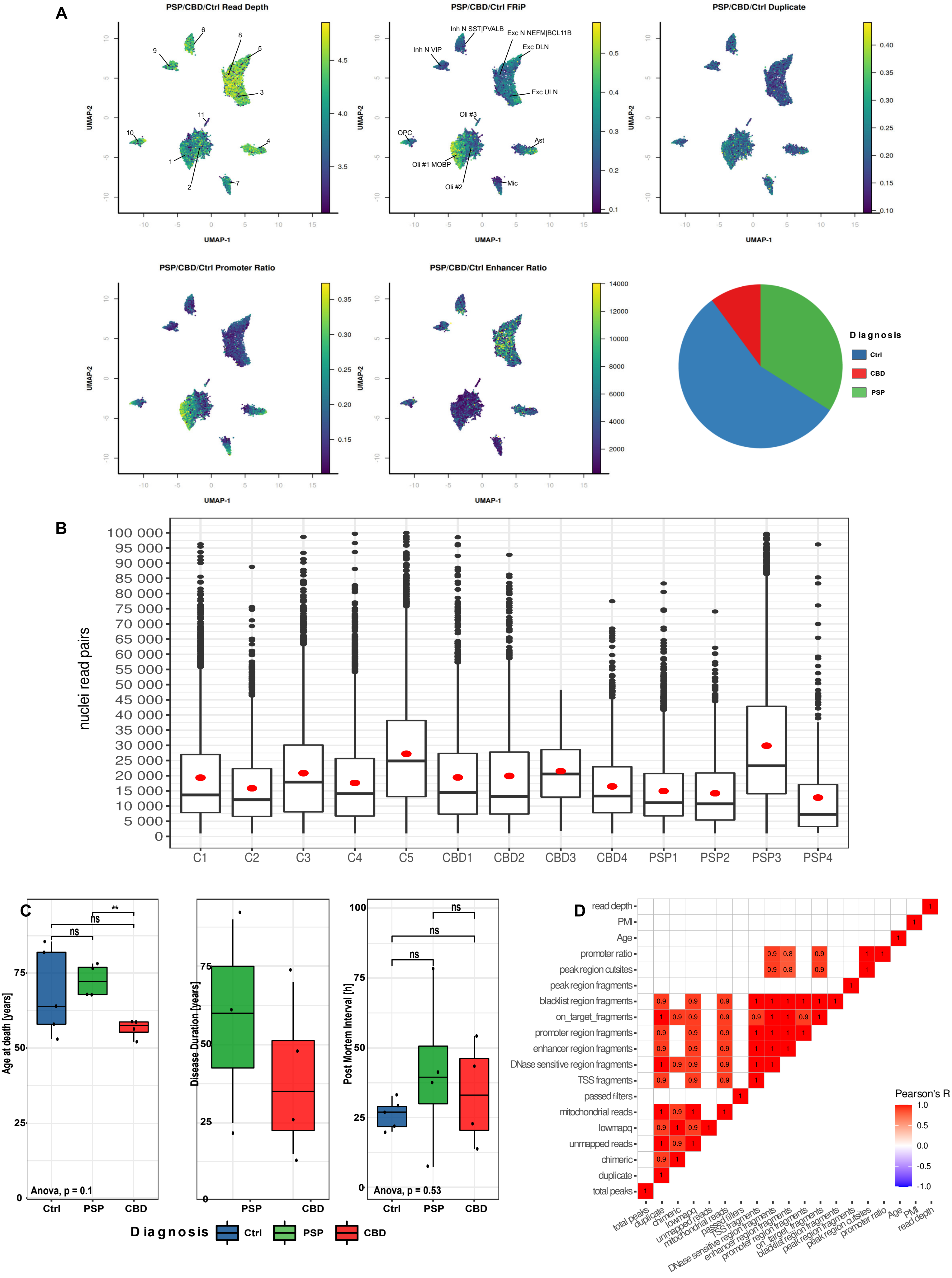
A
PSP/CBD/Ctrl Read Depth
PSP/CBD/Ctrl FRiP
PSP/CBD/Ctrl Duplicate
PSP/CBD/Ctrl Promoter Ratio
PSP/CBD/Ctrl Enhancer Ratio
UMAP-1
UMAP-2
Inh N SST|PVALB
Exc N NEFM|BCL11B
Inh N VIP
Exc DLN
Exc ULN
Oli #3
OPC
Ast
Oli #1 MOBP
Oli #2
Mic
Diagnosis
Ctrl
CBD
PSP
B
nuclei read pairs
C1
C2
C3
C4
C5
CBD1
CBD2
CBD3
CBD4
PSP1
PSP2
PSP3
PSP4
C
Age at death [years]
Disease Duration [years]
Post Mortem Interval [h]
Anova, p = 0.1
Anova, p = 0.53
ns
**
Diagnosis
Ctrl
PSP
CBD
D
read depth
PMI
Age
promoter ratio
peak region cutsites
peak region fragments
blacklist region fragments
on_target_fragments
promoter region fragments
enhancer region fragments
DNase sensitive region fragments
TSS fragments
passed filters
mitochondrial reads
lowmapq
unmapped reads
chimeric
duplicate
total peaks
Pearson's R

Suppl.Fig.02 Epidemiological and sequencing metadata

A Projections of technical metadata onto the UMAP embedding of high quality-filtered barcodes indicating the respective variable as color code. Shown are read depth, fraction of reads in peaks (‘FriP’) score, duplicate likelihood, promoter and enhancer ratios. A pie chart displays group-wise contributions to the entire cell pool. As reference, the first an second panel include cluster identifiers (ID) and cell type assignments, respectively.
B Boxplot comparisons of nuclei sequencing read depth across included cases. Outliers are depicted as black dots. The hinges of each box correspond to the 25th and 75th percentiles with medians drawn as black bar. The 1.5-times inter-quartile ranges are shown as black whiskers. Red dots show means of the distributions.
C Boxplot comparisons of patient-specific and disease-relevant parameters, such as age at death, disease duration and *post mortem* interval. The plot structure equals to B. Color coding indicates the neuropathological diagnose, while asterisks denote the degree of significance with ‘ns’ = ‘not significant’, *p<.05, and **p<.01. Analysis of variance (Anova) results are shown at the bottom of each triple comparison.
D Correlation matrix heatmap of epidemiological and technical parameters between average-aggregated barcodes of single human cases. *Pearson’s* R is displayed by the color shading and labels. Paired correlations that do not comply with a p<.05 are shown as empty boxes.
Abbreviations: PMI, *post mortem* interval; TSS, transcription starting site; UMAP, uniform manifold approximation and projection.

Suppl.Fig.03

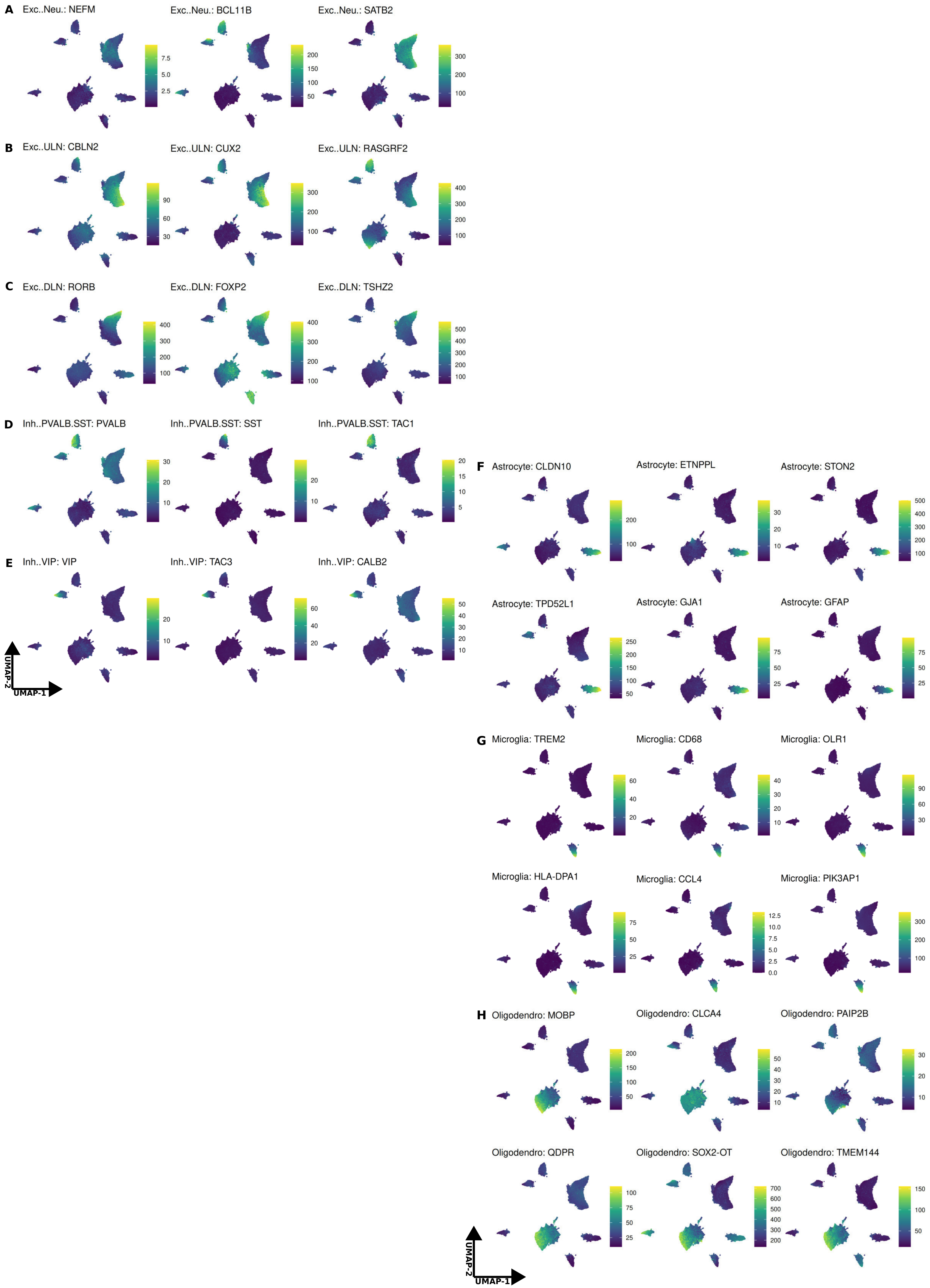
A
Exc..Neu.: NEFM
7.5
5.0
2.5
Exc..Neu.: BCL11B
200
150
100
50
Exc..Neu.: SATB2
300
200
100
B
Exc..ULN: CBLN2
90
60
30
Exc..ULN: CUX2
300
200
100
Exc..ULN: RASGRF2
400
300
200
100
C
Exc..DLN: RORB
400
300
200
100
Exc..DLN: FOXP2
400
300
200
100
Exc..DLN: TSHZ2
500
400
300
200
100
D
Inh..PVALB.SST: PVALB
30
20
10
Inh..PVALB.SST: SST
20
10
Inh..PVALB.SST: TAC1
20
15
10
5
E
Inh..VIP: VIP
20
10
Inh..VIP: TAC3
60
40
20
Inh..VIP: CALB2
50
40
30
20
10
UMAP-2
UMAP-1
F
Astrocyte: CLDN10
200
100
Astrocyte: ETNPPL
30
20
10
Astrocyte: STON2
500
400
300
200
100
Astrocyte: TPD52L1
250
200
150
100
50
Astrocyte: GJA1
75
50
25
Astrocyte: GFAP
75
50
25
G
Microglia: TREM2
60
40
20
Microglia: CD68
40
30
20
10
Microglia: OLR1
90
60
30
Microglia: HLA-DPA1
75
50
25
Microglia: CCL4
12.5
10.0
7.5
5.0
2.5
0.0
Microglia: PIK3AP1
300
200
100
H
Oligodendro: MOBP
200
150
100
50
Oligodendro: CLCA4
50
40
30
20
10
Oligodendro: PAIP2B
30
20
10
Oligodendro: QDPR
100
75
50
25
Oligodendro: SOX2-OT
700
600
500
400
300
200
Oligodendro: TMEM144
150
100
50
UMAP-2
UMAP-1

Suppl.Fig.03 Cell type identification by marker gene activity

Projections of accessibility at canonical *neuronal* (**A-E**) and *glial* (**F-H**) marker genes onto the UMAP embedding of barcodes indicating the respective variable as color code from low (blue) to high (yellow). Depicted are the three most most distinguishing marker genes of each cell type or sub-type. GA scores are scaled to each gene’s range.
Abbreviations: DLN, deep-layer neurons; Exc., excitatory; Inh. Inhibitory neurons; Neu., neurons; Oligodendro, oligodendrocytes; ULN, upper-layer neurons.

Suppl.Fig.04

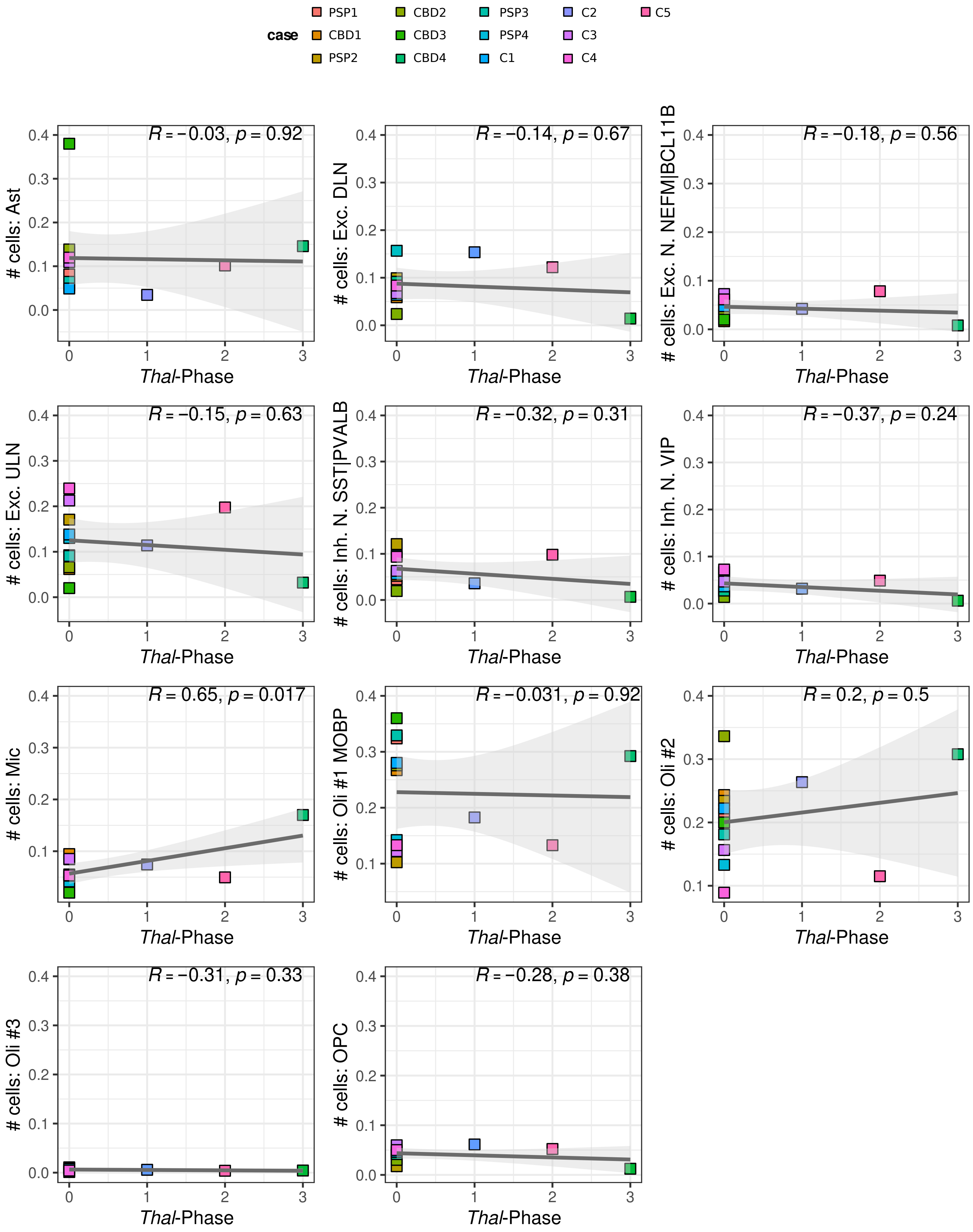

case
PSP1
CBD1
PSP2
CBD2
CBD3
CBD4
PSP3
PSP4
C1
C2
C3
C4
C5
R = −0.03, p = 0.92
# cells: Ast
R = −0.14, p = 0.67
# cells: Exc. DLN
R = −0.18, p = 0.56
# cells: Exc. N. NEFM|BCL11B
R = −0.15, p = 0.63
# cells: Exc. ULN
R = −0.32, p = 0.31
# cells: Inh. N. SST|PVALB
R = −0.37, p = 0.24
# cells: Inh. N. VIP
R = 0.65, p = 0.017
# cells: Mic
R = −0.031, p = 0.92
# cells: Oli #1 MOBP
R = 0.2, p = 0.5
# cells: Oli #2
R = −0.31, p = 0.33
# cells: Oli #3
R = −0.28, p = 0.38
# cells: OPC
Thal-Phase

Suppl.Fig.04 Cross-case correlation of cell type frequencies and *Thal* phases

Stratified by previously defined cell types and subpopulations, each scatter plot depicts the relation between *Thal*-phase (Aβ+ plaque distribution) on the x-axis and the relative cell type frequency across cases in a group-agnostic way. A regression line is drawn in black, while the confidence interval is shown in grey. *Pearson's* R and p-values are depicted in the upper right corners. Color code indicates case identity.

Suppl.Fig.05

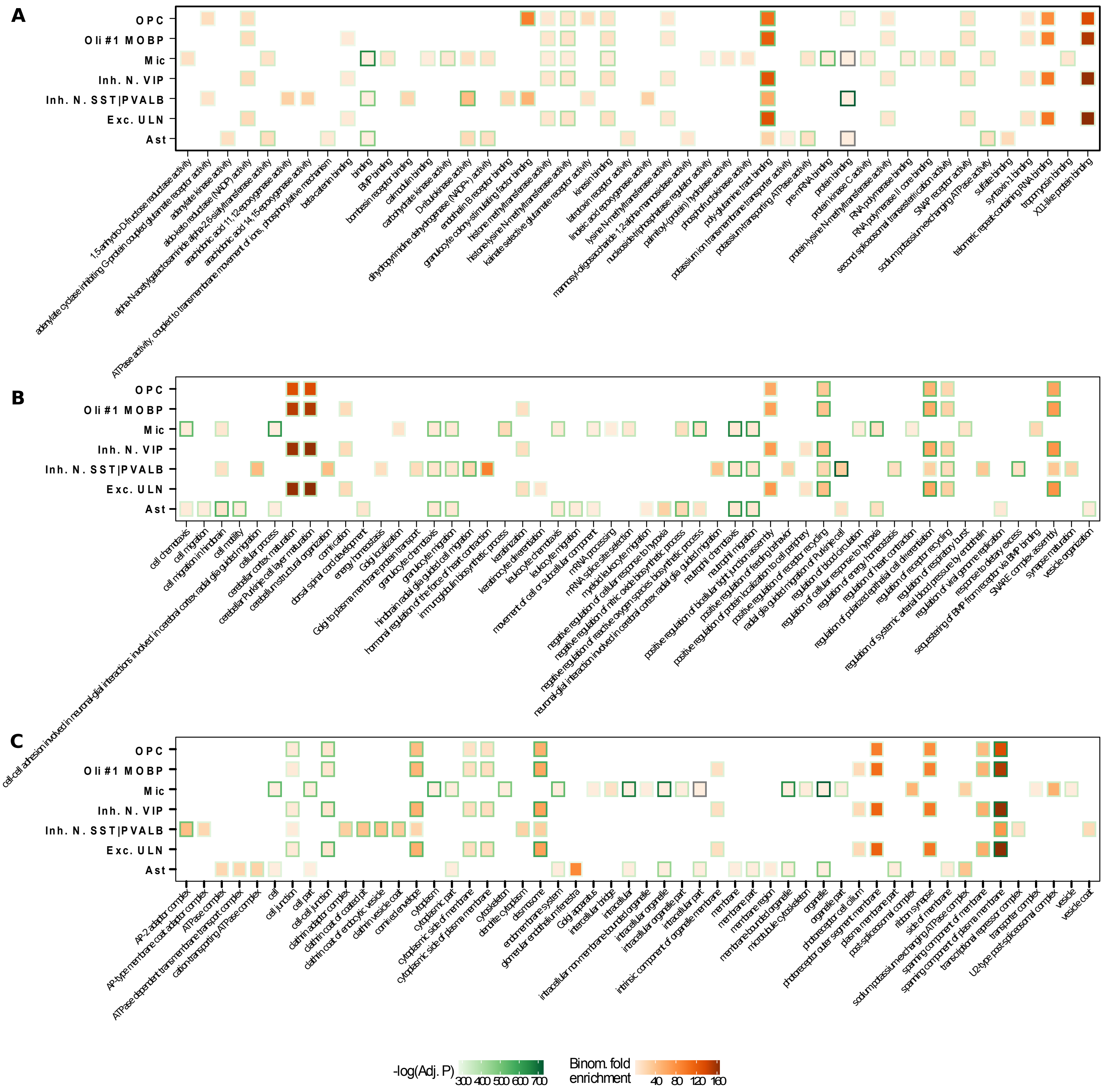
A
OPC
Oli #1 MOBP
Mic
Inh. N. VIP
Inh. N. SST|PVALB
Exc. ULN
Ast
1,5-anhydro-D-fructose reductase activity
adenylate cyclase inhibiting G-protein coupled glutamate receptor activity
adenylate kinase activity
aldo-keto reductase (NADP) activity
alpha-N-acetylgalactosaminide alpha-2,6-sialyltransferase activity
arachidonic acid 11,12-epoxygenase activity
arachidonic acid 14,15-epoxygenase activity
ATPase activity, coupled to transmembrane movement of ions, phosphorylative mechanism
beta-catenin binding
binding
BMP binding
bombesin receptor binding
calmodulin binding
carbohydrate kinase activity
D-ribulokinase activity
dihydropyrimidine dehydrogenase (NADP+) activity
endothelin B receptor binding
granulocyte colony-stimulating factor binding
histone methyltransferase activity
histone-lysine N-methyltransferase activity
kainate selective glutamate receptor activity
kinesin binding
latrotoxin receptor activity
linoleic acid epoxygenase activity
lysine N-methyltransferase activity
mannosyl-oligosaccharide 1,2-alpha-mannosidase activity
nucleoside-triphosphatase regulator activity
palmitoyl-(protein) hydrolase activity
phosphofructokinase activity
poly-glutamine tract binding
potassium ion transmembrane transporter activity
potassium-transporting ATPase activity
pre-mRNA binding
protein binding
protein kinase C activity
protein-lysine N-methyltransferase activity
RNA polymerase binding
RNA polymerase II core binding
second spliceosomal transesterification activity
SNAP receptor activity
sodium:potassium-exchanging ATPase activity
sulfate binding
Syntaxin-1 binding
telomeric repeat-containing RNA binding
tropomyosin binding
XCL-like protein binding
B
OPC
Oli #1 MOBP
Mic
Inh. N. VIP
Inh. N. SST|PVALB
Exc. ULN
Ast
cell chemotaxis
cell migration
cell migration in hindbrain
cell motility
cell-cell adhesion involved in neuronal-glial interactions involved in cerebral cortex radial glia guided migration
cellular process
cerebellar cortex maturation
cerebellar Purkinje cell layer maturation
cerebellum structural organization
cornification
dorsal spinal cord development
energy homeostasis
Golgi localization
Golgi to plasma membrane protein transport
granulocyte chemotaxis
granulocyte migration
hindbrain radial glia guided cell migration
hormonal regulation of the force of heart contraction
immunoglobulin biosynthetic process
keratinization
keratinocyte differentiation
leukocyte chemotaxis
leukocyte migration
movement of cell or subcellular component
mRNA processing
mRNA splice site selection
myeloid leukocyte migration
negative regulation of cellular response to hypoxia
negative regulation of nitric oxide biosynthetic process
negative regulation of reactive oxygen species biosynthetic process
neuronal-glial interaction involved in cerebral cortex radial glia guided migration
neutrophil chemotaxis
neutrophil migration
positive regulation of bicellular tight junction assembly
positive regulation of feeding behavior
positive regulation of protein localization to cell periphery
positive regulation of receptor recycling
radial glia guided migration of Purkinje cell
regulation of blood circulation
regulation of cellular response to hypoxia
regulation of energy homeostasis
regulation of heart contraction
regulation of polarized epithelial cell differentiation
regulation of receptor recycling
regulation of respiratory burst
regulation of systemic arterial blood pressure by endothelin
regulation of viral genome replication
response to dietary excess
sequestering of BMP from receptor via BMP binding
SNARE complex assembly
synapse maturation
vesicle organization
C
OPC
Oli #1 MOBP
Mic
Inh. N. VIP
Inh. N. SST|PVALB
Exc. ULN
Ast
AP-2 adaptor complex
AP-type membrane coat adaptor complex
ATPase complex
ATPase dependent transmembrane transport complex
cation-transporting ATPase complex
cell
cell junction
cell part
cell-cell junction
clathrin adaptor complex
clathrin coat of coated pit
clathrin coat of endocytic vesicle
clathrin vesicle coat
cornified envelope
cytoplasm
cytoplasmic part
cytoplasmic side of membrane
cytoplasmic side of plasma membrane
cytoskeleton
dendritic cytoplasm
desmosome
endomembrane system
glomerular endothelium fenestra
Golgi apparatus
intercellular bridge
intracellular
intracellular non-membrane-bounded organelle
intracellular organelle
intracellular organelle part
intracellular part
intrinsic component of organelle membrane
membrane
membrane part
membrane region
membrane-bounded organelle
microtubule cytoskeleton
organelle
organelle part
photoreceptor cell cilium
photoreceptor outer segment membrane
plasma membrane part
post-spliceosomal complex
ribbon synapse
side of membrane
sodium:potassium-exchanging ATPase complex
spanning component of membrane
spanning component of plasma membrane
transcriptional repressor complex
transporter complex
U2-type post-spliceosomal complex
vesicle
vesicle coat
-log(Adj. P)
300 400 500 600 700
Binom. fold enrichment
40 80 120 160

Suppl.Fig.05 Gene ontology (GO) of the major brain cell types

A-C GO tile maps of the top 100 results of each category Molecular Funciton (MF, **A**), Biological Process (BP, **B**) and Cellular Compartment (CC, **C**). The results were ranked by their *Benjamini Hochberg*-corrected p-values of the binomial enrichment test in *rGREAT*. Every column corresponds to one of these terms and every row to the cell type under investigation. The color fill indicates the binomial test fold enrichment, while the colored outlines show the negative decadic logarithm of the adjusted p-values (Adj. P).
Abbreviations: Adj. P, adjusted p-value; Ast, astrocytes; binom, binomial; DLN, deep-layer neurons; Exc., excitatory; Inh. N., Inhibitory neurons; Mic, microglia; Neu., neurons; ULN, upper-layer neurons; Oli Oligodendrocytes; OPC, oligodendrocytic precursor cells.
.

Suppl.Fig.06

A

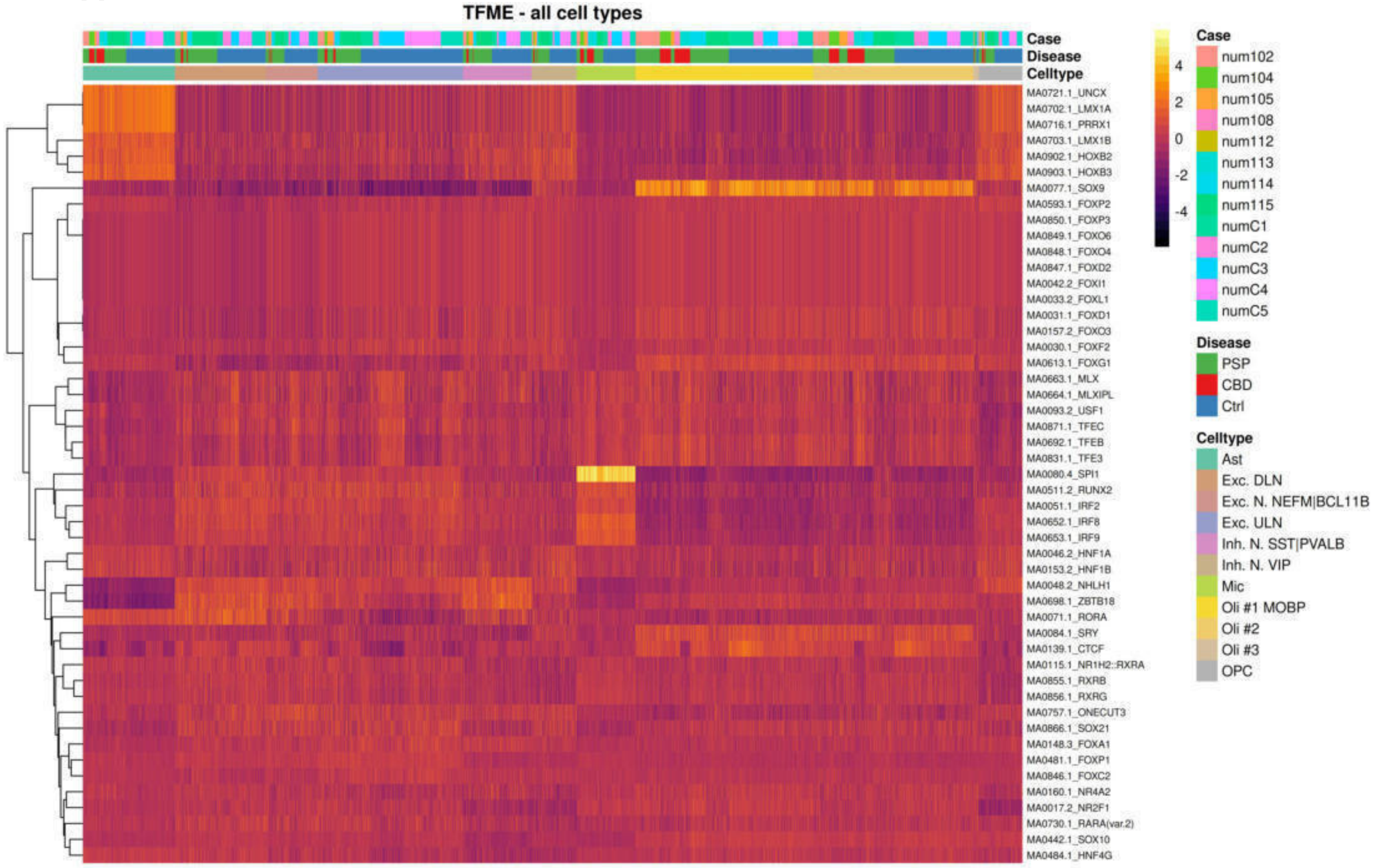

B

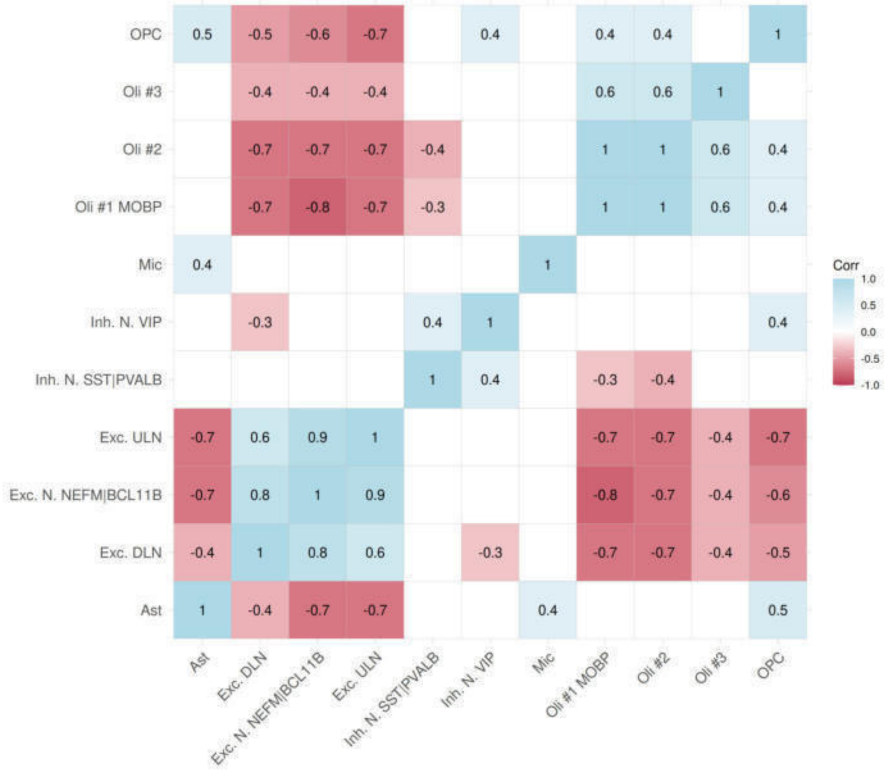

Suppl.Fig.06 TF profile in the tauopathy data set

A Motif enrichment heatmap of a TF subset that characterizes brain cell types. Every column corresponds to a single barcode and every row to a TF motif, while the color gradient indicates the extent of TF motif enrichment (TFME) from negative (black) through intermediate (purple) to high (yellow) values. Rows were clustered hierarchically (*Manhattan* distance, *Ward-D2* method) and results indicated as dendrogram on the left. The column order was fixed while the colored bars at the top inform about the case, disease diagnosis and cell type. Gene names comply with the *Ensembl* identifiers.
B Heatmap of correlation testing between cell type-wise average TFME values. *Spearman* correlation coefficient is displayed by the color shading and labels. Paired correlations that do not comply with a $p<.05$ are shown as empty boxes.
Abbreviations: Ast, astrocytes; Corr, correlation coefficient; DLN, deep-layer neurons; Exc., excitatory; Inh. N., Inhibitory neurons; Mic, microglia; Neu., neurons; ULN, upper-layer neurons; Oli Oligodendrocytes; OPC, oligodendrocytic precursor cells.

Suppl.Fig.07

A

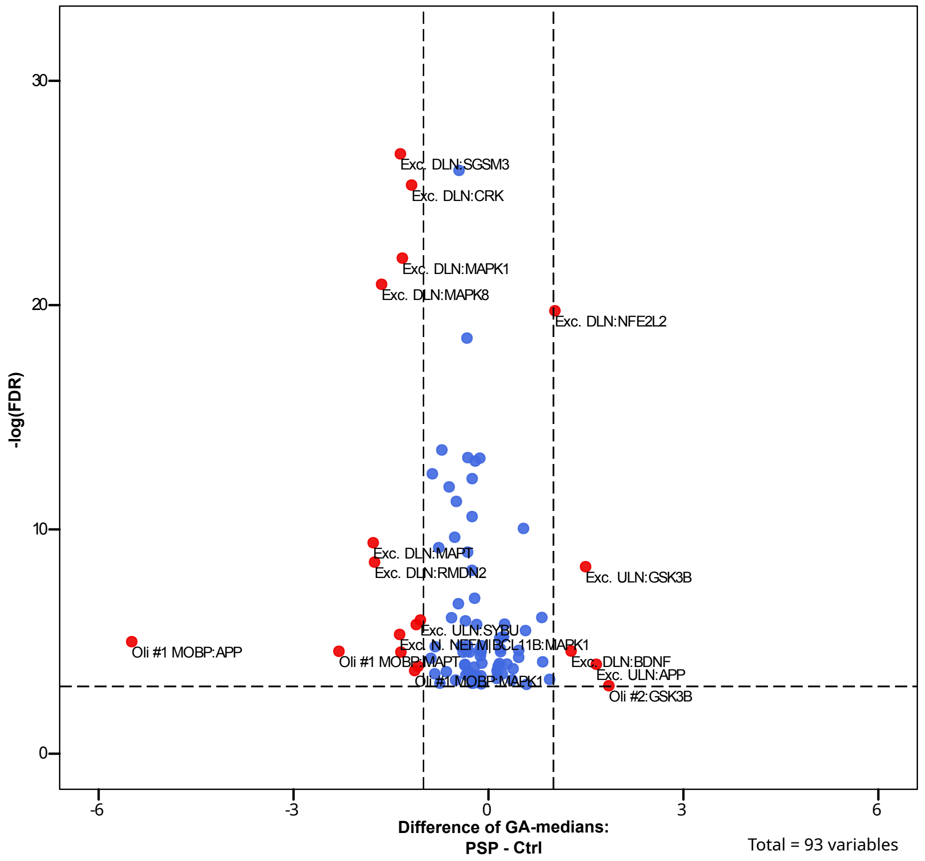

B

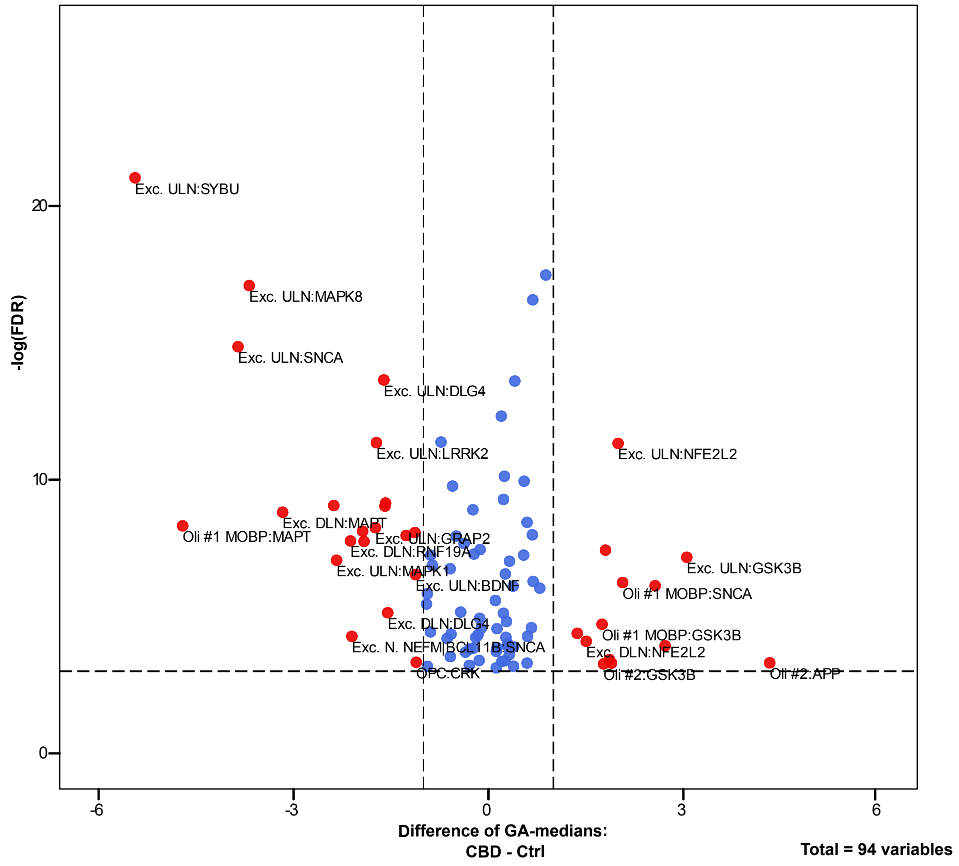

Suppl.Fig.07 Accessibility alterations at tauopathy-associated genes

A Volcano plot of differentially accessible tauopathy-associated genes in the PSP frontal cortex resolved by cell type assignments. Resulting from pair-wise Wilcoxon rank sum tests, the 93 significant tauopathy gene - cell type pairs (GCPs) are depicted in the plot. The x-axis differentiates the GA difference between the PSP GCPs and the reference GCPs of the Ctrl group. The y-axis displays the negative decadic logarithm of the FDR-corrected p-values. Color codes indicates whether GCPs comply with an adj. p-value <= .05 and an absolute difference of GA medians >=1 (red) or not (blue).
B Volcano plot of differentially accessible tauopathy-associated genes in the CBD frontal cortex resolved by cell type assignments. Resulting from pair-wise *Wilcoxon* rank sum tests, the 94 significant GCPs are distributed in the plot. The general plot structure corresponds to C.
Abbreviations: Adj. P, adjusted p-value; Ast, astrocytes; FDR, false discovery rate; GA, gene accessibility.

Suppl.Fig.08

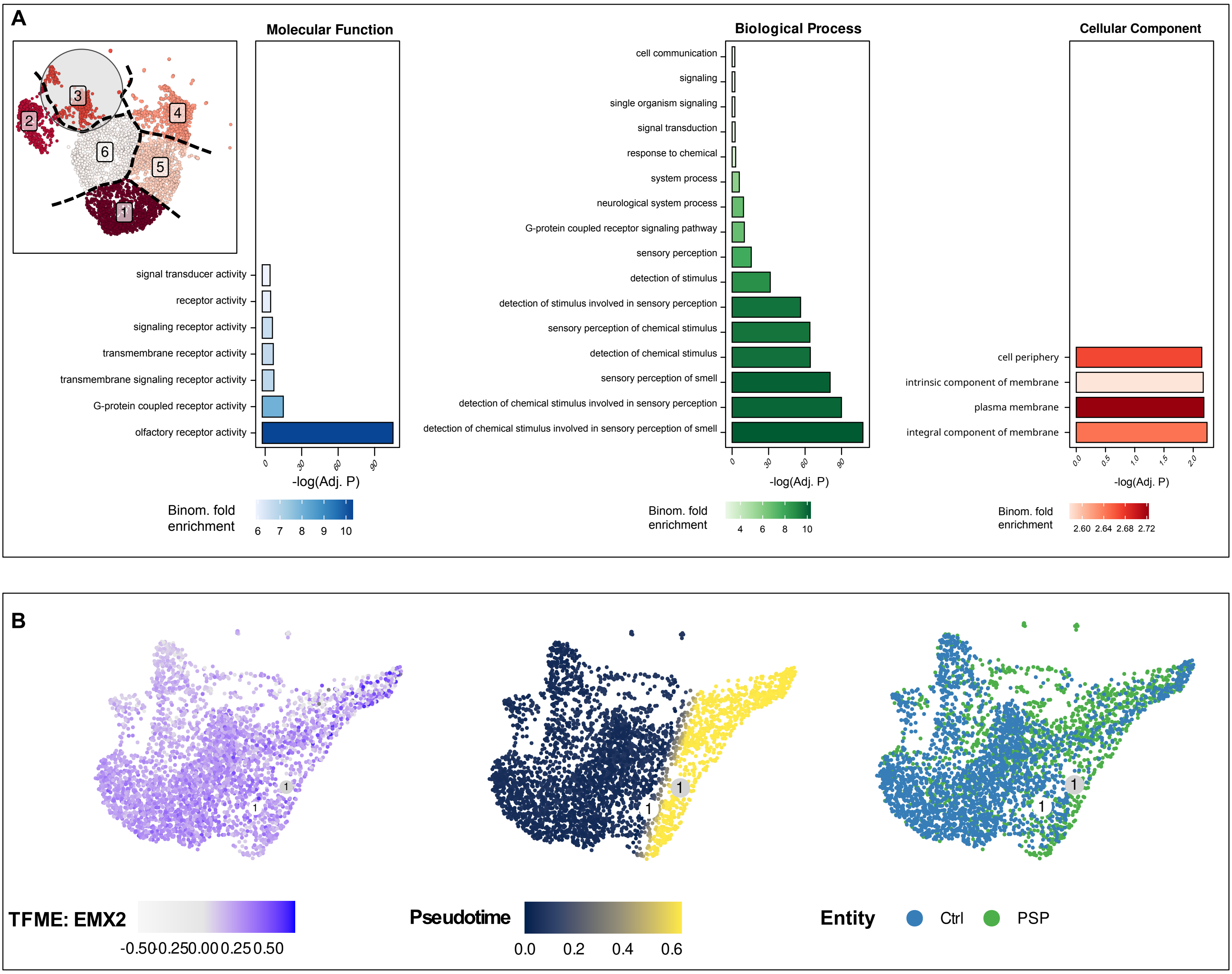
A
Molecular Function
signal transducer activity
receptor activity
signaling receptor activity
transmembrane receptor activity
transmembrane signaling receptor activity
G-protein coupled receptor activity
olfactory receptor activity
-log(Adj. P)
Binom. fold enrichment
6 7 8 9 10
Biological Process
cell communication
signaling
single organism signaling
signal transduction
response to chemical
system process
neurological system process
G-protein coupled receptor signaling pathway
sensory perception
detection of stimulus
detection of stimulus involved in sensory perception
sensory perception of chemical stimulus
detection of chemical stimulus
sensory perception of smell
detection of chemical stimulus involved in sensory perception
detection of chemical stimulus involved in sensory perception of smell
-log(Adj. P)
Binom. fold enrichment
4 6 8 10
Cellular Component
cell periphery
intrinsic component of membrane
plasma membrane
integral component of membrane
-log(Adj. P)
Binom. fold enrichment
2.60 2.64 2.68 2.72
B
TFME: EMX2
-0.50 -0.25 0.00 0.25 0.50
Pseudotime
0.0 0.2 0.4 0.6
Entity
Ctrl
PSP

Suppl.Fig.08 GO of the astrocyte subclusters

A GO analysis results in the astrocytic subcluster 3 after k-means clustering of the PSP, CBD, and Ctrl astrocytes-comprising UMAP. Bar plots of the top results of each category MF (left), BP (**mid**) and CC (right) were ranked by their *Benjamini Hochberg*-corrected p-values of the binomial enrichment test in *rGREAT*. Every row corresponds to one of the GO terms and the color fill indicates the binomial test fold enrichment, while the bar length represents the negative decadic logarithm of the adjusted p-values (Adj. P).
B Subset of PSP and Ctrl astrocytes re-embedded in UMAP. Color code indicates EMX2 TF motif enrichment (TFME) (left), inferred pseudotime (mid), or group entity (right). The black line indicates the pseudotemporal trajectory from the 'root' (white label) toward the 'end' (grey label) cell. A clear transition trajectory could not be inferred.
Abbreviations: Adj. P, adjusted p-value; Ast, astrocytes; binom, binomial.

Suppl.Fig.09

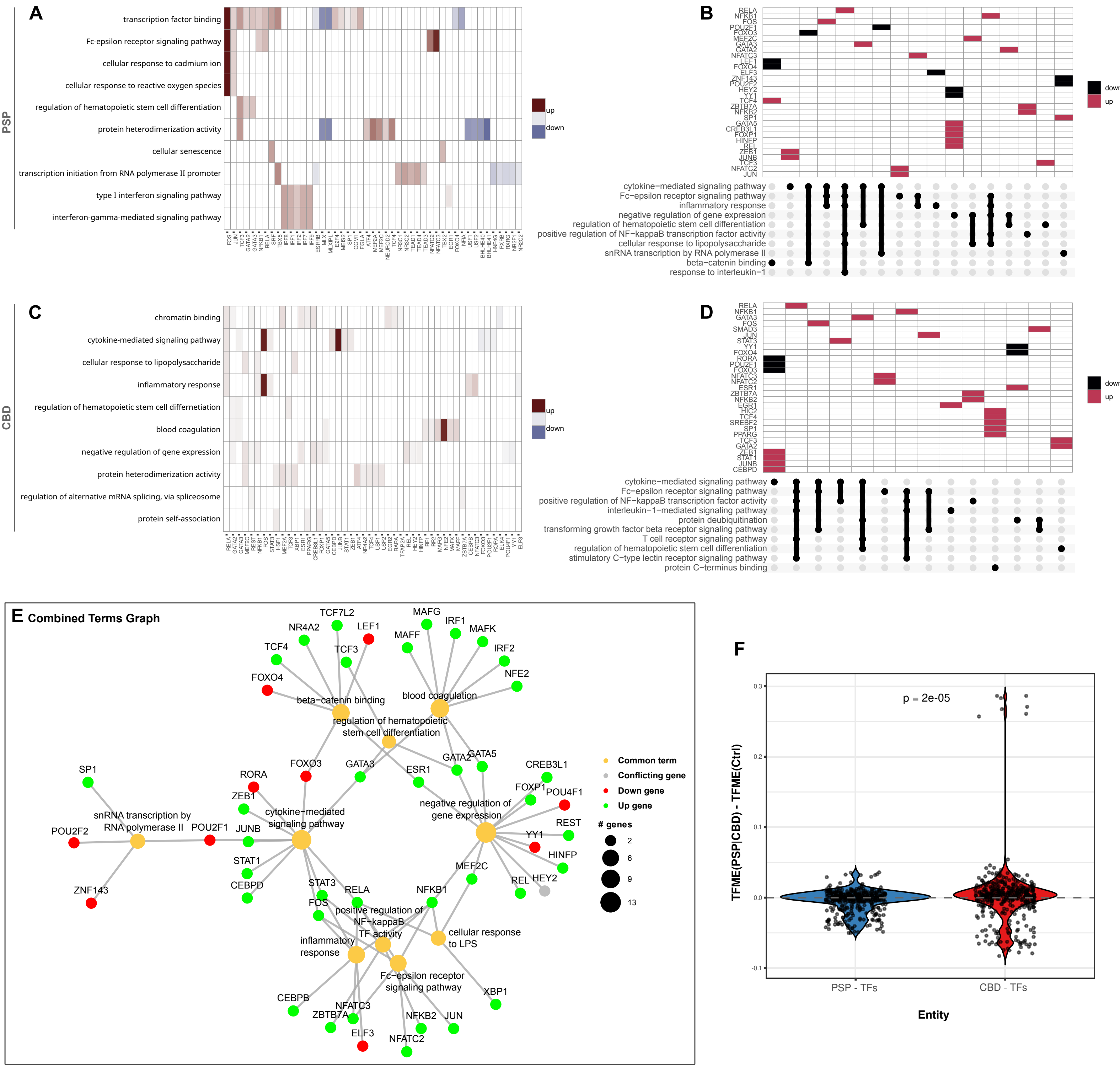

A
PSP
transcription factor binding
Fc-epsilon receptor signaling pathway
cellular response to cadmium ion
cellular response to reactive oxygen species
regulation of hematopoietic stem cell differentiation
protein heterodimerization activity
cellular senescence
transcription initiation from RNA polymerase II promoter
type I interferon signaling pathway
interferon-gamma-mediated signaling pathway
up
down
B
down
up
cytokine−mediated signaling pathway
Fc−epsilon receptor signaling pathway
inflammatory response
negative regulation of gene expression
regulation of hematopoietic stem cell differentiation
positive regulation of NF−kappaB transcription factor activity
cellular response to lipopolysaccharide
snRNA transcription by RNA polymerase II
beta−catenin binding
response to interleukin−1
C
CBD
chromatin binding
cytokine-mediated signaling pathway
cellular response to lipopolysaccharide
inflammatory response
regulation of hematopoietic stem cell differentiation
blood coagulation
negative regulation of gene expression
protein heterodimerization activity
regulation of alternative mRNA splicing, via spliceosome
protein self-association
up
down
D
down
up
cytokine−mediated signaling pathway
Fc−epsilon receptor signaling pathway
positive regulation of NF−kappaB transcription factor activity
interleukin−1−mediated signaling pathway
protein deubiquitination
transforming growth factor beta receptor signaling pathway
T cell receptor signaling pathway
regulation of hematopoietic stem cell differentiation
stimulatory C−type lectin receptor signaling pathway
protein C−terminus binding
E Combined Terms Graph
beta−catenin binding
blood coagulation
regulation of hematopoietic stem cell differentiation
snRNA transcription by RNA polymerase II
cytokine−mediated signaling pathway
negative regulation of gene expression
positive regulation of NF−kappaB TF activity
inflammatory response
cellular response to LPS
Fc−epsilon receptor signaling pathway
Common term
Conflicting gene
Down gene
Up gene
genes
2
6
9
13
F
p = 2e-05
TFME(PSP|CBD) - TFME(Ctrl)
PSP - TFs
CBD - TFs
Entity

Suppl.Fig.09 Biological pathways indicated by altered TFs in tauopathy brains

A GO enrichment analysis of TFs that exhibited significant TFME deviations in PSP astrocytes. Only the top 25 enrichment results with an adjusted p-value <.05 are depicted. Rows correspond to the GO terms comprising MF, BP, and CC, while TF *Ensembl* IDs are given on the x-axis. The color shading indicates the enrichment score from negative (blue) through zero (grey) to high (red) values.
B GO enrichment analysis of TFs that exhibited significant TFME deviations in CBD astrocytes. The plot structure equals to A.
C Combined heatmap-upset plot of the top 10 GO terms enriched in the set of differentially active TFs in PSP astrocytes. The lower matrix shows the co-enriched GO term logic. The resulting intersection of GO terms involves those TFs that are highlighted in the heatmap above. Whether these TFs exhibit positive or negative TFME values changes is indicated by the color code.
D Combined heatmap-upset plot of the top 10 GO terms enriched in the set of differentially active TFs in CBD astrocytes. The plot structure equals to C.
E Bubble-connection graph depicting the top 10 co-enriched GO terms of significant TF alterations in PSP and CBD. The yellow dots represent specific GO terms, while their size equal to the number of term-assigned genes (# genes) that are found in the queried data sets. These genes are displayed as red (co-downregulated), green (co-upregulated), or grey dots (conflicting direction).
F The extent of TFME alterations differs between astrocytes of PSP and CBD origin. Y-axes depict TFME median value deviations from Ctrls' medians in astrocytes. The dots represent single TFMs, while violin convexities indicate the distribution over TFME deviations. For a more intuitive comprehension a horizontal line marks the zero line. The degree of statistical significance is given for the CBD vs. PSP comparison (*Wilcoxon* rank-sum test).

Suppl.Fig.10

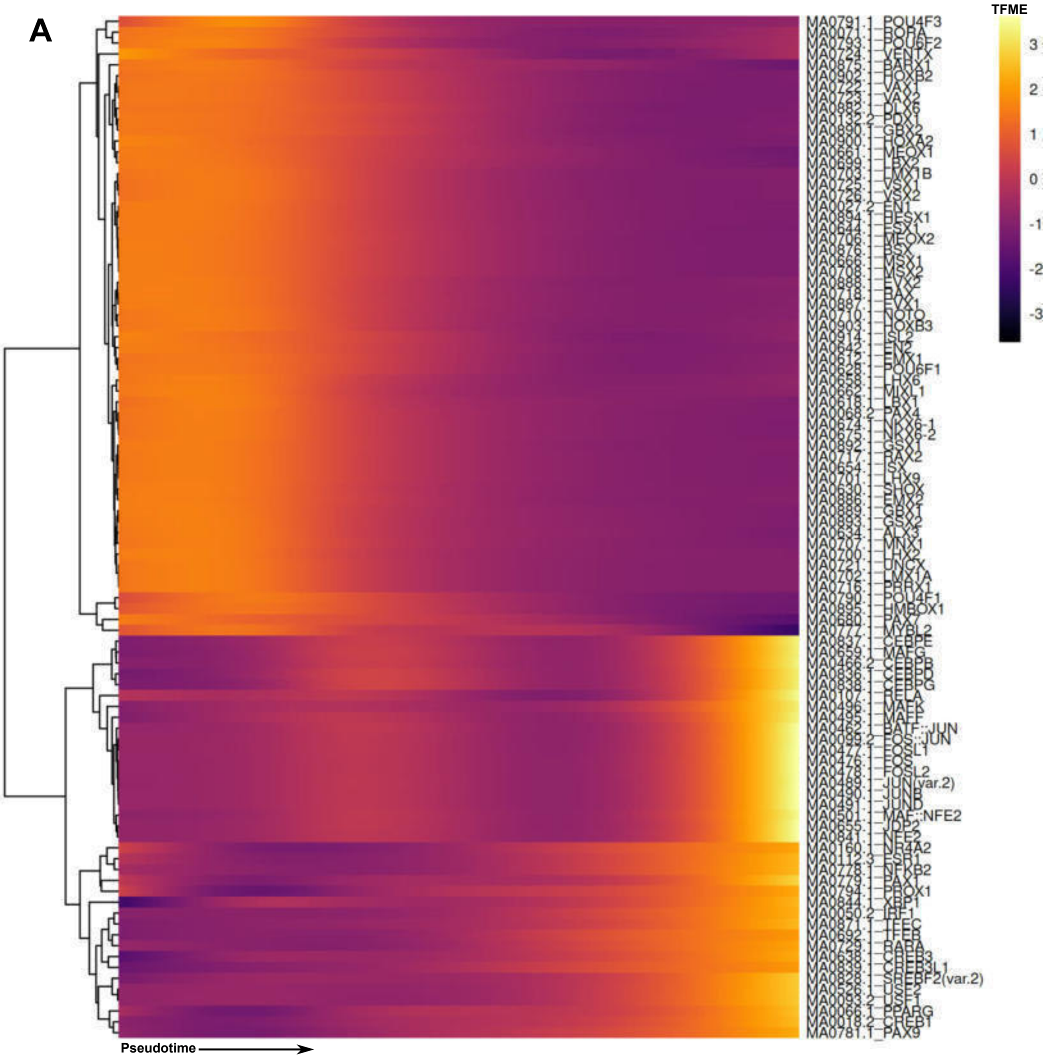
A
TFME
3
2
1
0
-1
-2
-3
Pseudotime

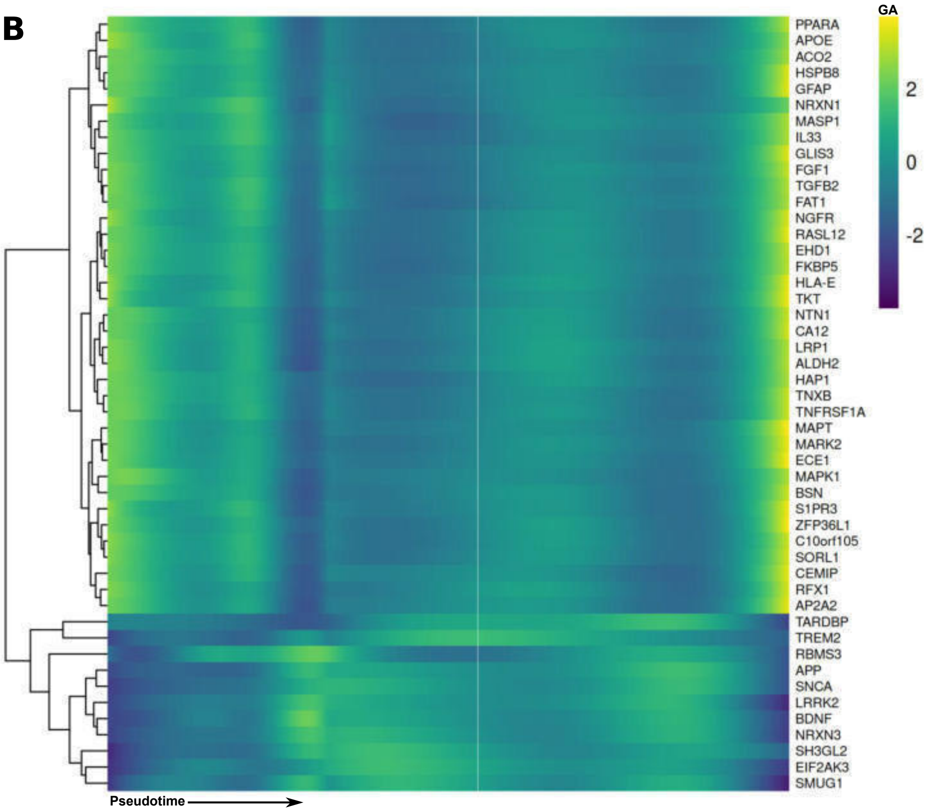
B
GA
2
0
-2
PPARA
APOE
ACO2
HSPB8
GFAP
NRXN1
MASP1
IL33
GLIS3
FGF1
TGFB2
FAT1
NGFR
RASL12
EHD1
FKBP5
HLA-E
TKT
NTN1
CA12
LRP1
ALDH2
HAP1
TNXB
TNFRSF1A
MAPT
MARK2
ECE1
MAPK1
BSN
S1PR3
ZFP36L1
C10orf105
SORL1
CEMIP
RFX1
AP2A2
TARDBP
TREM2
RBMS3
APP
SNCA
LRRK2
BDNF
NRXN3
SH3GL2
EIF2AK3
SMUG1
Pseudotime

Suppl.Fig.10 Loss of immaturity markers and acquisition of a reactive inflammatory state

A Heatmap displaying the pseudotime changes in TFME of the TFs belonging to the two major, inverse running clusters as determined by k-means clustering in *tradeSeq*. Results relate to Ctrl/CBD astrocytes and their pseudotemporal transition only. TFME courses (rows) were clustered hierarchically (*Euclidean* distance, c*omplete* method) and results indicated as dendrogram on the left.
B Heatmap displaying the pseudotime changes in GA of tauopathy-associated as well as significantly altered genes in the start-vs.-end and association test in *tradeSeq.* Candidates had to comply fulfill the criterion of p<.05 (Wald-statistic, *Bonferroni* correction). The results relate to Ctrl/CBD astrocytes and their pseudotemporal transition only. GA courses (rows) were clustered hierarchically (*Euclidean* distance, c*omplete* method) and results indicated as dendrogram on the left.

Suppl.Fig.11

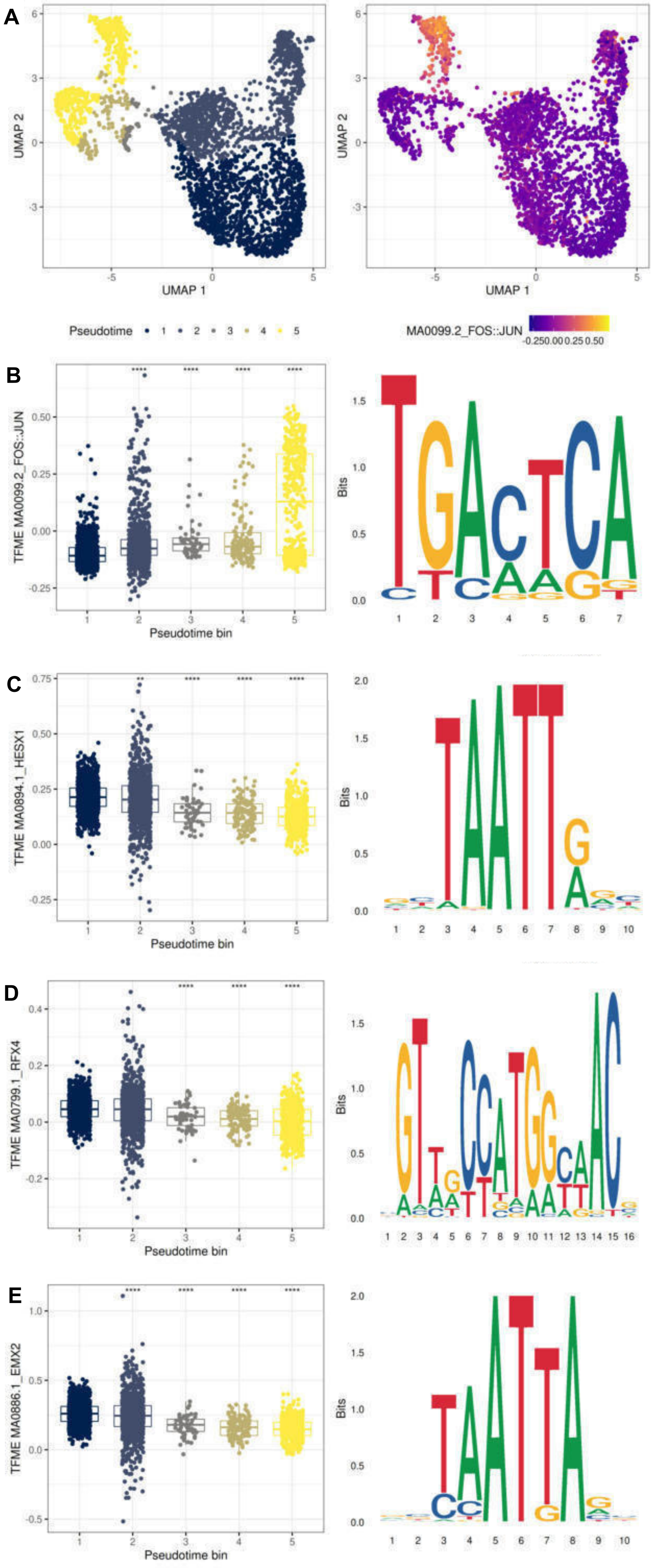

A
UMAP 1
UMAP 2
Pseudotime 1 2 3 4 5
MA0099.2_FOS::JUN
-0.25 0.00 0.25 0.50
B
TFME MA0099.2_FOS::JUN
Pseudotime bin
Bits
C
TFME MA0894.1_HESX1
Pseudotime bin
Bits
D
TFME MA0799.1_RFX4
Pseudotime bin
Bits
E
TFME MA0886.1_EMX2
Pseudotime bin
Bits

Suppl.Fig.11 Analysis of discretized TFME changes across pseudotime in CBD astrocytes

A Projections of pseudotime steps (left) and the MA0099.2_FOS::JUN enrichment (right) onto the UMAP embedding of Ctrl/CBD astrocytes indicating the respective variable as color code.
B-E Boxplots showing the TFME values of selected TFMs in Ctrl/CBD astrocytes over 5 discrete pseudotime steps. The color code emphasizes the assigned pseudotime bin. Statistical comparison was conducted between each time step > 1 and the first one (*Wilcoxon* rank-sum test) and results expressed as asterisks where *p<.05, **p<.01, ***p<.001 and ****p<.0001. On the right, the position weight matrix is displayed via motif sequence logos. The information content is depicted as bits for every base position of the motif sequence.

Suppl.Fig.12

A

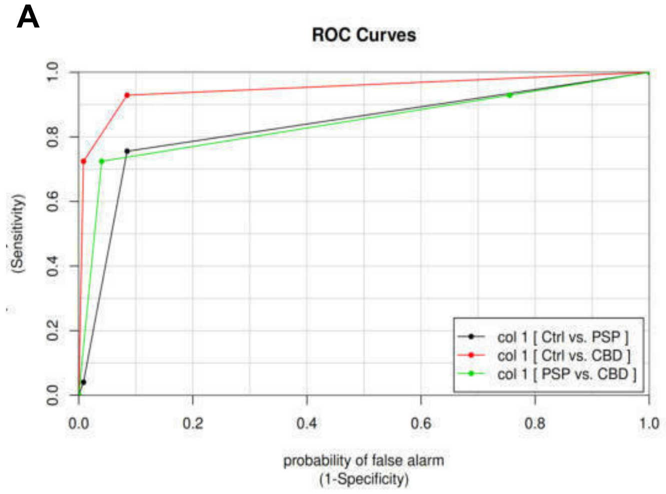

B

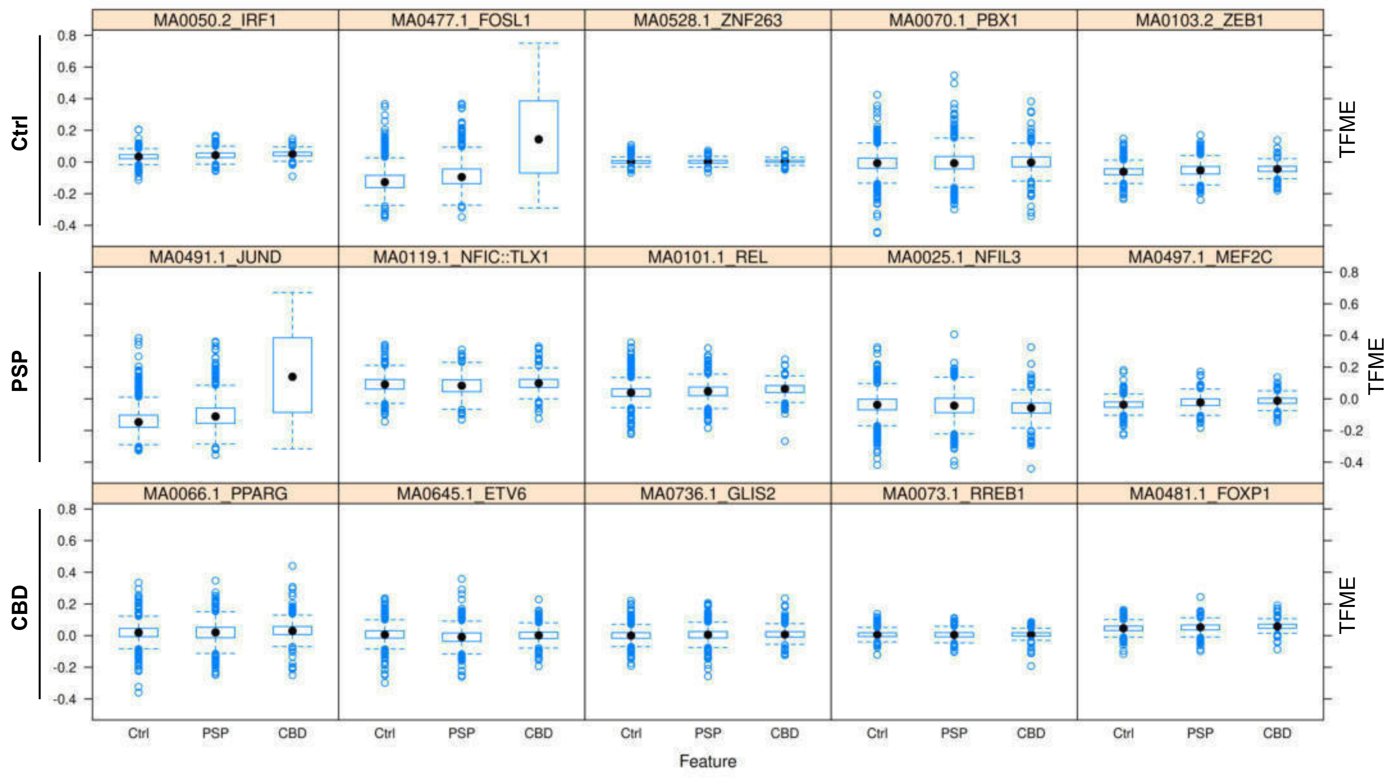

Suppl.Fig.12 XGB model performance and most discriminative features for predicting the astrocyte disease entity

A The sensitivity vs. counter-probability of specificity is drawn as receiver-operator curves (ROC) of the trained XGB model performance on an unseen astrocytic TF matrix subset. Differently colored curves display the three comparisons between Ctrl, PSP, and CBD assigned astrocytes.
B Feature matrix boxplots depicting the TFME distribution of the 5 most important features in the XGB model representation for each group entity (top: Ctrl, middle: CBD, lower: PSP) as extracted from *Lime*. Outliers are depicted as blue circles. The hinges of each box correspond to the 25th and 75th percentiles while medians drawn as black dots. The 1.5-times inter-quartile ranges are shown as black whiskers. The entire range is scaled to values from -0.4 to 0.8 for all features.
Abbreviations: col, color.

Suppl.Fig.13

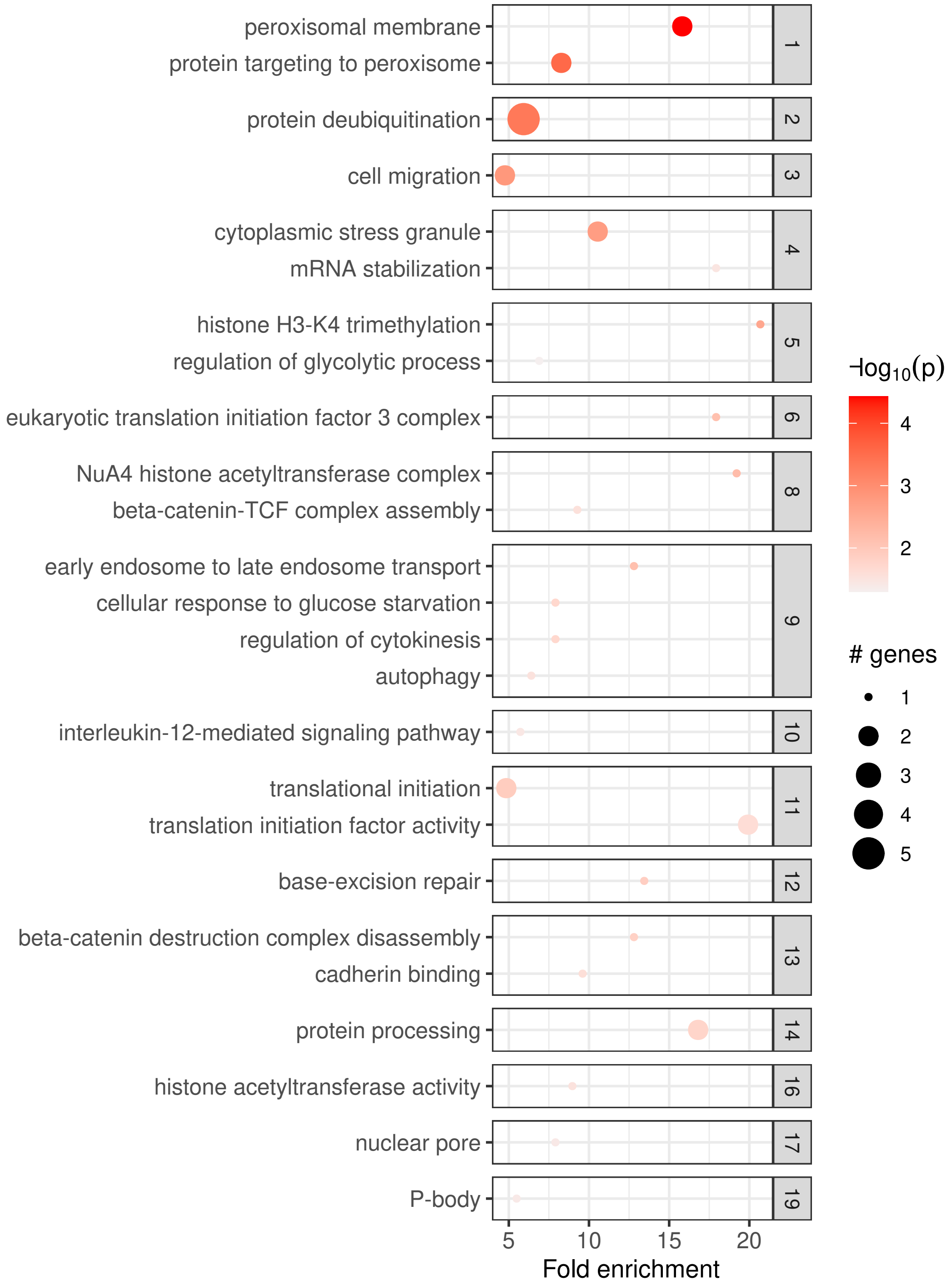
peroxisomal membrane
protein targeting to peroxisome
protein deubiquitination
cell migration
cytoplasmic stress granule
mRNA stabilization
histone H3-K4 trimethylation
regulation of glycolytic process
eukaryotic translation initiation factor 3 complex
NuA4 histone acetyltransferase complex
beta-catenin-TCF complex assembly
early endosome to late endosome transport
cellular response to glucose starvation
regulation of cytokinesis
autophagy
interleukin-12-mediated signaling pathway
translational initiation
translation initiation factor activity
base-excision repair
beta-catenin destruction complex disassembly
cadherin binding
protein processing
histone acetyltransferase activity
nuclear pore
P-body
1
2
3
4
5
6
8
9
10
11
12
13
14
16
17
19
5
10
15
20
Fold enrichment
-log10(p)
4
3
2
genes
1
2
3
4
5

Suppl.Fig.13 GO analysis of TA-associated regulons

Clustered depiction of GO term enrichment in all genes regulated by the top 50 TA-associated regulons. The x-axis differentiates the GO term fold enrichment, the negative decadic logarithm of respective p-values is indicated as color gradient, and the gene set size is expressed as point size.
Abbreviations: # genes, number of genes.

Suppl.Fig.14

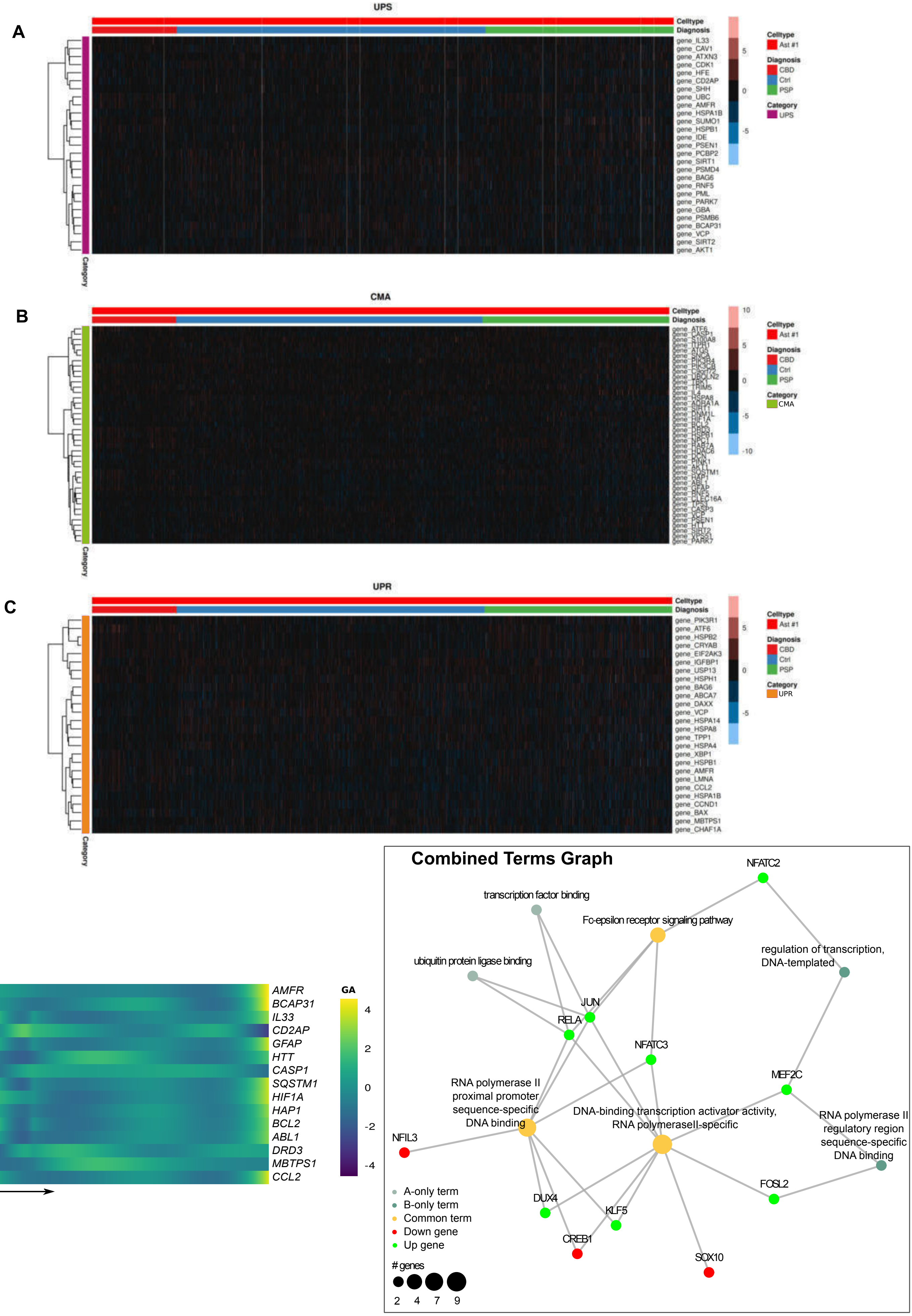
A
UPS
Celltype
Diagnosis
gene_IL33
gene_CAV1
gene_ATXN3
gene_CDK1
gene_HFE
gene_CD2AP
gene_SHH
gene_UBC
gene_AMFR
gene_HSPA1B
gene_SUMO1
gene_HSPB1
gene_IDE
gene_PSEN1
gene_PCBP2
gene_SIRT1
gene_PSMD4
gene_BAG6
gene_RNF5
gene_PML
gene_PARK7
gene_GBA
gene_PSMB6
gene_BCAP31
gene_VCP
gene_SIRT2
gene_AKT1
Category
5
0
-5
Celltype
Ast #1
Diagnosis
CBD
Ctrl
PSP
Category
UPS
B
CMA
Celltype
Diagnosis
Category
10
5
0
-5
-10
Celltype
Ast #1
Diagnosis
CBD
Ctrl
PSP
Category
CMA
C
UPR
Celltype
Diagnosis
gene_PIK3R1
gene_ATF6
gene_HSPB2
gene_CRYAB
gene_EIF2AK3
gene_IGFBP1
gene_USP13
gene_HSPH1
gene_BAG6
gene_ABCA7
gene_DAXX
gene_VCP
gene_HSPA14
gene_HSPA8
gene_TPP1
gene_HSPA4
gene_XBP1
gene_HSPB1
gene_AMFR
gene_LMNA
gene_CCL2
gene_HSPA1B
gene_CCND1
gene_BAX
gene_MBTPS1
gene_CHAF1A
Category
5
0
-5
Celltype
Ast #1
Diagnosis
CBD
Ctrl
PSP
Category
UPR
D
UPS
CMA
UPR
AMFR
BCAP31
IL33
CD2AP
GFAP
HTT
CASP1
SQSTM1
HIF1A
HAP1
BCL2
ABL1
DRD3
MBTPS1
CCL2
GA
4
2
0
-2
-4
Pseudotime
Combined Terms Graph
NFATC2
transcription factor binding
Fc-epsilon receptor signaling pathway
regulation of transcription, DNA-templated
ubiquitin protein ligase binding
JUN
RELA
NFATC3
MEF2C
RNA polymerase II proximal promoter sequence-specific DNA binding
DNA-binding transcription activator activity, RNA polymeraseII-specific
RNA polymerase II regulatory region sequence-specific DNA binding
NFIL3
FOSL2
DUX4
KLF5
CREB1
SOX10
A-only term
B-only term
Common term
Down gene
Up gene
genes
2
4
7
9

Suppl.Fig.14 Assessing the involvement of protein degradation pathways on a system level

A-C Gene accessibility (GA) heatmaps of genes associated with three major protein homeostasis pathways (UPS, **A**; CMA, **B**; UPR, **C**) in astrocytes. Every column corresponds to a single nucleus and every row to a specific gene, while the color gradient indicates the extent of GA from negative (blue) through intermediate (black) to high (red) values. Rows were clustered hierarchically (*Manhattan* distance, *Ward-D2* method) and results indicated as dendrogram on the left. The column order was fixed while the colored bars at the top inform about the cell type and disease diagnosis. Gene names comply with the *Ensembl* identifiers.

D Heatmap displaying the pseudotime changes in GA of tauopathy and degradation pathways-associated, significantly altered genes in the group-wise comparisons (Fig. 6H, main manuscript). The results relate to Ctrl/CBD astrocytes and their pseudotemporal transition exclusively. Gene assignments to these pathways are indicated by colored bars on the left.

E Bubble-connection graph depicting the top 3 co-enriched and top 2 exclusively enriched GO terms of TF signatures in PSP and CBD. The yellow dots represent specific GO terms, while their size equal to the number of term-assigned genes (# genes) that are found in the queried data sets. These genes are displayed as red (co-downregulated), green (co-upregulated), or grey dots (conflicting direction). 'A-only terms' correspond to PSP-related and 'B-only terms' to CBD-related ones.

Abbreviations: CMA, chaperon-mediated autophagy; UPS, ubiquitin-proteasome-system; UPR, unfolded-protein-response.

Suppl.Fig.15

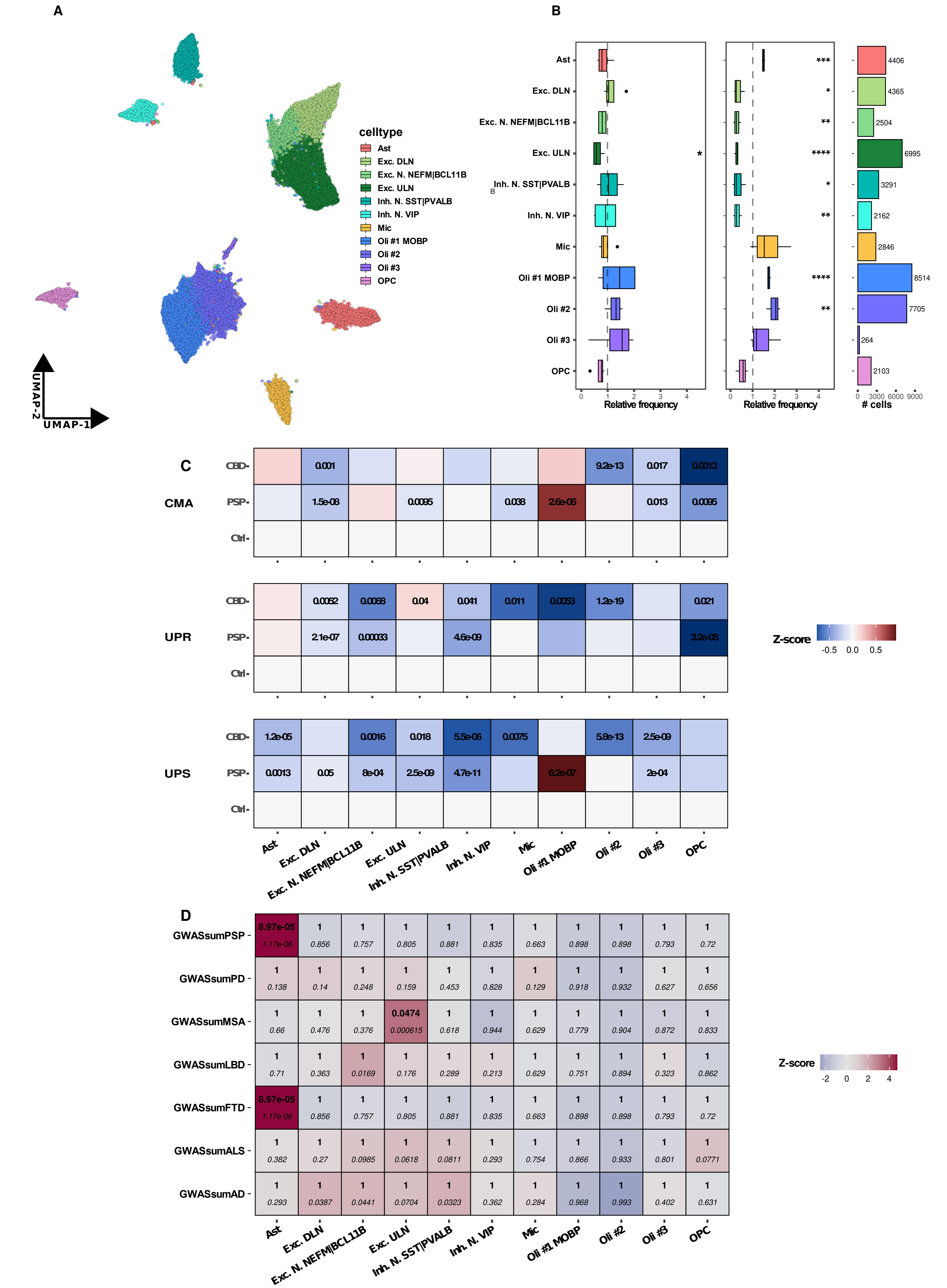
A
B
C
D
celltype
Ast
Exc. DLN
Exc. N. NEFM|BCL11B
Exc. ULN
Inh. N. SST|PVALB
Inh. N. VIP
Mic
Oli #1 MOBP
Oli #2
Oli #3
OPC
UMAP-1
UMAP-2
Relative frequency
cells
4406
4365
2504
6995
3291
2162
2846
8514
7705
264
2103
CMA
UPR
UPS
CBD
PSP
Ctrl
Z-score
GWASsumPSP
GWASsumPD
GWASsumMSA
GWASsumLBD
GWASsumFTD
GWASsumALS
GWASsumAD

Suppl.Fig.15 Re-analysis of the dataset with exclusion of #CBD3 – part 1: dataset characterization

A Plot is analogous to Fig.2a. Projections of cluster-cell type assignments and metadata onto the UMAP embedding. Color coding and labels indicate the cell type or sub-cell type identity where applicable.
B Plot is analogous to Fig.2c. Boxplots of relative cell type frequencies show reductions in excitatory neurons in PSP (left) and in all neuronal populations in CBD. Higher astrocyte *and* oligodendrocyte frequencies can be detected in the remaining CBD samples, when compared to the Ctrls' mean (vertical dashed line). Total numbers of cells (# of cells) are indicated as bar plots on the right.
C.Plot is analogous to Fig.3c. Protein homeostasis-related genes across all cell types differentiated by group entity and degradation pathway show reduced system-level GA in Ast, most neuronal, and Oli populations, while Mic exhibit marked CMA and UPS inductions in PSP. Color coding shows aggregated mean scores of accessibility values at genes that were altered significantly and associated with either the CMA (top), UPR (middle), or UPS degradation systems (bottom). P-values are given for each group vs. Ctrl comparison (2-tailed Welch t-test, if p<=.05).
D Plot is analogous to Fig.3d. Heatmap of genetic risk variant enrichment results in tauopathy cortices resolved by cell type assignments (x-axis) and GWAS data set (y-axis) highlight Ast, which exhibit strong enrichment. Color code indicates z-scores and text inserts depict the uncorrected p-value (*italic*) as well as the BH-corrected p-values (**bold**, *Wilcoxon* rank-sum test).
Abbreviations: CMA, chaperon-mediated autophagy; Exc. DLN, excitatory deep-layer neurons; Exc. ULN, excitatory upper-layer neurons; Inh. N., inhibitory neurons; Mic, microglia; Oli, oligodendrocytes; OPC, oligodendrocytic precursor cells; UPS, ubiquitin-proteasome-system; UPR, unfolded-protein-response.

Suppl.Fig.16

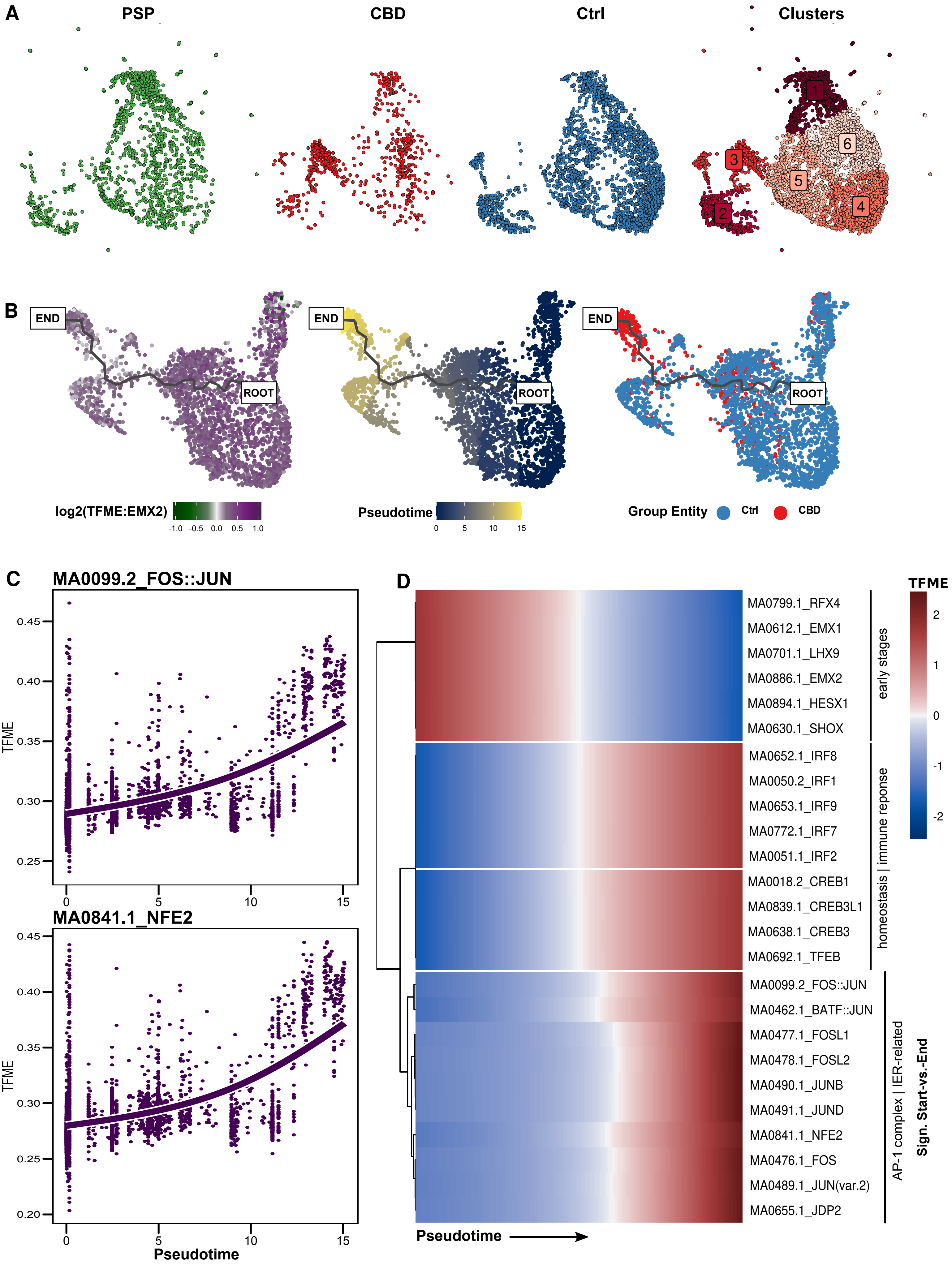
A
PSP
CBD
Ctrl
Clusters
1
2
3
4
5
6
B
END
ROOT
log2(TFME:EMX2)
-1.0 -0.5 0.0 0.5 1.0
Pseudotime
0 5 10 15
Group Entity
Ctrl
CBD
C
MA0099.2_FOS::JUN
TFME
0.25
0.30
0.35
0.40
0.45
0 5 10 15
MA0841.1_NFE2
0.20
Pseudotime
D
TFME
2
1
0
-1
-2
MA0799.1_RFX4
MA0612.1_EMX1
MA0701.1_LHX9
MA0886.1_EMX2
MA0894.1_HESX1
MA0630.1_SHOX
MA0652.1_IRF8
MA0050.2_IRF1
MA0653.1_IRF9
MA0772.1_IRF7
MA0051.1_IRF2
MA0018.2_CREB1
MA0839.1_CREB3L1
MA0638.1_CREB3
MA0692.1_TFEB
MA0099.2_FOS::JUN
MA0462.1_BATF::JUN
MA0477.1_FOSL1
MA0478.1_FOSL2
MA0490.1_JUNB
MA0491.1_JUND
MA0841.1_NFE2
MA0476.1_FOS
MA0489.1_JUN(var.2)
MA0655.1_JDP2
early stages
homeostasis | immune reponse
AP-1 complex | IER-related
Sign. Start-vs.-End
Pseudotime

Suppl.Fig.16 Re-analysis of the dataset with exclusion of #CBD3 – part 2: Pseudotime trajectory of CBD astrocytes

A Plot is analogous to Fig.4a. All *PSP-, CBD-, and Ctrl*-derived astrocytes (excluding #CBD3) re-embedded in UMAP, stratified by group entity (first, second, third panel), and depicted after k-means clustering in a merged UMAP (fourth panel). One cluster (#3) remains specific for CBD astrocytes. Color code indicates group entity or cluster assignments in the first three or the fourth panel, respectively.
B Plot is analogous to Fig.4b-d. Exclusively *CBD- and Ctrl*-derived astrocytes (excluding #CBD3) re-embedded in UMAP. A pseudotime trajectory leads from a non-specific Ast pool towards a CBD-enriched population. Color code indicates log2(EMX2 TFME) (left), pseudotime (mid, dimensionless), or group entity (right). The black line indicates the pseudotemporal trajectory from the *root* towards the *end* cell.
C.Plot is analogous to Fig.4e-f. Generative additive model non-linear fits of TFME values over pseudotime of the FOS-JUN (upper) or NFE2 (lower) motifs indicate parallel increments during the astrocytic transition towards a CBD-state.
D Plot is analogous to Fig.4g. Pseudotime heatmap displaying the TFME values of significantly altered TFMs in the start-vs.-end comparison (*Wald*-testing, *BH*-adjusted $p<.05$), as well as markers of early astrocytic development or immune regulation. TFME of astrocytic early-stage TFs is gradually decreasing, while immunologically relevant and AP-1 complex-related TFs gain in motif enrichment.
Abbreviations: IER, immediate-early response; TFME, transcription factor motif enrichment; Sign, significant.

Suppl.Fig.17

A

Overall Accuracy	0.817
Balanced Accuracy	0.83
F1	0.784
Sensitivity	0.766
Specificity	0.893
Pos Pred Value	0.81
Neg Pred Value	0.9
Kappa	0.681

B

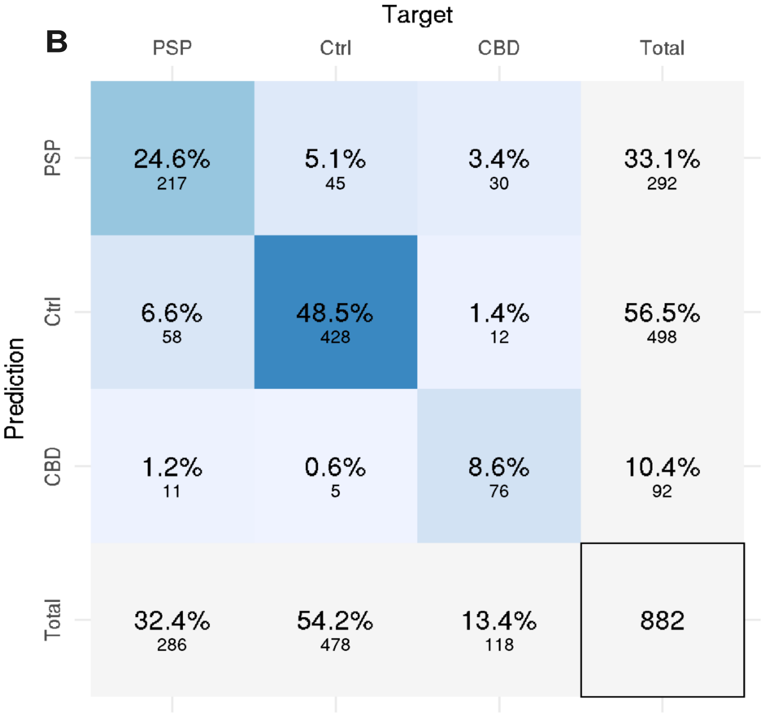

C

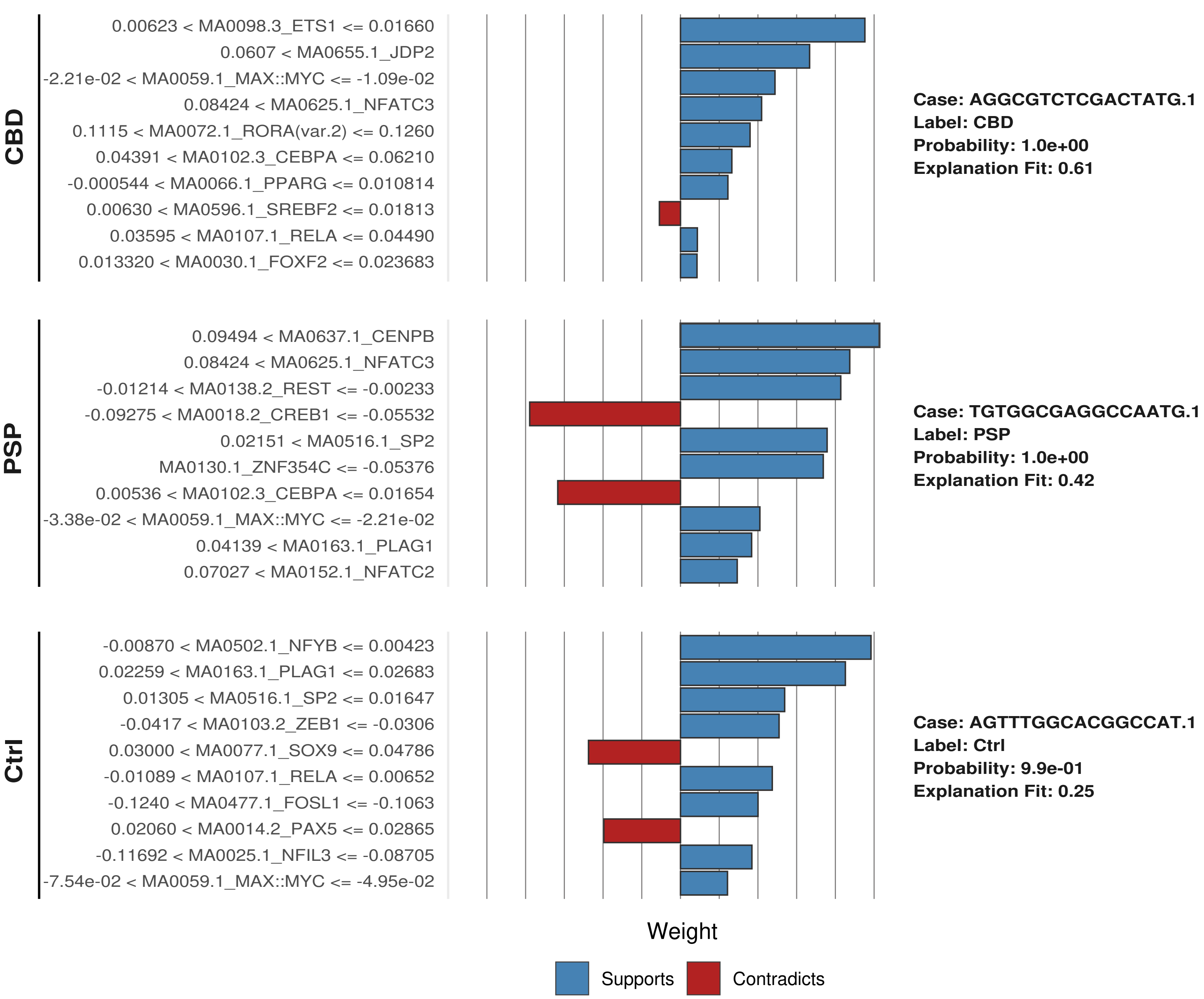

Suppl.Fig.17 Re-analysis of the dataset with exclusion of #CBD3 – part 3: TF networks associated with an astrocytic tauopathy state

A Plot is analogous to Fig.5b. Evaluation parameters of classification performance of the trained XGB model on the 20% test set-split of astrocytic nuclei (excluding #CBD3). Overall, more than 81% of predictions were correct (overall accuracy) and the model performs substantially with a *Cohen* kappa of 68.1%.
B Plot is analogous to Fig.5a. Confusion matrix displaying the intersections of the XGB model's predictions (rows) and the actual labels (columns). Each square contains the percentual proportion (large digits) and total numbers (small digits) of test set samples (=20% astrocytic nuclei excluding #CBD3) with the assigned prediction-label-relation. The sums of each row or column are depicted in the rightmost column or undermost row, respectively. The total sample number (i.e., nuclei of the 20% test set-split) is shown in the downright corner.
C.Plot is analogous to Fig.5d-f. *Lime* feature importance bar diagrams of the most certainly correctly classified barcodes of each group entity. The bar direction and bar color indicate the feature weights (~importance) assigned to the TFM, which are given as y-axis breaks. Note, feature weight was assigned to specific TFME value ranges. Each panel is complemented by the group entity label, the models' calculated probability, and the explanatory model's fit value.
Abbreviations: Neg Pred Value: negative predictive value, Pos Pred Value: positive predictive value; TFM(E), transcription factor motif (enrichment); XGB, extreme gradient boosting tree.

Suppl.Fig.18

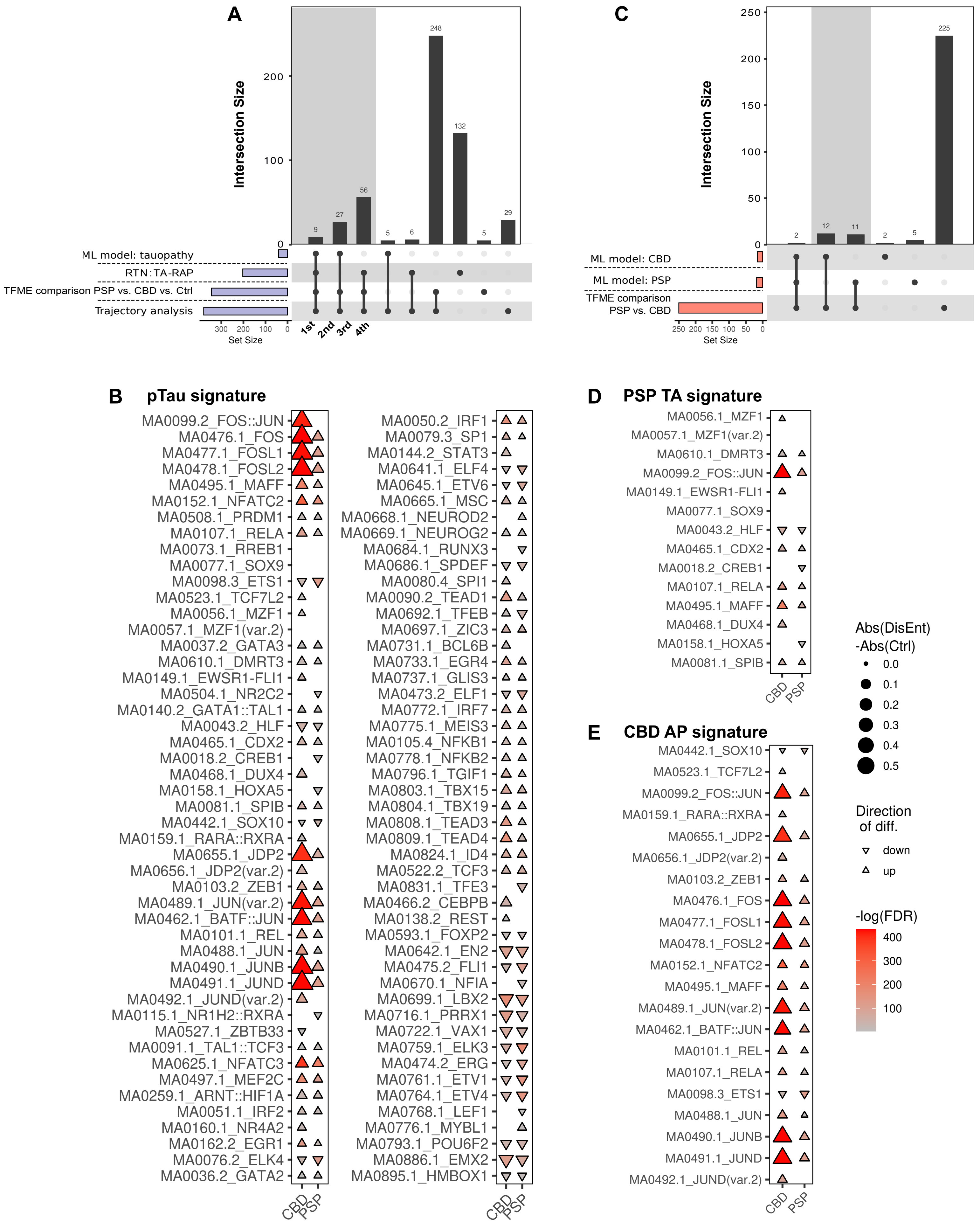
A
Intersection Size
0
100
200
9
27
56
5
6
248
132
5
29
ML model: tauopathy
RTN:TA-RAP
TFME comparison PSP vs. CBD vs. Ctrl
Trajectory analysis
300 200 100 0
Set Size
1st
2nd
3rd
4th
C
Intersection Size
0
50
100
150
200
250
2
12
11
2
5
225
ML model: CBD
ML model: PSP
TFME comparison
PSP vs. CBD
250 200 150 100 50 0
Set Size
B
pTau signature
MA0099.2_FOS::JUN
MA0476.1_FOS
MA0477.1_FOSL1
MA0478.1_FOSL2
MA0495.1_MAFF
MA0152.1_NFATC2
MA0508.1_PRDM1
MA0107.1_RELA
MA0073.1_RREB1
MA0077.1_SOX9
MA0098.3_ETS1
MA0523.1_TCF7L2
MA0056.1_MZF1
MA0057.1_MZF1(var.2)
MA0037.2_GATA3
MA0610.1_DMRT3
MA0149.1_EWSR1-FLI1
MA0504.1_NR2C2
MA0140.2_GATA1::TAL1
MA0043.2_HLF
MA0465.1_CDX2
MA0018.2_CREB1
MA0468.1_DUX4
MA0158.1_HOXA5
MA0081.1_SPIB
MA0442.1_SOX10
MA0159.1_RARA::RXRA
MA0655.1_JDP2
MA0656.1_JDP2(var.2)
MA0103.2_ZEB1
MA0489.1_JUN(var.2)
MA0462.1_BATF::JUN
MA0101.1_REL
MA0488.1_JUN
MA0490.1_JUNB
MA0491.1_JUND
MA0492.1_JUND(var.2)
MA0115.1_NR1H2::RXRA
MA0527.1_ZBTB33
MA0091.1_TAL1::TCF3
MA0625.1_NFATC3
MA0497.1_MEF2C
MA0259.1_ARNT::HIF1A
MA0051.1_IRF2
MA0160.1_NR4A2
MA0162.2_EGR1
MA0076.2_ELK4
MA0036.2_GATA2
CBD
PSP
MA0050.2_IRF1
MA0079.3_SP1
MA0144.2_STAT3
MA0641.1_ELF4
MA0645.1_ETV6
MA0665.1_MSC
MA0668.1_NEUROD2
MA0669.1_NEUROG2
MA0684.1_RUNX3
MA0686.1_SPDEF
MA0080.4_SPI1
MA0090.2_TEAD1
MA0692.1_TFEB
MA0697.1_ZIC3
MA0731.1_BCL6B
MA0733.1_EGR4
MA0737.1_GLIS3
MA0473.2_ELF1
MA0772.1_IRF7
MA0775.1_MEIS3
MA0105.4_NFKB1
MA0778.1_NFKB2
MA0796.1_TGIF1
MA0803.1_TBX15
MA0804.1_TBX19
MA0808.1_TEAD3
MA0809.1_TEAD4
MA0824.1_ID4
MA0522.2_TCF3
MA0831.1_TFE3
MA0466.2_CEBPB
MA0138.2_REST
MA0593.1_FOXP2
MA0642.1_EN2
MA0475.2_FLI1
MA0670.1_NFIA
MA0699.1_LBX2
MA0716.1_PRRX1
MA0722.1_VAX1
MA0759.1_ELK3
MA0474.2_ERG
MA0761.1_ETV1
MA0764.1_ETV4
MA0768.1_LEF1
MA0776.1_MYBL1
MA0793.1_POU6F2
MA0886.1_EMX2
MA0895.1_HMBOX1
CBD
PSP
D
PSP TA signature
MA0056.1_MZF1
MA0057.1_MZF1(var.2)
MA0610.1_DMRT3
MA0099.2_FOS::JUN
MA0149.1_EWSR1-FLI1
MA0077.1_SOX9
MA0043.2_HLF
MA0465.1_CDX2
MA0018.2_CREB1
MA0107.1_RELA
MA0495.1_MAFF
MA0468.1_DUX4
MA0158.1_HOXA5
MA0081.1_SPIB
CBD
PSP
E
CBD AP signature
MA0442.1_SOX10
MA0523.1_TCF7L2
MA0099.2_FOS::JUN
MA0159.1_RARA::RXRA
MA0655.1_JDP2
MA0656.1_JDP2(var.2)
MA0103.2_ZEB1
MA0476.1_FOS
MA0477.1_FOSL1
MA0478.1_FOSL2
MA0152.1_NFATC2
MA0495.1_MAFF
MA0489.1_JUN(var.2)
MA0462.1_BATF::JUN
MA0101.1_REL
MA0107.1_RELA
MA0098.3_ETS1
MA0488.1_JUN
MA0490.1_JUNB
MA0491.1_JUND
MA0492.1_JUND(var.2)
CBD
PSP
Abs(DisEnt)
-Abs(Ctrl)
0.0
0.1
0.2
0.3
0.4
0.5
Direction
of diff.
down
up
-log(FDR)
400
300
200
100

Suppl.Fig.18 Re-analysis of the dataset with exclusion of #CBD3 – part 4: Astrocytic tauopathy signatures

A Plot is analogous to Fig.6a. Upset plot illustrating TFs useful in distinguishing PSP/CBD from Ctrl astrocytes (when excluding #CBD3) that resulted from (i) group-wise TFME comparisons in the snATAC-seq data set ('TFME' comparison'), (ii) the pseudotime trajectory analysis in the snATAC-seq data set ('Trajectory analysis'), (iii) the TA-related regulon activity profile in the bulkRNA-seq data set ('RTN: TA-RAP'), and (iv) interpreting the XGB classification model ('ML model: tauopathy'). Set sizes are indicated as blue bars, while the intersection logic is shown as vertical lines and dots. Column heights depict the extent of intersection for the given sets. The first three intersections were assigned a hierarchy of importance in defining the primary tauopathy context.
B Plot is analogous to Fig.6b. Triangle plot indicating significance, absolute extent, and direction of TFME changes in **pTau signature TFs** in tauopathy-assigned astrocytes. The triangle tips point towards the direction of change while the size represents the absolute difference from the TFME reference in Ctrls. Fill shading displays the negative decadic logarithm of the BH-corrected p-values from pair-wise *Wilcoxon* rank-sum tests. Empty coordinates inform about non-significant comparisons in the test set.
C. Plot is analogous to Fig.6c. Upset plot to identify TFs useful in differentiating CBD from PSP astrocytes (when excluding #CBD3). The single sets resulted (i) from the most important TFs for PSP or CBD prediction according to the XGB model explainer ('ML model: PSP', 'ML model: CBD') and (ii) from pairwise statistical TFME comparisons between PSP and CBD astrocytes ('TFME comparisons'). The general plot structure equates to A.
D-E Plots are analogous to Fig.6d-e. Triangle plot indicating significance, absolute extent, and direction of TFME changes in **PSP (D), and CBD (E) signature TFs** in tauopathy-assigned astrocytes (when excluding #CBD3). The general plot structure equates to B.
Abbreviations: Abs.diff., absolute difference; AP, astrocytic plaque; Dis.Ent., disease entity; FDR, false discovery rate; ML, machine learning; TA, tufted astrocyte; TFM(E), transcription factor motif (enrichment).

Suppl.Fig.19

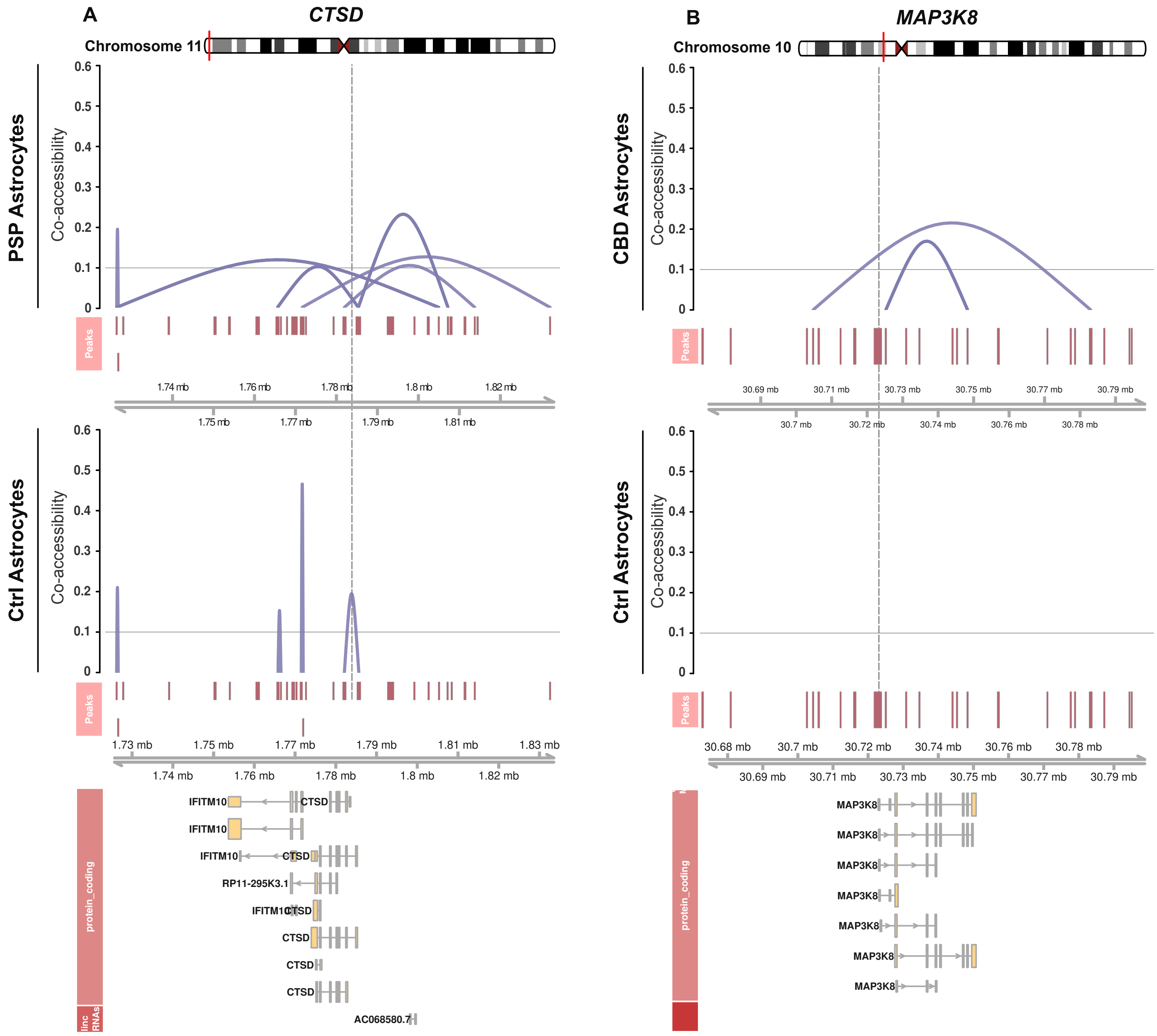
A
CTSD
Chromosome 11
PSP Astrocytes
Co-accessibility
Peaks
Ctrl Astrocytes
1.73 mb
1.74 mb
1.75 mb
1.76 mb
1.77 mb
1.78 mb
1.79 mb
1.8 mb
1.81 mb
1.82 mb
1.83 mb
protein_coding
linc RNAs
IFITM10
CTSD
RP11-295K3.1
AC068580.7
B
MAP3K8
Chromosome 10
CBD Astrocytes
30.68 mb
30.69 mb
30.7 mb
30.71 mb
30.72 mb
30.73 mb
30.74 mb
30.75 mb
30.76 mb
30.77 mb
30.78 mb
30.79 mb

Suppl.Fig.19 Co-accessibility at JUNB and TFEB target genes in CBD & PSP astrocytes.

A-C Co-accessibility plots at genomic loci including genes that are correlated with TFEB and/or JUNB motif enrichment (**A** *CTSD*, **B** *MAP3K8*) in astrocytes. Chromosomal location is depicted at the top of each panel, followed by *Cicero's* co-accessibility links in PSP/CBD (upper) and Ctrl (middle) astrocytes. The y-axes correspond to the extent of co-accessibility, while the value 0.1 is chosen as cut-off to reduce depiction of technical or biological noise. Annotations for this genomic frame (gene location +/- 10e4 bp) including protein coding gene isoforms as well as lincRNAs are given in the bottom panel. The distribution of summed ATAC peaks are given beneath each co-accessibility plot.
Abbreviations: lincRNAs, long intervening/intergenic noncoding RNAs.
