# Supplementary material for: Single-nucleus chromatin accessibility profiling highlights distinct astrocyte signatures in progressive supranuclear palsy and corticobasal degeneration
Source: Acta Neuropathol. 2022 Aug 17;144(4):615–35. doi: 10.1007/s00401-022-02483-8 (PMC9468099; doi:10.1007/s00401-022-02483-8)
Supplement: Supplementary file 2 — (PDF 370 KB) [file 401_2022_2483_MOESM2_ESM.pdf]

Single-Nucleus Chromatin Accessibility Profiling Highlights Distinct Astrocyte Signatures in Progressive Supranuclear Palsy and Corticobasal Degeneration

Nils Briel, Viktoria C Ruf, Katrin Pratsch, Sigrun Roeber, Jeannine Widmann, Janina Mielke, Mario M Dorostkar, Otto Windl, Thomas Arzberger, Jochen Herms*, Felix L Struebing*

* These authors contributed equally

- Supplemental Methods -

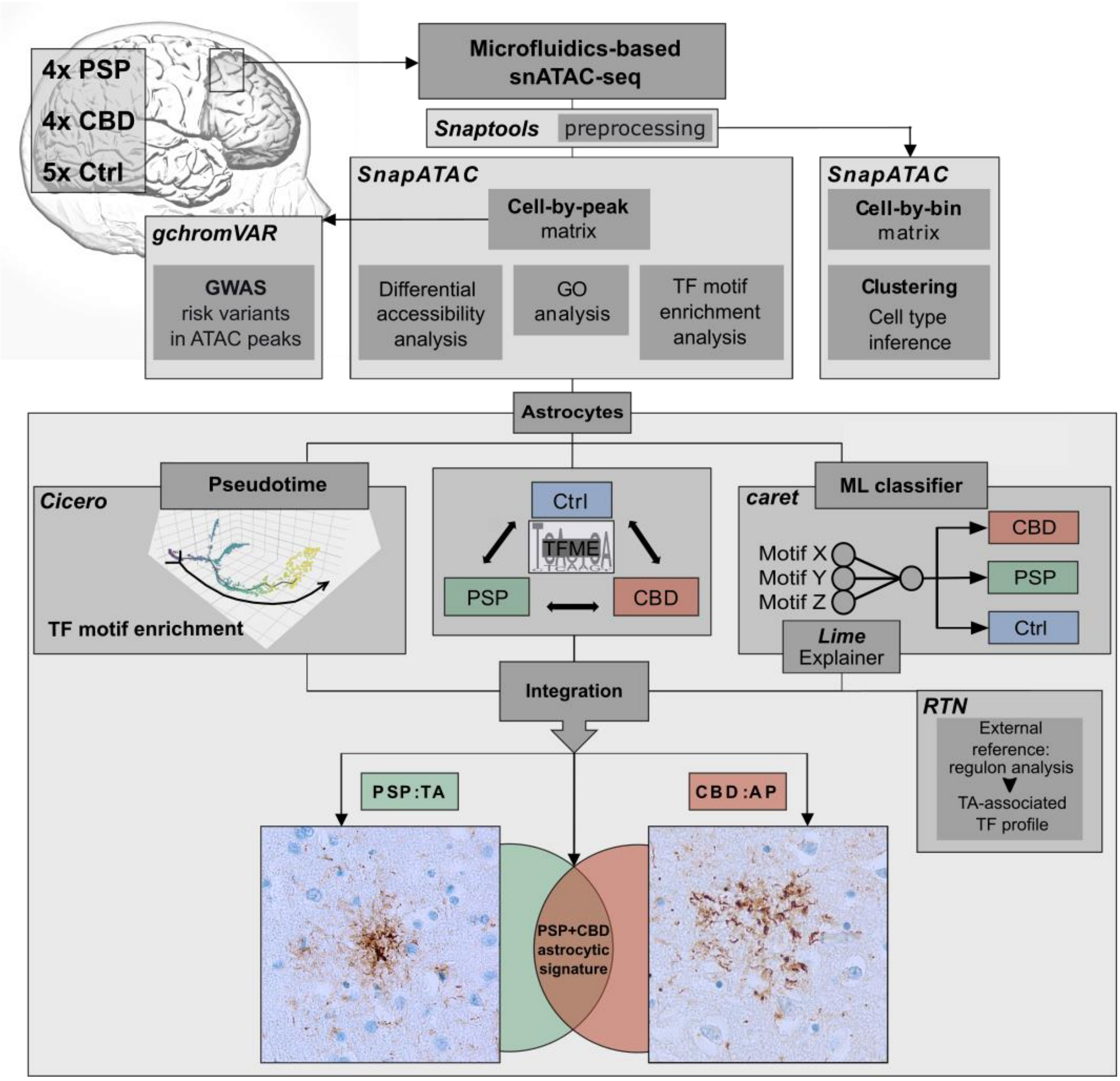

Supplemental Methods Fig.1 Comprehensive bioinformatical analysis flow diagram.

SnATAC-sequencing was applied to cryopreserved frontal cortex samples from deceased PSP, CBD, and Ctrl individuals. Raw sequencing reads were preprocessed with *Snaptools* and *SnapATAC*. The resulting matrices were then used (i) for graph-based clustering and cell type inference (using a binned genome), and (ii) for peak-calling, GO, and TF-motif analysis (using the peak matrix). Furthermore, the peak matrix was subjected to GWAS risk variant-association with cell types.

Downstream, only the astrocytic cluster was investigated (boxed lower part) to find altered TF motif enrichment (TFME) in pairwise comparisons (mid panel), TFME changes along pseudotime trajectories (left), and to train an ML-based disease classifier (right). Finally, significant results from all these three branches were integrated, and refined by a TA-associated TF profile extracted from an external dataset, to define either a general astrocytic tauopathy TF signature, or entity-specific astrocytic TF signatures. These signatures were presumed to mirror the neuropathological context of characteristic pTau inclusions in astrocytes, namely TA in PSP and AP in CBD. Names of algorithms employed in this analysis are given in ***bold italic*** in the upper left corner of each panel.

Abbreviations: AP, astrocytic plaque; GO, gene ontology; GWAS, genome wide association studies; ML, machine learning; pTau; hyperphosphorylated Tau; RTN, Reconstruction of Transcriptional Networks; TA, tufted astrocyte; TFME, transcription factor motif enrichment.

Analysis of snATAC-seq data

snATAC-seq data pre-processing, peak calling, and peak matrix construction

Raw sequencing reads in *.bcl-format were de-multiplexed into *.fastq using the 10x Genomics™ *cellranger-atac-1.2.0* software with cellranger-atac mkfastq. Subsequently, cellranger-atac count was executed on single-sample fastq-files to generate general QC metrics and sequence alignment maps in *.bam format according to the reference data (10x Genomics-indexed genome hg19/GRCh37.87 (hg19), https://cf.10xgenomics.com/supp/cell-atac/refdata-cellranger-atac-hg19-1.2.0.tar.gz). The cellranger-atac output allowed a primary QC of each sample regarding sequencing metrics, included cells, insert sizes, targeting metrics, and library complexity.

Next, these quality assessment values were set to define exclusion criteria for single barcode (assigned cell)-fragment vectors, so that only those barcoded cells remained, whose fragments reached a mapping quality score (MAPQ) of at least 30, did not exceed the length of 1000 bp, had a coverage of at least 500, and which satisfied correctly paired ends on the basis of alignment flags. Incorporating these QC parameters, the snaptools snap-pre function [1] generated a *Single-Nucleus Accessibility Profiles* file (.snap). After generating a cell-by-bin matrix with snaptools snap-add-bmat (window size: 1000 bp) within each snap-file, downstream analysis was continued in an RStudio Server/R3.6 environment by importing and instantiating the <sample>.snap objects. A final QC measure followed to restrict the inclusion in terms of unique fragment counts (3 <= UMI <= 6) and fragments/promoter ratio (.1 <= ratio <= .7).

SnapATAC: quality control, clustering, and cell type identification

We utilized the R package *SnapATAC* [2] to perform matrix binarization, clustering, differential accessibility, GO, and TFM analysis on the preprocessed snATAC-seq data. Single sample datasets were preprocessing before merging into a single, all samples comprising snap-file, followed by downstream matrix manipulation. Therefore, the entire genome was binned into 1000 bp-large segments and binary normalized, which had been shown to biologically and computationally improve clustering performance [3]. Fragments overlapping with regions present in the ENCODE blacklist [4] or the mitochondrial chromosome, or which represented the top 5% bins at transcription start sites were excluded, since those could systematically compromise subsequent steps. Dimensionality reduction and feature extraction was conducted by applying the diffusion maps algorithm in combination with *Nyström* density-based sampling (because of large sample sizes). Significant components were determined *ad hoc* and set as eigen dimensions in k-nearest

neighbor clustering (kNN, k = 15, eigen dimensions = 1 to 25, *Euclidean* distance, resolution = 1). This graph-based approach was guided by the *Leiden* algorithm to find optimally connected communities/clusters [5]. The resulting cluster number showed a robust gap statistic of 0.943, when applied to a subset of 1,000 barcodes/cells and the top 3 quartiles of accessibility bins in a *post hoc* cluster validation (Suppl.Fig.1). This parameter describes the deviation of intra-cluster variation at different cluster sizes k from a randomly distributed reference data set and should be maximized. Subset size was determined by visual cluster purity, choosing the minimum cell number that resulted in overlapping cluster assignments (1,000 cells, Suppl.Fig.1). Downscaling was necessary due to the algorithm's computing capacity. Barcodes were then embedded in two-dimensional (2D) space using uniform manifold approximation and projection (UMAP). Batch effects were levelled out by *harmony* [6] accounting for the assigned case identifiers (IDs) in the first 25 eigen dimensions. Thus, main technical confounders showed no specific enrichment within single clusters (Suppl.Fig.2A&B). In a sample-specific evaluation, sequencing, and biological covariates (e.g., unmapped reads, duplicate likelihood, low MAPQ, and promoter ratio, mitochondrial reads, blacklist region fragments) showed high correlations, but not with epidemiological covariates (age at death, PMI) (Suppl.Fig.2D).

GA scores were calculated in *SnapATAC* and utilized to identify cluster-wise cell type identities. Reference marker genes for brain cell types were included from McKenzie et al. [7] and Lake et al. [8], and are provided with Suppl.Data01,T01. Visual inspection of projected GA scores on cells in UMAP guided cell type assignments (Suppl.Fig.3&4).

SnapATAC: Peak calling, GO, and TFM analysis

For peak calling, reads from cells of the same cluster (n > 100 cells) were aggregated first. Then, peaks were extracted for each cluster individually with MACS2, given the options `--nomodel --shift 75 --ext 150 --qval 5e-2 -B --SPMR`. Considering this cluster-wise peak-matrix as reference, the cell-by-peak matrix (*pmat*) was deduced from the merged peaks and the binarized matrix (*bmat*).

To identify differentially accessible peaks among clusters, a kNN-based approach was followed, which accounted for a reference background to compare with in the local graph environment. Using *SnapATAC's* implementation of *edgeR's* (v3.18.1) differential analysis scoring, *Benjamini-Hochberg* (*BH*)-corrected p-values were read out for a biological coefficient of variation of 0.25 to identify differentially accessible regions (DARs). DARs in smaller clusters (n <= 100 cells) were detected for the top 2,000 peaks in a rank-based enrichment metric. Next, *chromVAR-motif* [9] was used to compute TFME from the peaks-

input in the *pmat*, which resulted in a motif matrix (*mmat*; ref. genome hg19, minimum cells per peak = 10). With this approach, we found a total of 373,957 peaks and 386 TFMs.

The *rGREAT* package [10] was applied on the DARs of each cluster to obtain GO term enrichment for molecular function (MF), biological process (BP), and cellular compartment (CC). *BH*-corrected p-value statements and corresponding binomial enrichment values were reported, as indicated in the figures.

Astrocyte sub-clusters: Co-accessibility, pseudotime inference and TFME tracing

To regress peak co-accessibility and to delineate single-nucleus accessibility pseudotime trajectories in astrocytes, we deployed the updated *Cicero* [11,12] version developed with *Monocle3*.

First, a *CellDataSet (cds)* was created given the barcode vector and *pmat* from the astrocytes snap object. Then we followed single steps as described in the version-specific vignette of *Cicero* [12]. Briefly, we preprocessed the *cds* using principal component analysis (PCA) to obtain a reduced dimensionality of 50 (default) and regressed out batch effects with `align_cds`, taking the case IDs as covariate. Cells were embedded in 2D with UMAP and astrocytic subclusters detected with k-means clustering.

For single-cell trajectory construction, functions from *Cicero/Monocle3* to learn a trajectory graph was applied to a re-processed *cds* in UMAP for each disease entity separately, but while including Ctrl astrocytes as biological reference and origin of the trajectory. Root ('origin') cells were defined as the population with the highest TFME for EMX2, a developmentally early, astrocytic TF [13]. Epigenetic changes of TFME and GA along pseudotime were modeled separately using *tradeSeq*'s [14] `fitGAM` function for each disease-specific trajectory. Differences regarding start to end feature values and lineage associations were statistically tested with *Wald*-test-based functions.

In order to discretize pseudotime steps, as depicted in Suppl.Fig.11, all cells were partitioned to one of 5 equally sized pseudotime bins. TFME scores were pairwise compared across those bins, where the first one was set as reference (*Wilcoxon* rank-sum test).

Astrocytes sub-clusters: GO analysis and TFME comparisons

GO assessment was applied on the UMAP embedding of astrocytes obtained from the previous *Cicero*-based dimensionality reduction. GO analysis of TF proteins was conducted with *pathfindR*. Binomial testing enrichment and p-values with *BH*-correction were reported. The same tool was used for analyzing relations of terms and proteins in the

bubble-connections graphs. To identify significant differences of active TFs between the three disease groups, pairwise comparisons of TFME medians was conducted, using a *Wilcoxon* rank-sum test and the *BH* method for multiple hypothesis correction.

Quantification of protein degradation changes or microglial activation was enabled by the *amiGO2* database (http://amigo.geneontology.org/amigo/search/bioentity) filtered for the terms 'chaperon-mediated autophagy' (CMA), ubiquitin-proteasome-system (UPS), and unfolded-protein-response (UPR) or 'microglial cell activation' in *Homo sapiens*. Gene lists were downloaded March 7th, or June 10th, 2021, respectively, and subjected to *SnapATAC's* GA calculation. Then, disease- and cell type-wise mean GA values (of genes associated with one of these gene lists) were calculated for statistical comparison (*Welch* t-test).

Modeling TF states, analyzing branch intersections and triangle plots

To train machine learning classifiers, the astrocyte TFME matrix was first split into a train (80% of cells) and test (20% of cells) set. Then a decision tree-based modeling algorithm (extreme gradient boosting tree, XGB) was fit to the train set with a 3 times repeated 10-fold cross validation control strategy in *caret* [15]. Predictive performance was measured on the test set in terms of overall accuracy and *Cohen's kappa* as chance-corrected agreement measure in categorical problems [16]. The ML model explanation framework *Lime* [17] was used to learn an interpretable representation of the complex XGB by fitting multiple local linear models to the permuted predictions of the original model. Extracted feature weights from these simpler models were considered to describe the importance of each feature, namely TFMs, in favoring one of the group entities.

To determine the intersections of TFs associated with either the trajectory changes, a disease group in the triangular comparison, the model's feature importance, or with the appearance of TAs in PSP, upset plots were constructed with *UpSetR* [18].

Triangle plots were considered to extend volcano plots in differentiating a grouping identity against feature scores. In this approach, two columns (C, C_{ref}) of the same feature (f) were stratified by disease entity (*i)* and their medians statistically evaluated against each other, where C_{ref} was the median of the respective Ctrl subset (*Wilcoxon* rank-sum test, *BH* correction). Then the extent of absolute difference of medians between $C_{i,f}$ and $C_{ref\ i,f}$ was depicted as symbol size and the respective negative decadic logarithm of p-values was indicated as color code. The tips of the triangles finally show the direction of value change in the comparison of interest.

gchromVAR: risk variant enrichment analysis in snATAC-seq data

We used *gchromVAR* [19,20] to asses single nucleus-resolved GWAS risk variant enrichment in the chromatin accessibility data set comprising all identified cell types and following the *gchromVAR_vignette.Rmd*. GWAS summary statistics for PSP (Orphanet_683), CBD (Orphanet_278), FTD (Orphanet_282), AD (EFO_0000249), PD (EFO_0002508), MSA (EFO_1001050), LBD (EFO_0006792), and ALS (EFO_0000253) were downloaded from the EBI-GWAS catalogue [21] January 7th, 2021. We used a *pmat* derivate depicting cluster- or cell type-wise peak sums from the previously assigned snap object and discarded empty or unmapped peak columns. Together with the genomic peak description table, a *RangedSummarizedExperiment* object was created. Then, GC bias was added, a measure introduced by the developers of *chromVAR* to account for background properties in the hg19 reference genome. By finding overlaps of the peak distributions in the dataset with risk variant annotations in the summary statistics, *gchromVAR* implements 'weighted deviations' as z-scores to evaluate the extent of cell type-specific enrichment and provides *Bonferroni*-corrected p-values.

Analysis of transcriptional regulatory networks in PSP

Processed phenotype-gene expression regression data from bulkRNA-seq in temporal cortices (TCX) of PSP brains [22] were downloaded from https://link.springer.com/article/10.1007%2Fs00401-018-1900-5#SupplementaryMaterial (Table 04, Excel-file). Subject covariates with the accession doi:10.7303/syn3817650.5, as well as normalized gene-mapped read counts with the accession doi:10.7303/syn3607513.1 (MayoRNA-seq-Pilot PSP TCX) and doi:10.7303/syn4650265.4 (MayoRNA-seq PSP TCX) were downloaded from the AMP-AD knowledge portal. During pre-processing of the primary data, 25,937 single transcripts from the MayoRNAseq Study [23] could be assigned to a total of 14,056 annotated *Ensembl* gene IDs using the hg19 reference genome. Based on a consensus list of 1,590 human TFs [24], 1,097 TFs could be identified by their *Ensembl* IDs in the underlying expression data set. For network inference, only the PSP cohorts comprising 176 samples were used.

For the *Reconstruction of Transcriptional Regulatory Networks*, input parameters were defined as follows: a named normalized gene expression matrix (*gexp*, n=176 PSP cases), a named character vector with gene identification codes of all human TFs [24], and a matrix with annotations to all matched gene identification codes (Illumina_ID; Ensemble Gene_ID, hg19 H. sapiens, v86; 'Symbol'). The mutual information, a weighting of the interaction of each TF with all its possible target genes, was calculated from the *gexp*. After permutation

(n = 1000, p-cut-off = 3.21^{-7}) and bootstrapping, only robust regulon edges (corresponding to a TF-target gene connection) were retained. The established transcriptional network (*tnet*) comprised regulons and their binary inner single connection weighting (positive vs. negative).

To assess regulon associations with phenotypic hallmarks in PSP, a numerical vector was included with gene-specific coefficients resulting from *Pearson* correlation between expression and neuropathological latent trait residual values representing the semiquantitative TA levels of PSP brains [22]. Using gene-set enrichment analysis (GSEA) with the *Pearson* coefficient and significant differentially expressed genes (DEG; adj.p-value <= .05), we obtained an enrichment score that reflected the accumulation of phenotype-attributed DEGs in the inferred regulons. Resulting phenotype-associated regulons were filtered for their statistical significance in the comparison of regulon activities between PSP and Ctrl samples from the Allen *et al.* data set. Only those regulons with a *BH*-corrected p <=.05 in GSEA-1T and with a *Bonferroni*-corrected p<.05 in the PSP vs. Ctrl comparison of regulon activities were considered in downstream analysis parts.

References

1. GitHub - r3fang/SnapTools: A module for working with snap files in Python [Internet]. [cited 2021 Mar 14]. Available from: https://github.com/r3fang/SnapTools

2. Fang R, Preissl S, Li Y, Hou X, Lucero J, Wang X, et al. Comprehensive analysis of single cell ATAC-seq data with SnapATAC. Nat Commun. 2021 Dec 1;12(1):1–15.

3. Chen H, Lareau C, Andreani T, Vinyard ME, Garcia SP, Clement K, et al. Assessment of computational methods for the analysis of single-cell ATAC-seq data. 2019;

4. Amemiya HM, Kundaje A, Boyle AP. The ENCODE Blacklist: Identification of Problematic Regions of the Genome. Sci Rep. 2019 Dec 1;9(1):1–5.

5. Traag VA, Waltman L, van Eck NJ. From Louvain to Leiden: guaranteeing well-connected communities. Sci Rep. 2019 Dec 1;9(1):1–12.

6. Korsunsky I, Millard N, Fan J, Slowikowski K, Zhang F, Wei K, et al. Fast, sensitive and accurate integration of single-cell data with Harmony. Nat Methods. 2019 Dec 1;16(12):1289–96.

7. McKenzie AT, Wang M, Hauberg ME, Fullard JF, Kozlenkov A, Keenan A, et al. Brain Cell Type Specific Gene Expression and Co-expression Network Architectures. Sci Rep. 2018 Dec 11;8(1):8868.

8. Lake BB, Ai R, Kaeser GE, Salathia NS, Yung YC, Liu R, et al. Neuronal subtypes and diversity revealed by single-nucleus RNA sequencing of the human brain. Science. 2016 Jun 24;352(6293):1586–90.

9. Schep AN, Wu B, Buenrostro JD, Greenleaf WJ. ChromVAR: Inferring transcription-factor-associated accessibility from single-cell epigenomic data. Nat Methods. 2017 Oct 1;14(10):975–8.

10. Bioconductor - rGREAT [Internet]. [cited 2021 Jan 19]. Available from: http://bioconductor.org/packages/release/bioc/html/rGREAT.html

11. Pliner HA, Packer JS, McFaline-Figueroa JL, Cusanovich DA, Daza RM, Aghamirzaie D, et al. Cicero Predicts cis-Regulatory DNA Interactions from Single-Cell Chromatin Accessibility Data. Mol Cell. 2018 Sep 6;71(5):858-871.e8.

12. Pilner H. Package “cicero.” 2020.

13. Tiwari N, Pataskar A, Pé S, Ló Pez-Mascaraque L, Tiwari VK, Correspondence BB. Stage-Specific Transcription Factors Drive Astrogliogenesis by Remodeling Gene Regulatory Landscapes. Stem Cell. 2018;23:557-571.e8.

14. Van den Berge K, Roux de Bézieux H, Street K, Saelens W, Cannoodt R, Saeys Y, et al. Trajectory-based differential expression analysis for single-cell sequencing data. Nat Commun. 2020;11(1):1–13.

15. Kuhn M. Journal of Statistical Software Building Predictive Models in R Using the caret Package. 2008.

16. Cohen J. A Coefficient of Agreement for Nominal Scales. Educ Psychol Meas. 1960 Jul 2;20(1):37–46.

17. Ribeiro MT, Singh S, Guestrin C. “Why should i trust you?” Explaining the predictions of any classifier. In: Proceedings of the ACM SIGKDD International Conference on Knowledge Discovery and Data Mining. Association for Computing Machinery; 2016. p. 1135–44.

18. Conway JR, Lex A, Gehlenborg N. UpSetR: an R package for the visualization of intersecting sets and their properties. Hancock J, editor. Bioinformatics. 2017 Sep 15;33(18):2938–40.

19. Ulirsch JC, Lareau CA, Bao EL, Ludwig LS, Guo MH, Benner C, et al. Interrogation of human hematopoiesis at single-cell and single-variant resolution. Nat Genet. 2019 Apr 11;51(4):683–93.

20. caleblareau/gchromVAR: Cell type specific enrichments using finemapped variants and quantitative epigenetic data [Internet]. [cited 2020 Apr 3]. Available from: https://github.com/caleblareau/gchromVAR

21. MacArthur J, Bowler E, Cerezo M, Gil L, Hall P, Hastings E, et al. The new NHGRI-EBI

Catalog of published genome-wide association studies (GWAS Catalog). Nucleic Acids Res. 2017 Jan 1;45(D1):D896–901.

22. Allen M, Wang X, Serie DJ, Strickland SL, Burgess JD, Koga S, et al. Divergent brain gene expression patterns associate with distinct cell-specific tau neuropathology traits in progressive supranuclear palsy. Acta Neuropathol. 2018 Nov 22;136(5):709–27.

23. Allen M, Carrasquillo MM, Funk C, Heavner BD, Zou F, Younkin CS, et al. Data Descriptor: Human whole genome genotype and transcriptome data for Alzheimer's and other neurodegenerative diseases. 2016;

24. Vaquerizas JM, Kummerfeld SK, Teichmann SA, Luscombe NM. A census of human transcription factors: Function, expression and evolution. Nat Rev Genet. 2009;10(4):252–63.

1. Abbreviations

**Abbreviation**	**Term**
(q)PCR	(Quantitative) polymerase chain reaction
AD	Alzheimer's Disease
ALS	Amyotrophic Lateral Sclerosis
Ast	Astrocytes
ATAC-seq	Assay for Transposase-Accessible Chromatin using sequencing
BH	Benjamini-Hochberg
bp	Base pairs
BP	biological process
CBD	Corticobasal Degeneration
CC	cellular compartment
CMA	Chaperon-mediated autophagy
CRE	*Cis*-regulatory element
DAR	Differentially accessible region
DEG	Differentially expressed gene
DNA	Desoxyribonucleic acid
FDR	False discovery rate
FTD	Frontotemporal Dementia
GA	Gene accessibility
Gb	Giga bases
GO	Gene ontology

GSEA	Gene-set enrichment analysis
GWAS	Genome-wide association study
kNN	k-nearest neighbor
LSI	latent semantic indexing
LBD	Lewy Body Dementia
Lime	Local interpretable model-agnostic explanations
Log2-FC	Binary logarithm fold-change
MF	molecular function
ML	Machine learning
MSA	Multiple System Atrophy
PCA	Principle component analysis
PD	Parkinson Disease
PMI	*Post mortal* interval
PSP	Progressive Supranuclear Palsy
pTau	Hyperphosphorylated Tau
RAP	Regulon activity profile
RNA-seq	Ribonucleotide acid sequencing
RTN	Reconstruction of transcriptional regulatory networks
sn*	Single nulcei
TA	Tufted astrocyte
TF(M)(E)	Transcription factor (motif) (enrichment)
UMAP	Uniform Manifold Approximation and Projection
UPR	Unfolded protein response
UPS	Ubiquitin proteasome system
XGB	Extreme gradient boosting
